# A Bioinformatics Whole-Genome Sequencing Workflow for Clinical Mycobacterium tuberculosis Complex Isolate Analysis, Validated Using a Reference Collection Extensively Characterized with Conventional Methods and *In Silico* Approaches

**DOI:** 10.1128/JCM.00202-21

**Published:** 2021-05-19

**Authors:** Bert Bogaerts, Thomas Delcourt, Karine Soetaert, Samira Boarbi, Pieter-Jan Ceyssens, Raf Winand, Julien Van Braekel, Sigrid C. J. De Keersmaecker, Nancy H. C. Roosens, Kathleen Marchal, Vanessa Mathys, Kevin Vanneste

**Affiliations:** a Transversal Activities in Applied Genomics, Sciensano, Brussels, Belgium; b Bacterial Diseases, Sciensano, Brussels, Belgium; c Department of Information Technology, Internet Technology and Data Science Lab (IDLab), Interuniversity Microelectronics Centre (IMEC), Ghent University, Ghent, Belgium; d Department of Plant Biotechnology and Bioinformatics, Ghent University, Ghent, Belgium; e Department of Genetics, University of Pretoria, Pretoria, South Africa; University of Iowa College of Medicine

**Keywords:** *Mycobacterium tuberculosis*, whole genome sequencing, validation, public health, national reference center, single nucleotide polymorphism, antimicrobial resistance

## Abstract

The use of whole-genome sequencing (WGS) for routine typing of bacterial isolates has increased substantially in recent years. For Mycobacterium tuberculosis (MTB), in particular, WGS has the benefit of drastically reducing the time required to generate results compared to most conventional phenotypic methods. Consequently, a multitude of solutions for analyzing WGS MTB data have been developed, but their successful integration in clinical and national reference laboratories is hindered by the requirement for their validation, for which a consensus framework is still largely absent. We developed a bioinformatics workflow for (Illumina) WGS-based routine typing of MTB complex (MTBC) member isolates allowing complete characterization, including (sub)species confirmation and identification (16S, *csb*/RD, *hsp65*), single nucleotide polymorphism (SNP)-based antimicrobial resistance (AMR) prediction, and pathogen typing (spoligotyping, SNP barcoding, and core genome multilocus sequence typing). Workflow performance was validated on a per-assay basis using a collection of 238 in-house-sequenced MTBC isolates, extensively characterized with conventional molecular biology-based approaches supplemented with public data. For SNP-based AMR prediction, results from molecular genotyping methods were supplemented with *in silico* modified data sets, allowing us to greatly increase the set of evaluated mutations. The workflow demonstrated very high performance with performance metrics of >99% for all assays, except for spoligotyping, where sensitivity dropped to ∼90%. The validation framework for our WGS-based bioinformatics workflow can aid in the standardization of bioinformatics tools by the MTB community and other SNP-based applications regardless of the targeted pathogen(s). The bioinformatics workflow is available for academic and nonprofit use through the Galaxy instance of our institute at https://galaxy.sciensano.be.

## INTRODUCTION

Technological advances in next-generation sequencing (NGS), improved bioinformatics methods, and dropping costs, have contributed to rendering whole-genome sequencing (WGS) an increasingly popular alternative to classically employed molecular approaches for routine typing of bacterial isolates. The added value of WGS in routine pathogen surveillance and outbreak situations has been illustrated extensively for multiple pathogens (1–3). In contrast to most molecular methods, WGS provides an “all-in-one” solution including, among other things, the detection of genes of interest (e.g., antimicrobial resistance [AMR] genes and virulence factors) and single nucleotide polymorphisms (SNPs) that can resolve the relatedness between isolates, eliminating the need for multiple sequential laborious molecular assays (4). The parallel nature of most modern sequencing technologies has facilitated multiplexing approaches, resulting in very high throughputs.

In recent years, many solutions for analyzing Mycobacterium tuberculosis (MTB) WGS data have been developed to replace traditionally employed molecular approaches (5–8). This is facilitated by the well-characterized MTB genome, rendering it well suited for WGS analyses (9). Because this pathogen evolves at a slow pace (10), many approaches focus on identifying SNPs against the H37Rv reference genome (11) and can be broadly grouped into three different, but still intertwined, functionalities. First, species confirmation and identification of the correct (sub)lineage are crucial, as lineages vary in virulence, transmissibility, geographical occurrence, host response, and emergence of drug resistance (12). Several approaches are used, with some of them specific to *Mycobacterium* species, such as heat shock protein 65 (*hsp65*) species differentiation (13), and genomic markers including the *csb* gene and the regions of difference (RDs) (14, 15). Species-agnostic methods such as 16S rRNA sequencing are generally applicable to bacterial pathogens, including MTB (16). Information for all these assays of interest can be obtained through a single WGS run, and WGS has proved itself a viable alternative for species confirmation and identification (12, 17). These WGS-based approaches often rely on the detection of specific SNPs to discriminate known MTB complex (MTBC) (sub)lineages (12, 17–20). Second, since multidrug-resistant (MDR) and extensively drug-resistant (XDR) strains are increasing in prevalence, rapid and accurate AMR characterization is required for successful treatment and monitoring (21). Traditionally, phenotypic antibiotic susceptibility testing in mycobacteria is performed with a culture step in mycobacterial growth indicator tubes (MGIT), followed by solid drug susceptibility testing (DST) or broth microdilution, which can take an especially long time due to the slow growth rate (22). In contrast to the majority of bacterial pathogens, AMR in MTB is mainly conferred by point mutations and, to a lesser degree, indels in genes coding for proteins targeted by drugs (23). Genotypic AMR prediction is therefore traditionally performed using Sanger sequencing or line probe assays (LPAs) on a limited target set. In contrast, WGS can screen the entire genome, thereby eliminating the need for different experiments for separate genomic loci. Many bioinformatics AMR detection workflows are available (19, 23–25) but have been shown to produce variable results, caused by differences in the employed underlying databases of AMR-associated mutations and variant-calling methodologies (26). Third, pathogen typing allows evaluating relatedness of isolates to make epidemiological links. Mycobacterial interspersed repetitive unit–variable number tandem repeat (MIRU-VNTR) typing and spacer oligonucleotide typing (spoligotyping) are two of the most widely used molecular methods to determine relatedness between MTBC isolates. However, systematic comparisons show that WGS-based approaches such as SNP typing or core genome multilocus sequence typing (cgMLST) can provide more discriminatory power (27). Similar to the detection of AMR mutations, differences in bioinformatics workflows can affect the classification of strains into transmission clusters, which are usually defined by a threshold for the maximum number of pairwise SNP or allele differences (26, 28). Backward compatibility of WGS with molecular spoligotyping is facilitated through *in silico* spoligotyping methods that enable comparison with historical data (29, 30).

Despite the many advantages of using WGS over classical methods, integration into routine pathogen typing poses additional challenges. For smaller laboratories that lack the necessary infrastructure and/or bioinformatics expertise, WGS data analysis remains a bottleneck, highlighting the need for user-friendly pipelines (31). Moreover, integration into a quality system for routine use is complicated by the lack of standardization between laboratories and the lack of proper validation of bioinformatics workflows to demonstrate adequate performance, which is a prerequisite for most clinical and national reference centers (NRCs) (32–34). We previously proposed a validation framework and used it to exhaustively characterize the performance of bioinformatics workflows to analyze WGS data for the pathogens Neisseria meningitidis (35) and Shiga-toxin-producing Escherichia coli (STEC) (36), demonstrating that bioinformatics WGS workflows can achieve high performance to replace traditional approaches for routine pathogen typing. This framework uses assay-specific definitions to determine performance metrics, adopted from classical molecular assays. However, except for PointFinder (37), all assays of the aforementioned workflows were based on detecting specific genes or alleles, and the frameworks are therefore not directly applicable for evaluating the performance of bioinformatics assays for MTBC members, since most assays of critical clinical relevance are based on the identification of SNPs. Although the PointFinder assay is SNP-based, it was evaluated using a phenotypic reference standard in the validation of the aforementioned STEC workflow, and not through validating the correct identification of the underlying SNPs (36). A validation framework for evaluating bioinformatics assays for MTBC members that properly considers the detection and identification of particular SNPs is, however, not yet established. Validation of SNPs is particularly challenging due to both technological and analytical limitations and to the lack of widely accepted guidelines for evaluating methods for calling SNPs and indels (38). This is aggravated even further by the fact that differences in tools, parameters, and databases can all affect SNP calling and filtering and therefore impact downstream analysis results (39).

Here, we present a bioinformatics workflow for routine typing of MTBC members, allowing complete pathogen characterization based on different bioinformatics assays such as (sub)species confirmation and identification (including 16S rRNA, *hsp65*, *csb*, and RDs), SNP-based AMR prediction, and (sub)lineage detection (including spoligotyping, cgMLST, and SNP barcoding). In particular, we present an updated and extended validation framework for a WGS-based bioinformatics workflow incorporating several bioinformatics assays that rely on correct SNP detection, which can help with standardization of bioinformatics tools across the global MTBC community and other SNP-based applications regardless of the targeted pathogen(s).

## MATERIALS AND METHODS

### Bioinformatics workflow.

**FIG 1:**
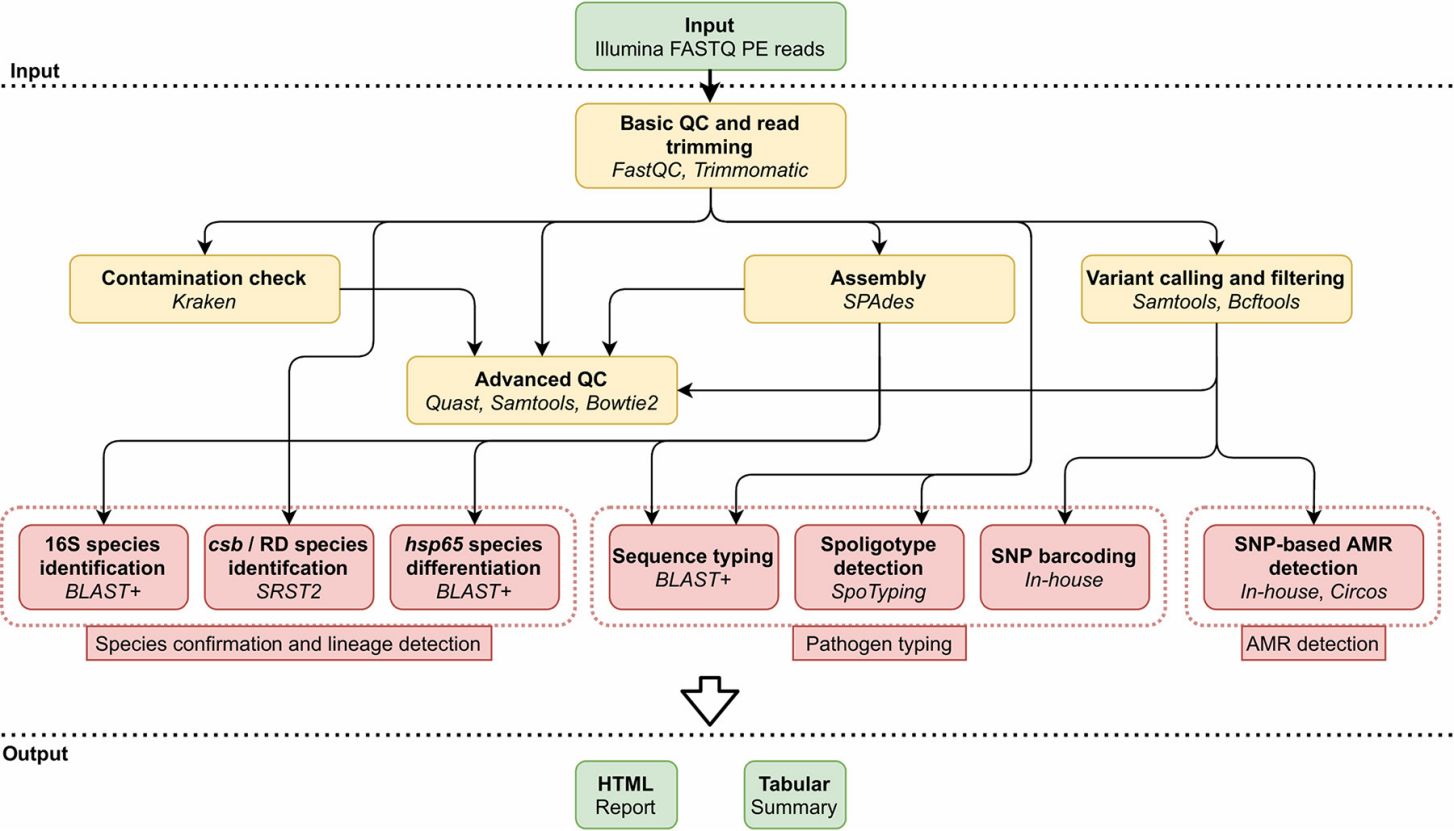
Overview of the bioinformatics workflow. Each box represents a component corresponding to a series of tasks that provide a certain well-defined functionality (indicated in bold). The major bioinformatics utilities employed in each module are also mentioned (indicated in italics). Data processing steps are indicated in yellow, bioinformatics assays are indicated in red, and the dotted red boxes group the assays into the three main categories. PE, paired-end; QC, quality control; RD, regions of difference; AMR, antimicrobial resistance.

**Data (pre)processing and quality control.** Figure 1 provides an overview of the bioinformatics workflow, which is compatible with all WGS data generated using the Illumina technology. Data preprocessing and quality control are executed as described in Bogaerts et al. (36) and detailed in the supplemental material. Several quality metrics are then computed, for which warning and failure thresholds were defined by selecting more and less stringent values for metrics exhibiting less and more variation between samples, respectively, and for which an overview is presented in Table 1. When the input data fails one of the quality control (QC) checks, a warning is added to the report, but the full output is generated and the decision on whether or not to resequence is left to the end user.

**TABLE 1:** Employed QC metrics

Metric	Definition	Warning threshold	Failure threshold
Contamination (%)	Percentage of reads classified as highest-occurring species other than M. tuberculosis	1.00	5.00
Median coverage against reference genome (×)	Median coverage based on mapping of the trimmed reads against the H37Rv reference genome	20	10
Reads mapping back to reference genome (%)	Percentage of the trimmed reads mapping back to the H37Rv reference genome	95	90
cgMLST genes identified (%)	Percentage of cgMLST genes identified. Only perfect hits (i.e., full length and 100% identity) are considered	95	90
Average read quality (Q-score)	Q-score of the trimmed reads averaged over all reads and positions	30	25
GC content deviation (%)	Deviation of the average GC content of the trimmed reads from the expected value for M. tuberculosis (65.5% [11])	2.00	4.00
N-fraction	Average N-fraction per read position of the trimmed reads	0.05	0.10
Per base sequence content (%)	Difference between AT and GC frequencies averaged at every read position. Since primer artefacts can cause fluctuations at the start of reads due to the nonrandom nature of enzymatic tagmentation when the Nextera XT protocol is used for library preparation, the first 20 bases are not included in this test. As fluctuations can also exist at the end of reads caused by the low abundance of very long reads because of read trimming, the 0.5% longest reads are similarly excluded.	3.00	6.00
Minimum read length (%)	Minimum read length after trimming (denoted as percentage of untrimmed read length) that minimum half of all trimmed reads must obtain (e.g., half of all trimmed reads should either be minimally 120 or 200 bases long when raw input reads lengths are 300 bases long)	66.67	40.00

For variant calling and filtering, processed reads are mapped against the H37Rv reference genome for MTB (Ensembl accession number ASM19595v2) using Bowtie 2 2.3.0 (40), after which a pileup is generated using the SAMtools “mpileup” function setting the following options: “--count-orphans” and “--VCF.” The coverage is estimated by calculating the median depth across all genomic positions using SAMtools depth 1.9. Variants are called using the BCFtools 1.9 function “call” (41) with the “--ploidy” option set to 1 and the “--consensus-caller” option enabled, after which low-quality variants are removed using the BCFtools “filter” function to enforce quality criteria adopted from the CSI phylogeny pipeline (42): a minimum SNP quality of 25, a minimum mapping quality of 30, covered by at least 10 reads, of which at least one is forward and reverse. A sliding window approach is then applied to remove variants located within 10 nucleotides of each other, and variants with a Z‐score lower than 1.96 and/or a Y‐multiplier of lower than 10 are also removed (see reference 42). Variants located in regions problematic for variant calling (e.g., repeat regions) are also removed, for which the list of excluded loci from the unified analysis variant pipeline of ReSeqTB is used (19) (https://github.com/CPTR-ReSeqTB/UVP).

**(Sub)species confirmation and identification.** (Sub)species confirmation and identification consist of three assays that complement each other and provide clinical and NRC experts with the necessary information to assess the taxonomic origin of the isolate. First, the genome assembly is aligned to the NCBI 16S rRNA database (downloaded on 27 November 2017) using blastn 2.6.0 with default parameters and filtering out hits with less than 95% identity or 60% coverage. The top five hits ordered by e-value after filtering are included in the output. Second, the best matching sequence from a database with 157 (partial) *hsp65* sequences (43) is selected using the same algorithmic approach as for 16S rRNA, and the best hit is selected with the allele scoring method described by Larsen et al. (44). Third, the presence and/or absence of the *csb* gene, RD1, and RD9 are determined. The *csb* gene is absent in bovine species but present in nonbovine species, while RD1 is absent in M. bovis BCG but present in other bovine species, and RD9 is absent in M. africanum but present in other MTBC members (see Fig. S1 in the supplemental material). Since BLAST+-based detection often experiences issues with detecting the long RD1 (10,956 bp) and RD9 (2,032 bp) sequences due to contig fragmentation, SRST2 0.2.0 (45) is used. The trimmed forward and reverse reads are provided as input, and the best hit is selected by SRST2. Output is presented as a decision tree with taxonomic predictions highlighted in green, an example of which is provided in Fig. S1.

**SNP-based antimicrobial resistance detection.** A BED file containing regions associated with AMR (Table S1) is used to extract variants from the unfiltered VCF file using BCFtools filter 1.9. Afterward, BCFtools csq 1.9 (46) is used to annotate the resulting VCF file with mutational effects. The reference FASTA file is provided with the “--fasta-ref” option, and the corresponding GFF Ensembl annotation is provided with the “--gff-annot” option. Annotated mutations are then parsed to report nucleotide, amino acid, frameshift, promoter, and stop codon changes (other types of mutations are classified as unknown). Databases were constructed from in-house resources and three literature sources (21, 47, 48) and have been made available as tabular files in Zenodo (http://doi.org/10.5281/zenodo.4434636). Each database contains mutations and their associated phenotypes (sensitive or resistant) to a particular or multiple antibiotic(s). Output is provided as separate tables for filtered and unfiltered variants that list the nucleotide change(s), mutational effects, counts for each of the four nucleotides (when applicable), and the corresponding association(s) with AMR to any antibiotic(s). In case of multiple database entries for a single mutation, all are listed. For mutations classified as unknown, antibiotics associated with the region are listed if available. Interpretation of mixed mutations is left to the end user, who can decide on heteroresistance based on the nucleotide counts at the corresponding position. Output is provided as a table of all antibiotics and the predicted AMR phenotype. Samples are predicted as resistant to an antibiotic if at least one mutation associated with a “known resistant” phenotype is detected. Based on predicted resistance(s), samples are classified as not resistant, mono-resistant, MDR, pre-XDR, XDR, or other (49). A visualization is created dynamically with Circos 0.69-6 (50) to visualize coverage across the chromosome and highlight regions that contain mutations associated with a known resistant phenotype, for which examples are provided in Fig. S2. The workflow also detects compensatory mutations that alleviate loss of fitness produced by drug resistance‐associated mutations by using the same approach as described for AMR-associated mutations (49, 51).

**Pathogen typing.** Three assays are included. First, SpoTyping 2.1 (29) is used to determine the spoligotype *in silico*, setting the threshold values for the “--min” and “--rmin” parameters dynamically to 10% of the estimated coverage against the H37Rv reference genome rounded to the nearest integer with a minimum value of 3. Samples with an estimated coverage of >50× are first downsampled with the seqtk 1.2 sample function to approximately 50×. SpoTyping is then executed on trimmed paired-end reads setting the “--swift” option to “off.” Output is provided as the spoligotyping binary and octal representations and the number of occurrences of the spacer sequence in the reads for all 43 spacers. The detected spoligotype is also queried against the SpolDB4 database (52), which contains the common name, shared international type (SIT) number, and number of isolates with matching spoligotype, which are all also provided in the output. Second, the SNP barcode assay developed by Coll et al. (12) is implemented to cross-reference SNPs with the database provided by them. At each of the barcoding levels, the most likely lineage is selected based on the number of supporting SNPs. If no SNPs are detected at the targeted positions, the sample is classified as lineage 4.9, corresponding to the clade containing the H37Rv reference genome. Output is provided as lineage name, main spoligotype, RD classification, number of supporting SNPs, and an overview of all detected SNPs from the database. Third, cgMLST is evaluated using the respective databases hosted by PubMLST (http://pubMLST.org) (53), for which all sequences and profiles are obtained using the REST API (86) and are automatically pulled in-house and updated weekly (the date of the last database update is included in the output). cgMLST allele calling is executed as described in Bogaerts et al. (35).

**Implementation and availability.** The workflow was implemented in Python 3.7.5 and tested on an Ubuntu 18.04 (64-bit) server. Workflow output is provided as an interactive HTML report with the relevant information and links to the full output of the different bioinformatics assays, enabling further processing or in-depth investigation. The tabular summary file contains an accumulation of the most important statistics and results in tab-separated format for programmatic processing. The workflow was integrated as a stand-alone tool in an in-house Galaxy workflow management system instance (54). This “push-button” workflow is also available at the public Galaxy instance of our institute at https://galaxy.sciensano.be as a free resource for academic and nonprofit use (registration required). Use of the workflow through Galaxy is explained in a training video that is available on YouTube (https://www.youtube.com/watch?v=cOtyNfsWJi8). A screenshot of the interface is provided in Fig. S3. Besides the validated assays discussed in this article, it also includes other bioinformatics assays not validated for routine purposes but useful for informative purposes, such as detection of AMR-associated mutations with PointFinder (37), *in silico* screening of 51 informative SNPs to delineate principle genetic groups and SNP cluster groups (55), subspecies and lineage identification with SNP-IT (17), and regular MLST sequence typing with the PubMLST scheme. Direct read mapping with SRST2 and kmer-based detection of genes and alleles with KMA (56) are also supported.

### Validation data set and characterization with conventional methods.

**Selection of samples and WGS.** The validation data set was constructed by selecting samples from the routine activities sent to the Belgian NRC until a total of 238 samples were collected for which WGS data did not fail any of the quality control (QC) metrics listed in Table 1. The strains were sampled from humans and were analyzed with NGS in the context of various research projects and integration of WGS in the NRC routine activities. Data from molecular testing was available from routine diagnostics, including species identification, spoligotyping, DST in MGIT, and genotyping of AMR mutations. The diversity of the in-house samples included in the validation data set was assessed by constructing a SNP-based maximum likelihood phylogeny using the filtered VCF files generated by the workflow (see supplemental material). Information on the lineage, SIT, and number of detected and inserted AMR mutations was added as annotations and visualized using iTOL (57).

Samples were extracted by first inactivating a volume of 1 ml of the identified mycobacterial culture for 15 min at 95°C and then treating the culture by bead-beating 3 × 30 sec with 0.5 mm zirconia/silica beads (BioSpec) using the Mini-BeadBeater-16 (BioSpec). The pellet collected after centrifugation (2 min at 13,000 × *g*) was used for the DNA extraction outside the biosafety level 3 (BSL3) lab using the MagCore genomic DNA bacterial kit. After extraction, the amount of DNA was quantified using the Quantus fluorometer for sensitive detection of nucleic acids (detection limit of 10 pg/μl) using QuantiFluor dye system (QuantiFluor double-stranded [dsDNA] system). WGS was performed as an ISO 17025 accredited assay. Here, Nextera XT libraries (Illumina, San Diego, CA) were constructed with a 15-cycle PCR indexing step as described by Shea et al. (7). First, 1 ng of MTB genomic DNA was used as input for tagmentation, unless this amount was not available, in which case, 5 μl of the genomic DNA extract was used. The concentration of the samples was measured before the WGS run using the high-sensitivity dsDNA Qubit kit (Thermo Fisher Scientific, Waltham, MA, USA), and the majority of samples had a DNA concentration of ≤0.010 ng/μl. Libraries were pooled aiming for a theoretical coverage of at least 30× and subsequently sequenced on a MiSeq instrument (Illumina, San Diego, CA) using the MiSeq V3 chemistry following the manufacturer’s instructions, for the production of 2 × 250-bp paired-end reads. A negative control (Tris-HCl [10 mM, pH 8.5] with 0.1% Tween 20 instead of MTB DNA as the template for tagmentation) was included in each library preparation and on each sequencing run.

**FIG 2:**
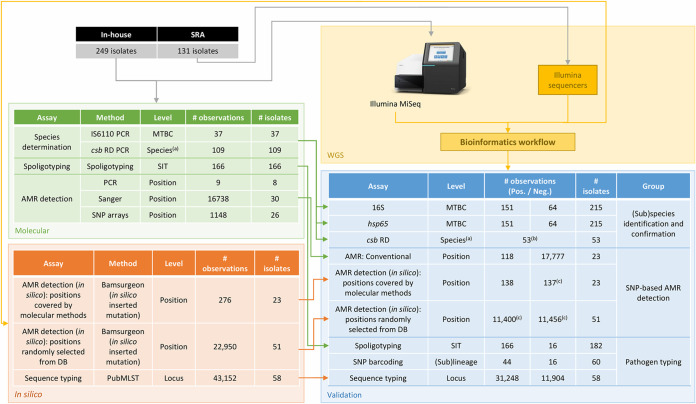
Schematic representation of the validation strategy. The colored boxes represent the different components of the validation strategy as follows: whole-genome sequencing (WGS, yellow), characterization with molecular methods (molecular, green), *in silico* modification or characterization of WGS data (*in silico*, orange), and validation of the assays (validation, blue). Arrows indicate the flow of the data between the different steps. The “Method” columns represent the molecular or bioinformatics method that was used to generate data. The “# observations” columns correspond to the number of observations that were obtained or evaluated at the listed level. Abbreviations: MTBC, Mycobacterium tuberculosis complex; SIT, shared international type; RD, regions of difference; WGS, whole-genome sequencing; SRA Sequence Read Archive; AMR, antimicrobial resistance; LPAs line probe assays. ^(a)^ csb/RD allows distinguishing the following 4 species within the MTBC: *Mycobacterium tuberculosis, Mycobacterium africanum, Mycobacterium bovis* BCG, and *Mycobacterium bovis*. ^(b)^ Validation was performed on a per-species basis reusing the positive observations of the other species as negative controls. ^(c)^ Some observations of the *in silico* modified dataset were removed because of incompatibilities with preexisting mutations in the WGS data.

All in-house-generated sequencing data have been submitted to the SRA (58) under BioProject number PRJNA681718. For the validation of some assays, the data set was extended with Illumina data collected from the SRA as described in the following sections. The accession numbers for the 380 samples used in the validation are listed in Table S2. A schematic overview of characterization with conventional methods is provided in Fig. 2.

**(Sub)species identification and confirmation.** At the NRC, species determination was performed by PCR of the IS*6110* insertion element for strains analyzed before 2018 (59). From 2018 onward, PCR of the *csb* gene was performed to distinguish between bovine and nonbovine strains, and an additional PCR of the RD1 was performed for the bovine strains to distinguish between M. bovis and M. bovis BCG (14, 60). As our in-house collection contained mainly M. tuberculosis isolates and *Mycobacterium* samples with inconclusive species information, the data set was extended with data sets from different subspecies retrieved from the SRA. This data set was further extended with 16 negative control samples from species outside the *Mycobacterium* genus for the 16S rRNA and *hsp65* assays (Table S3). The truth set for all three assays (16S rRNA, *hsp65*, and *csb*/RD) consisted of the taxonomic information for these samples either obtained as described above for the in-house-sequenced samples or retrieved from SRA metadata for the public data sets.

**SNP-based antimicrobial resistance detection.** Molecular AMR detection was performed in case of suspected resistance by MGIT DST. Detection of AMR mutations was performed using the GenoType MTBBR+ and MTBDRsl LPA kits (Hain Lifescience), multiplex PCR for isoniazid resistance (61), or Sanger sequencing on a Genetic Analyzer 3500 (Applied Biosystems) of one or more genes associated with AMR (62). Results per sample are provided in Table S4 (phenotypic testing), Table S5 (PCR and LPAs), and Table S6 (Sanger sequencing). Because only a relatively small subset of mutations present in the database was covered by molecular methods, the range of evaluated mutations was extended by inserting variants *in silico* into the actual WGS data sets. Data sets were modified by first mapping the reads to the M. tuberculosis H37Rv reference genome (NCBI accession number GCA_000195955.2) using the Burrows-Wheeler Aligner MEM algorithm (BWA-MEM) 0.7.15 with the “-M” option enabled (63). Secondary alignments were removed from the resulting BAM files using SAMtools view 1.3.1 with the “-h” option enabled and “-F” set to “0 × 900” and then sorted and indexed using SAMtools sort 1.3.1 and SAMtools 1.3.1 index. Nucleotide mutations were inserted into indexed BAM files using Bamsurgeon 1.2 (64) with the “--ignorepileup” and “--force” options enabled, “-p” set to 5, “--coverdiff” set to 0.01, “--mindepth” set to 1, and “--minmutreads” set to 1. The resulting BAM files were sorted using SAMtools sort with the “-n” option and converted to fastq.gz format using BEDTools bamtofastq 2.25.0 (65) and gzip 1.6. Finally, modified positions were confirmed with bam-readcount 0.8.0 (https://github.com/genome/bam-readcount).

**Pathogen typing.** Routine typing of MTB strains in the NRC was performed by a combination of spoligotyping and MIRU-VNTR. Clusters were subsequently detected using Bionumerics as described previously (66). This data set was complemented with the same 16 negative-control samples from species outside the *Mycobacterium* genus used for species identification (Table S7), which were also used as negative controls for SNP-barcoding (Table S8) and sequence typing (Table S9).

SNP-barcoding assay performance was evaluated on 44 MTBC samples with lineage information available selected from the study describing the lineage classification (12). Accession numbers are provided in Table S8. This selection covered all six major lineages and 44 out of 55 sublineages.

For the sequence typing assay, a representative subset of in-house-sequenced samples was selected by collapsing the branches from the phylogeny described in “Selection of Samples and WGS” on 40 or fewer allele differences. Afterward, the validation data set was created by randomly selecting a sample from each distinct group for a total of 42 samples. An overview of the selected samples is provided in Table S9.

### Validation of the bioinformatics workflow.

**Validation strategy.** We used a previously described validation framework with performance metrics adapted for exhaustively validating the bioinformatics workflow as follows: repeatability, reproducibility, accuracy, precision, sensitivity, and specificity (35, 36). The validation strategy is schematically represented in Fig. 2, and a full overview of all performance metrics and their corresponding definitions and formulas is presented in Table 2. Workflow repeatability and reproducibility were evaluated by running the bioinformatics workflow twice on the same data set on the same and a separate computational environment, respectively. The two computational environments were Python 3.7.5 and Python 3.7.4 on two different Ubuntu 18.04.3 LTS (64-bit) servers. Accuracy, precision, sensitivity, and specificity all require classification of workflow results as either true positives (TPs), false positives (FPs), true negatives (TNs), or false negatives (FN), determined from comparison against a reference that represents the “ground truth.” Information for the reference ground truth is provided in the following sections per assay. The validation was limited to data sets that passed the various quality control checks included in the bioinformatics workflow (i.e., no fail for any of the QC checks; Table 1). For negative control samples from species outside the MTBC, results of QC checks aiming to detect contamination or specific to M. tuberculosis were disregarded (cgMLST genes detected, percentage GC content, contamination check with Kraken, percentage of reads mapping back to the M. tuberculosis reference genome, and median genome coverage).

**TABLE 2:** Evaluated performance metrics and their corresponding definitions and formulas^*a*^

Metric	Definition	Formula	Assay-specific definitions	Bioinformatics assay								
				(Sub)species identification and confirmation			SNP-based antimicrobial resistance detection			Pathogen typing		
				16S	*csb* RD	*hsp65*	Conventional	*In silico* conventional	*In silico* database	Spoligotyping	SNP barcoding	Sequence typing
Repeatability	Agreement of the assay based on intra-assay replicates	Repeatability = 100% × (no. of intra-assay replicates in agreement)/(total no. of intra-assay replicates)	Intra-assay replicate	Running the bioinformatics workflow twice on the same data set on the same computational environment								
Reproducibility	Agreement of the assay based on interassay replicates	Reproducibility = 100% × (no. interassay replicates in agreement)/(total no. interassay replicates)	Interassay replicate	Running the bioinformatics workflow twice on the same data set on a separate computational environment								
Accuracy	The likelihood that results of the assay are correct	Accuracy = 100% × (TP + TN)/(TN + FN + TP + FP)	True-positive result (TP)	Detection of MTBC species in positive sample	Targeted species detected in positive sample	Detection of MTBC species in positive sample	Detection of expected variant			Detection of expected spoligotype	Expected lineage in detected lineages	Detection of the same allele as in the reference standard
Precision	The likelihood that detected results of the assay are truly present	Precision = 100% × TP/(TP + FP)	False-negative result (FN)	No detection of MTBC species in positive sample	Targeted species not detected in positive sample	No detection of MTBC species in positive sample	No detection of expected variant			No detection of spoligotype or detection of wrong spoligotype	Expected lineage not in detected lineages	Detection of a different allele as in the reference standard
Sensitivity	The likelihood that a result will be correctly picked up by the assay when present	Sensitivity = 100% × TP/(TP + FN)	True-negative result (TN)	No detection of MTBC species in negative sample	Targeted species not detected in negative sample	No detection of MTBC species in negative sample	No variant detected at WT position			No detection of spoligotype when none expected	No detection of lineage when none expected	No detection of an allele in negative-control sample
Specificity	The likelihood that a result will not be falsely picked up by the assay when not present	Specificity = 100% × TN/(TN + FP)	False-positive result (FP)	Detection of MTBC species in negative sample	Targeted species detected in negative sample	Detection of MTBC species in negative sample	Detection of nonexisting variant or other variant than expected			Detection of spoligotype when none expected	Detection of lineage when none expected	Detection of an allele in negative-control sample

a TP, true positive; FP, false positive, TN, true negative; FN, false negative; WT, wild-type.

**(Sub)species identification and confirmation.** For the validation of the 16S rRNA and *hsp65* assays, the validation data set was divided into a positive set of 151 samples from species within the MTBC and a negative set of 64 samples from species outside the MTBC (within and outside the *Mycobacterium* genus). An overview is provided in Table S3. Validation was performed at the level of the MTBC using the following definitions: TP, samples from the positive set with at least one reported hit for a species belonging to the MTBC; FN, samples from the positive set with no reported hit for a species belonging to the MTBC; TN, samples from the negative set with no hit for a species belonging to the MTBC; FP, samples from the negative set with at least one reported hit for a species belonging to the MTBC. For the 16S assay, hits to incomplete 16S reference sequences were discarded.

For the *csb*/RD assay, the validation data set consisted of 53 samples from the 4 species that can be distinguished with this assay as follows: M. tuberculosis (*n* = 12), M. africanum (*n* = 12), M. bovis BCG (*n* = 13), and M. bovis (*n* = 16), for which an overview is provided in Table S10. Validation was performed on a per-species basis, with the samples from the corresponding species as the positive set and samples from the three other species as the negative set. The following definitions were used: TP, samples from the positive set correctly identified as the target species; FN, samples from the positive set not correctly identified as the target species; TN, samples from the negative set not identified as the target species; FP, samples from the negative set identified as the target species.

**FIG 3:**
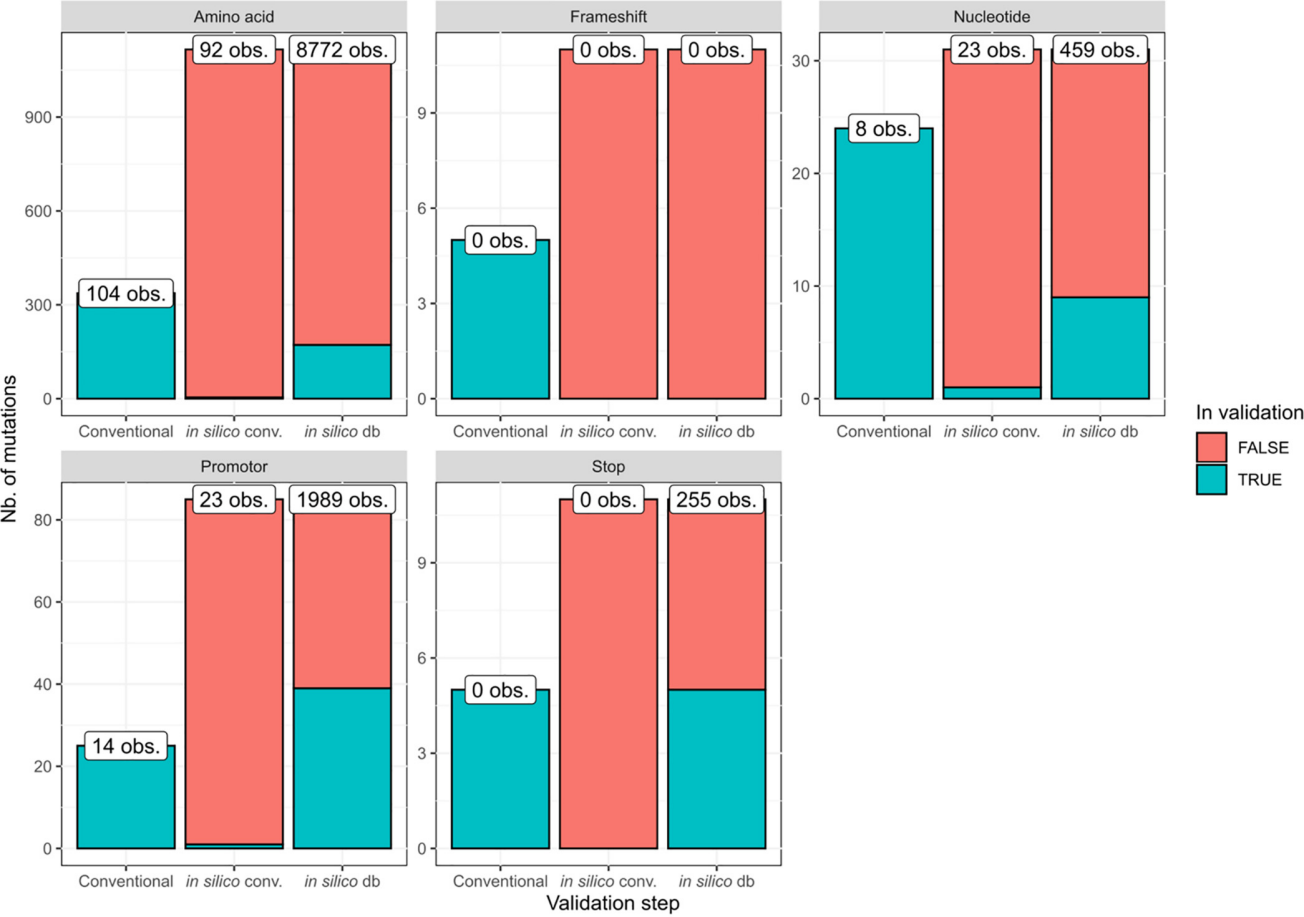
Overview of the mutations included in the (validation of) SNP-based AMR detection. The *y* axis represents the number of mutations for the validation steps listed on the *x* axis. Each subplot represents a different category of mutations. The colors indicate if mutations were included (turquoise) or not included (red) in the corresponding validation step. The total height of each bar (i.e., red + turquoise) corresponds to the maximum number of mutations of the corresponding category that could be included in the validation. For the comparison with conventional methods, this corresponds to all mutations located in genomic regions targeted by the conventional methods. For the WGS-based workflow (validated through *in silico* conventional and *in silico* database), this corresponds to the total number of AMR mutations in the database. The white labels show the number of times mutations of the corresponding category were present in the validation samples according to the reference information (note that this number can be higher than the number of mutations on the *y* axis because the same mutation can have been validated multiple times in different samples). This figure demonstrates that (i) the number of mutations that are screened with WGS far exceeds the mutations detected with conventional molecular methods, and (ii) the *in silico* approach enables us to validate a much larger fraction of mutations in the database. obs., observations; db, database; conv. conventional; AMR, antimicrobial resistance.

**SNP-based antimicrobial resistance detection.** The SNP-based AMR detection assay was validated at the genotypic level in three steps. The number of observations and included mutations for each mutation type and validation step are shown in Fig. 3. First, correspondence between workflow output and AMR mutations confirmed with molecular methods (PCR, Sanger sequencing, and LPAs) was evaluated. Full results for AMR screening with PCR and LPAs (Table S5) and Sanger sequencing (Table S6) are provided in the supplemental material. Note that only a subset of phenotypically tested samples (Table S4) was also screened genotypically for AMR mutations with conventional methods (Tables S5 and S6). Validation was performed at the level of individual mutations, considering confirmed mutations as the positive set and positions in the database confirmed as being the wild-type (WT) by molecular methods as the negative set. The following definitions for classification were used for all three steps: TP, mutations from the positive set correctly identified by the workflow; FN, mutations from the positive set incorrectly identified as the WT by the workflow; TN, positions from the negative set correctly identified as the WT by the workflow; FP, positions from the negative set incorrectly identified as mutations by the workflow.

Second, to assess equivalence between the first approach and an *in silico* approach simulating randomly selected mutations from the database (including both mutations associated with phenotypic susceptibility and resistance) in the actual sequencing data sets, an additional data set was generated, consisting of mutations that were inserted *in silico* at positions covered by the molecular methods in samples with no prior reference data from molecular methods. In-house-sequenced samples (*n* = 23) from species within the MTBC were used (see Table S11). The six randomly selected mutations from the positions covered by molecular methods are listed in Table S12 and comprise amino-acid changes (*n* = 4), promoter (*n* = 1), and nucleotide mutations (*n* = 1). The positive set was constructed by inserting the selected mutations in raw reads of sequencing data sets. The negative set was constructed by modifying raw reads of sequencing data sets to ensure the presence of the WT at the targeted positions. This resulted in a positive and negative set of each 138 observations (i.e., 6 mutations times 23 samples).

Third, to expand the range of evaluated positions since only a subset of positions could be evaluated by using molecular methods, randomly selected mutations from the database of AMR mutations used by the workflow were inserted *in silico* in raw reads of sequencing data sets. In-house-sequenced samples (*n* = 51) from species within the MTBC were used (see Table S13). The positive set was created by randomly selecting mutations from the entire database covering a broad range of antibiotics (Table S14) and inserting them *in silico* into sequencing data sets at a read depth of 100% as described above. This also enabled evaluating performance on mutations that are not commonly observed by the NRC, such as those associated with resistance to newer antibiotics, including bedaquiline and linezolid. Frameshift mutations were not compatible with the modification workflow and therefore were not included in the validation. The 225 inserted mutations are listed in Table S14 and comprise amino-acid changes (*n* = 172), promoter mutations (*n* = 39), nucleotide mutations (*n* = 9), and premature stop codons (*n* = 5). The negative set was created by modifying sequencing data sets to ensure WT nucleotides at the targeted positions. This resulted in a positive and negative test set of each 11,475 observations (i.e., 225 mutations times 51 samples).

**Pathogen typing.** The spoligotyping assay was evaluated on a positive set of 166 observations and completed with a negative set of 16 observations. The assay was validated at the SIT level with the following definitions: TP and FN, MTBC samples where the SIT detected by the workflow corresponded or did not correspond to the reference SIT, respectively; TN and FP, negative samples where the workflow did not detect or did detect a SIT, respectively.

For SNP-barcoding assay evaluation, the positive and negative sets consisted of 44 and 16 samples, respectively. The following definitions were used: TP and FN, samples from the positive set where the lineage reported by the workflow corresponded or did not correspond to the reference information, respectively; TN, samples from the negative set where sublineage 4.9 was reported (i.e., no SNPs detected at targeted positions, corresponding to the base composition); FP, samples from the negative set where a (sub)lineage other than 4.9 was reported.

For evaluating sequence typing, the cgMLST scheme containing 744 loci was considered. No cgMLST information from conventional methods was available for this assay, and performance was therefore determined by comparing the output from our workflow with the output from the PubMLST sequence query tool (53) (available at https://pubmlst.org/bigsdb?db=pubmlst_mycobacteria_seqdef&page=sequenceQuery). Performance evaluation was done at the locus level only considering perfect hits (i.e., full length and perfect identity), with the following classification definitions: TP and FN, alleles for which workflow output corresponded or did not correspond to reference information, respectively. TN and FP were evaluated by analyzing negative control samples with the workflow, with TN and FP defined as unidentified and identified alleles by our workflow, respectively.

### Data availability.

The data sets supporting the conclusions of this study have been deposited in the NCBI SRA under accession number PRJNA681718 (in-house sequenced data) and Zenodo (http://doi.org/10.5281/zenodo.4434636) (results of all bioinformatics analyses) and are included within this manuscript and its supplemental files (results of the validation).

## RESULTS

### Employed sequencing data.

In total, 238 out of 315 (75.56%) in-house-sequenced samples met the QC thresholds defined in Table 1 and were employed for further validation. Raw and trimmed read counts for the in-house validation samples are provided in Table S15 with a median number of 825,342 read pairs per sample before read trimming. Assembly statistics for the in-house validation samples are also provided in that table and indicated high quality with a median total assembly length of 4,335,544 bp and an *N*_50_ value of 76,947 bp. The H37Rv reference genome mapping rate ranged between 90.28% and 99.31% with a median of 98.39%. An overview of the performance of the different assays is provided in Table 3. Repeatability and reproducibility were always 100% for all assays, since generated processed FASTQ files, assembled contigs, and VCF files were always identical for repeated runs (except for header information). Figure 2 schematically represents the validation workflow for which the results are discussed detail in below; assay-specific definitions are provided in Table 2.

**TABLE 3:** Performance results for the different evaluated bioinformatics assays^*a*^

Group	Assay	No. of TP	No. of FN	No. of TN	No. of FP	Accuracy (%)	Precision (%)	Sensitivity (%)	Specificity (%)	Repeatability (%)	Reproducibility (%)
(Sub)species confirmation and identification	16S rRNA	150	1	64	0	99.53	100	99.34	100	100	100
	*csb/*RD	12/16/13/12^*b*^	0	41/37/40/41^*b*^	0	100	100	100	100	100	100
	*hsp65*	151	0	64	0	100	100	100	100	100	100
SNP-based AMR detection	AMR detection: comparison with molecular methods	118	0	17,776	1	99.99	99.16	100	99.99	100	100
	AMR detection *IS*: positions covered by molecular methods	138	0	137	0	100	100	100	100	100	100
	AMR detection *IS*: positions randomly selected from database	11,371	29	11,456	0	99.87	100	99.75	100	100	100
Pathogen typing	Spoligotyping	148	18	16	0	90.11	100	89.16	100	100	100
	SNP barcoding	44	0	16	0	100	100	100	100	100	100
	Sequence typing (cgMLST)	31,245	3	11,904	0	99.99	100	99.99	100	100	100

a TP, true positive; FP, false positive; TN, true negative; FN false negative; RD, regions of difference; AMR, antimicrobial resistance; IS, *in silico*.

b Validation was performed on a per-species basis. Listed values correspond to M. africanum, M. bovis, M. bovis BCG, and M. tuberculosis (in order).

### (Sub)species identification and confirmation.

For 16S rRNA-based species identification, 150 of 151 observations from the positive set matched the reference information. A single FN was observed in sample SRR6045214 (M. bovis), for which the assembly contained a fracture in the 16S rRNA locus. For the negative test set, no FPs were observed in any of the 64 negative-control samples. This resulted in accuracy, precision, sensitivity, and specificity of 99.53%, 100%, 99.34%, and 100%, respectively. For *hsp65* species identification, all 151 samples from the positive set were correctly identified as MTBC, and no MTBC species were falsely identified in any of the 64 negative-control samples, resulting in accuracy, precision, sensitivity, and specificity of 100%. For the *csb*/RD assay, validation was performed on a per-species basis. All 12, 16, 13, and 12 samples from the positive set were correctly identified for M. africanum, M. bovis, M. bovis BCG, and M. tuberculosis, respectively. No FPs were observed in any of the 41, 37, 40, and 41 negative-control samples for M. africanum, M. bovis, M. bovis BCG, and M. tuberculosis, respectively. This resulted in accuracy, precision, sensitivity, and specificity of 100%.

### SNP-based antimicrobial resistance detection.

Figure 3 presents an overview of the number of observations and included mutations for each mutation type per validation step. In the first validation step, the correspondence of AMR mutations detected with the workflow and molecular methods was evaluated. No FNs were observed in the 118 positions from the positive set (i.e., all mutations were correctly identified). Across 17,777 positions from the negative set, only a single FP was observed in sample S16BD06161, where a C > T mutation at position –15 in the promoter region Rv1482c-*fabG1* not reported by molecular methods was detected. Manual inspection showed that this mutation was present in 100% of reads aligned to this position (see Fig. S4) and was most likely present due to a sample switch, a hypothesis consistent with five discrepancies observed in the spoligotyping data for this sample. This resulted in accuracy, precision, sensitivity, and specificity of 99.99%, 99.16%, 100%, and 99.99%, respectively.

In the second validation step, detection of mutations and wild types inserted *in silico* at positions covered by molecular methods into raw reads of in-house-sequenced data was evaluated. The positive and negative sets were constructed through *in silico* insertion of six mutations into 23 samples, amounting to 138 observations. Sample S08MY01602 contained a preexisting SNP in the targeted codon at a different position than the one reverted to the WT nucleotide that resulted in the insertion of a different amino acid mutation. This position was therefore removed from the negative set of this sample, leading to a final set of 137 observations. For both the positive and negative sets, all mutations and wild types were correctly identified, resulting in accuracy, precision, sensitivity, and specificity of 100%.

In the third validation step, detection of randomly selected mutations from the AMR database inserted *in silico* in in-house-sequenced data sets was evaluated. Out of 11,475 inserted variants for the positive set, 75 were excluded from the validation because they were incorrectly inserted by Bamsurgeon (*n* = 58) or inserted into a codon containing a preexisting mutation affecting the targeted codon (*n* = 17), resulting in a positive test set of 11,400 mutations. Incorrectly inserted variants were traced back to wrong read pairing during an intermediate insertion process step of Bamsurgeon, for which no exact cause could be derived (see the supplemental material). For the negative set, 19 positions were removed because of preexisting mutations in the targeted codon, resulting in a total of 11,456 positions. The workflow missed 29 mutations from the positive set, which were subsequently classified as FN. All missed mutations were amino acid mutations located downstream of stop codons (*n* = 10) and frameshift mutations (*n* = 19), affecting correct detection (see Fig. S5 for example and discussion). All of these mismatches could be explained by the default behavior of BCFtools csq, which is to determine the effect of mutations based on gene coding sequences as a whole, causing it to discard amino acid mutations downstream of a stop codon or indel, as these are not translated. No mutations were falsely identified in the positions covered by the negative test set. This resulted in accuracy, precision, sensitivity, and specificity of 99.87%, 100%, 99.75%, and 100%, respectively.

### Pathogen typing.

For spoligotyping, the positive and negative sets consisted of 166 and 16 samples, respectively. Detailed results are provided in Table S7. The correct spoligotype was detected for 148 samples from the positive set. The 18 mismatches were all caused by detection of spacers that were not detected with the molecular method but were firmly supported by WGS. Seventeen samples had one spacer misidentified, and sample S16BD06161 had five spacers misidentified and was potentially explained by a sample swap (Fig. S4). Spacer 31 accounted for a disproportional number of mismatches in other samples (*n* = 12), but the exact reason could not be determined. No spoligotype was detected by the workflow in any of the 16 negative-control samples, resulting in accuracy, precision, sensitivity, and specificity of 90.11%, 100%, 89.16%, and 100%, respectively. For SNP barcoding, the positive and negative sets consisted of 44 and 16 samples, respectively. The correct lineage was identified for all samples from the positive set, and the reference lineage (lineage 4.9) was identified for all 16 negative data sets, resulting in accuracy, precision, sensitivity, and specificity of 100%. For sequence typing, the positive set consisted of 31,248 observations (i.e., 744 loci times 42 samples selected from the different phylogenetic groups). Only three FNs occurred, which were all mismatches in sample SRR6045301, where the workflow detected multi-hits in the MYCO000483, MYCO000484, and MYCO000486 loci, whereas the reference standard had unique allele calls for these loci. Manual inspection indicated that this was caused by a misassembly resulting in a duplicated sequence of 6,554 bp at the start of a contig, resulting in duplicate occurrences of identical allele sequences (results not shown). The workflow classified these allele calls as multi-hits, while the PubMLST sequence query tool reported them as separate calls for the same allele at different positions in the assembly. The negative set consisted of 16 samples from species outside the *Mycobacterium* genus, resulting in 11,904 observations. No alleles were identified as a perfect hit in any of the negative-control samples by either the workflow or the online pubMLST.org tool. This resulted in accuracy, precision, sensitivity, and specificity of 99.99%, 100%, 99.99%, and 100%, respectively.

## DISCUSSION

**FIG 4:**
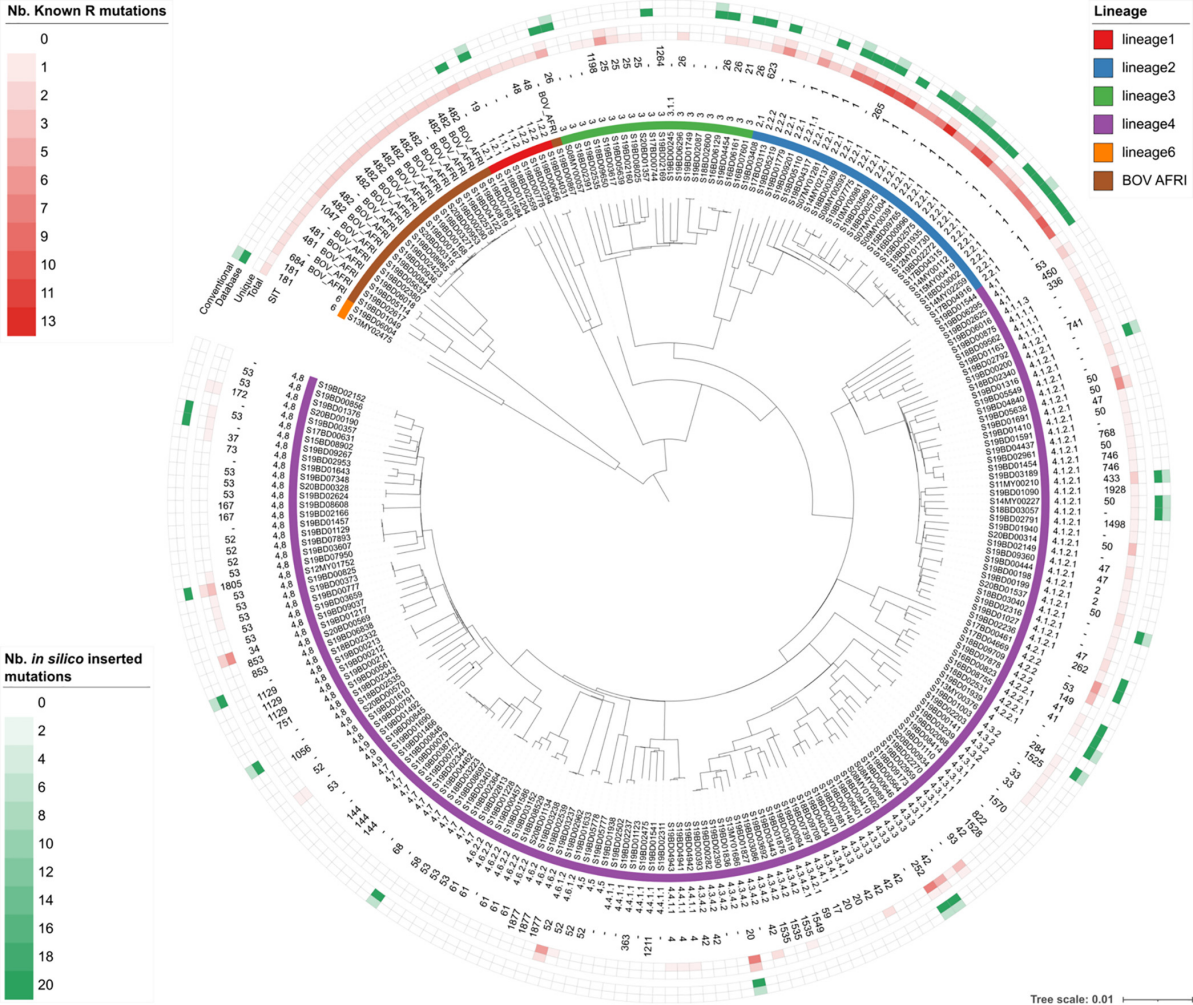
Maximum likelihood tree containing an overview of the diversity of the in-house samples included in the validation data set. The scale bar is expressed as average changes per site. The annotations are (from inner to outer rings) sample name, main lineage determined with the workflow (top-right legend), full lineage detected with the workflow, SIT number determined with conventional spoligotyping, total and unique (i.e., only occurring in the corresponding sample) number of detected mutations with known association(s) with resistance to antibiotics, and number of *in silico* inserted variants selected from the database (“database”) and from the positions covered by the conventional methods (“conventional”). Note that the color scale for the number of *in silico* inserted mutations was capped at 20 to increase clarity; the total number of mutations for all data points in the “database” ring ranged from 217 to 225. Nb, number; SIT, shared international type.

We present an extensive validation of a bioinformatics workflow (Fig. 1) for characterization of MTBC isolates using WGS data generated with Illumina technology. Many clinical and reference laboratories have already switched, or are actively switching, to WGS for their routine activities (4). Correspondingly, the requirement for validation of bioinformatics assays for WGS is increasingly being recognized for its importance for routine implementation in applied settings (35, 67, 68). For M. tuberculosis in particular, Meehan et al. recently highlighted that the lack of standardization and consensus between laboratories complicates the comparability and validation of WGS-based workflows (34). This is especially relevant for MTBC, which typically occurs in a clinical setting. Several validation strategies have been proposed recently (35, 67–69) but are all commonly focused on the correct identification of genes or specific alleles and are thus not directly applicable for MTBC members, for which accurate detection of SNPs is of major importance (10). The performance of SNP-based bioinformatics assays in microbial genomics is typically only performed indirectly, for instance, in phylogenetic studies by the correct placement of delineating strains (70), detection of mutations associated with a particular phenotype (e.g., AMR) (67, 68), or detection of the correct sequence type for MLST (44). Walter et al. recently used a set of 85 SNPs confirmed with Sanger sequencing to evaluate the performance of various variant calling tools for MTBC outbreak investigation (71). Even though individual variants were called with relatively high accuracy, inconsistencies between tools impacted transmission inferences. The differences between the bioinformatics methods illustrate the need for a framework to evaluate the correct detection and identification of individual mutations, which is of particular relevance for antimicrobial resistance prediction in MTBC. Therefore, a validation approach, incorporating and exhaustively characterizing the performance of SNP detection, was proposed (Fig. 2, Table 2). The approach was applied to a diverse data set of 238 in-house-sequenced MTBC isolates characterized extensively with conventional methods. The data set was selected to be representative for the intended application by covering the diversity and lineages that occur in Belgium, but also contained smaller clusters of closely related samples differing sometimes by only a few SNPs (Fig. 4), and consisted of samples observed within the routine activities of the Belgian NRC, complemented with data from public reference collections. However, due to the lack of available data and QC filtering, some lineages were underrepresented in the final validation data set, such as lineages 5 and 7. Nonetheless, our data set can be considered representative for the activities of a reference or clinical laboratory in Belgium (and, by extension, Europe), as these lineages are currently rarely observed by the NRC. If the workflow were employed in a setting where the expected sample composition is substantially different from the validation data set employed here, a revalidation would be required to characterize the performance in the modified setting. For all assays, very high performance was demonstrated, with all performance metrics at >95%, which we postulated as an acceptance criterion before considering an assay as validated. Spoligotyping was, however, a notable exception (see below). In line with previous observations for gene detection and allele calling (35, 36), repeatability and reproducibility were always 100% for all assays, including SNP-based AMR detection, illustrating the stability of bioinformatics analysis across repeated runs within and between computational environments. The validation strategy can be applied to bioinformatics assays for other bacteria, in particular, for SNP-based assays for which currently available validation frameworks are limited.

The (sub)species confirmation and identification component consists of several assays providing partially overlapping information. The 16S rRNA and *hsp65* assays were validated at the MTBC instead of species-level because only 23 of 125 samples contained species-level information, and others were classified as MTBC (*n* = 53) or either M. tuberculosis or M. africanum (*n* = 76). Additionally, the molecular methods themselves on which these WGS assays are based cannot always distinguish species within the *Mycobacterium* genus due to their limited resolution (13). While more powerful WGS-based bioinformatics methods offer much more discriminatory power to assign strains to taxonomic clades, the 16S rRNA, *csb*/RD, and *hsp65* assays were included, as these methods facilitate comparison with historical data. All evaluated (sub)assays showed very high performance, with all performance metrics at >99%, illustrating that WGS is an excellent alternative for these three molecular methods.

The performance of various variant detection workflows has been extensively documented, but typically in the context of human applications such as detection of mutations with clinical significance in oncology and rare diseases (72, 73), and are consequently not directly applicable to bacterial pathogens. A main bottleneck in evaluating variant detection workflows is the lack of sufficient reference information at a SNP-level resolution, which is often impossible or infeasible to obtain. Therefore, *in silico*-generated or -modified data sets are often used to complement validation efforts for human applications on top of actual reference data generated in the lab (74). To obtain representative results, simulated data must represent real data as closely as possible. Read simulation software can only approximate sequencing error profiles, which can vary between and within sequencing centers (75). Methods that insert variants by modifying reads of real sequencing data sets do not suffer from this limitation (64). Whenever possible, real sequencing data from well-characterized reference materials should be used in conjunction with *in silico* methods. Therefore, we evaluated variant detection with both reference information from molecular methods and simulated data using a three-step approach. First, a reference set of AMR-associated positions was characterized in our validation data set of in-house-sequenced samples by using molecular methods, creating a high-quality “real” reference data set to compare WGS results against, demonstrating very high performance, with all metrics at >99%. This approach is similar to a previous study where mutations confirmed with Sanger sequencing were used to characterize the performance of AMR detection in M. tuberculosis. Schleusener et al. (26) reported similar performance on a limited set of mutations with concordance varying between ∼98 and 100% depending on the targeted gene. Second, a subset of positions included in the first step was subjected to *in silico* modification by inserting either the WT or mutation in raw sequencing reads of in-house-sequenced data. We found again very high performance with perfect detection in all modified data sets. This approach served as justification for extending our validation data set with *in silico*-modified data covering many more positions of the total AMR database than would have been possible using molecular approaches. Third, an *in silico* approach was taken to modify WGS data sets at positions randomly selected from the entire database with AMR-associated mutations, also demonstrating very high performance, with all performance metrics at >99%, demonstrating the feasibility of using SNP-based AMR detection for WGS data in a clinical and/or national reference lab settings. However, our approach has some limitations. First, indels were not considered here due to their complexity. Second, only the detection of consensus mutations was evaluated. Information on hetero-resistance is provided informatively and should not be considered validated, as accurate detection requires a median sequencing coverage much higher than the minimum required by the pipeline, especially for mutations present at low allelic frequencies, rendering this still prohibitively expensive for routine activities (Table 1). The validation of SNP-based AMR detection was performed exclusively at the genotypic level, since bioinformatics workflows should be evaluated at this level (i.e., correct or incorrect detection of a variant), whereas evaluation of genotype-phenotype correspondence is at a higher level (76). An explorative comparison outside the scope of the validation was nevertheless performed on the subset of the validation data set with phenotypic testing data available, resulting in a correspondence of 96.08% between the predicted phenotype based on detected SNPs and observed AMR phenotypes, indicating similarly high performance (Table S16).

For pathogen typing, three assays were validated, spoligotyping, SNP barcoding, and cgMLST. Phylogenetic classification at the SNP level allows the highest possible resolution and is expected to outperform conventional methods for (sub)species identification and lineage detection. However, the spoligotyping, SNP barcoding, and cgMLST assays still provide pertinent information for routine pathogen typing. Due to their use throughout the years, a wealth of historical data is available, allowing the placement of isolates in the context of historical background collections. Additionally, since MTB outbreaks typically span multiple years, overlap with conventional methods is essential to monitor ongoing outbreaks (77). In contrast to all other assays, performance for spoligotyping was substantially lower, with accuracy and sensitivity dropping to 90.11% and 89.16%, respectively, far below our postulated acceptance criterion of 95%. Conventional spoligotyping can provide ambiguous results with spacers that can be classified as either present or absent, and *in silico* spoligotyping is notoriously difficult with short-read data, as these spacers are located in highly repetitive genomic regions (78). The observed performance is in line with benchmarks of commonly used tools such as SpolPred (30) and SpoTyping (29). Nevertheless, since spoligotyping constitutes a relevant assay for clinicians due to the large volume of historical data, the assay was retained in the workflow at specific request, albeit as an indicative assay not meeting our *a priori* 95% acceptance criterion. The SNP barcoding and cgMLST assays did both demonstrate very high performance, with all performance metrics at >99%. Compared to spoligotyping, both methods were designed for WGS data and offer higher resolution by specifically considering a set of 413 SNPs and 744 loci located over the entire chromosome, respectively. Their usability is, however, hindered by lacking historical data, which will require substantial sequencing efforts. Nonetheless, the relatively low performance of spoligotyping compared to SNP barcoding and cgMLST highlights the urgent need for a paradigm shift by migrating toward WGS-based approaches for pathogen typing and the need to start building “new” historical data sets comprising full genomic information (19, 79). Although WGS-based *in silico* MIRU-VNTR typing was not included in our workflow, similar to spoligotyping, it has been reported to suffer from lower accuracy when only short-read data are available (80) and would therefore equally benefit from being phased out in favor of the more performant WGS-based typing methods. Neither cgMLST nor SNP barcoding offer enough resolution to detect closely related outbreaks characterized by only a few SNPs difference (81). Detecting such outbreaks requires employing variant detection workflows in conjunction with advanced phylogenomics tree reconstruction methods. Although a SNP-based phylogenetic tree was presented for describing variation in the validation data set (Fig.4), SNP-based phylogenetic inference itself was therefore not validated, because it would require validating methods dependent on the exact relationship between individual samples. Nonetheless, the high performance for variant detection of SNP-based AMR demonstrates that SNP calling can occur with high performance, suggesting that phylogenomics methods using SNPs, as presented in Fig. 4, can also achieve very high performance. Nevertheless, an important advantage of cgMLST and SNP barcoding is that both methods offer a harmonized and standardized framework for which all samples can be compared, whereas resolving closely related outbreaks using phylogenomics methods based on variant detection is heavily context-dependent.

Our bioinformatics workflow is provided through a Galaxy interface as a “push-button” implementation for nonexpert bioinformaticians and is available for nonprofit use at https://galaxy.sciensano.be. Such solutions are particularly relevant for laboratories that lack sufficient bioinformatics expertise or specialized hardware, such as low-income countries where tuberculosis is often a major problem. The workflow is compatible with data generated on all Illumina platforms. The minimum read length check is determined dynamically based on the input read length, making it compatible with data sets with reads shorter than the 2 × 250-bp reads generated by the Illumina MiSeq device on the in-house-generated samples. This was illustrated by the validation data set, which also contained data from public sources generated using the Illumina HiSeq and Illumina Genome Analyzer II. We stress that several viable alternative Web-based and command-line solutions exist for *Mycobacterium* pathogen typing (see introductory section), which can be equally or even better suited for specific target audiences. Our main consideration is that a more rigid framework for demonstrating minimal performance of WGS-based methods is urgently required, especially for clinical laboratories working under a quality system, for which labs can employ whatever bioinformatics method they prefer, whether in-house-developed, commercial, or Web-based. Such a strategy focusing on performance-based evaluation rather than enforcing strict methods is recommended, allowing flexibility in employed solutions and accounting for the large diversity and quick evolution in the many WGS technologies that exist (82). This is also in line with recommendations of ongoing initiatives such as ISO (83) and also reflects the approach taken, for instance, by laboratories processing human patient genomic data for clinical purposes such as oncology (84). Such a framework is also relevant for other microbial pathogens and will aid in the standardization and integration of WGS in routine clinical and/or public health contexts.
