# Supplementary material for: A Bioinformatics Whole-Genome Sequencing Workflow for Clinical Mycobacterium tuberculosis Complex Isolate Analysis, Validated Using a Reference Collection Extensively Characterized with Conventional Methods and In Silico Approaches
Source: J Clin Microbiol. 2021 May 19;59(6):e00202-21. doi: 10.1128/JCM.00202-21 (PMC8316078; doi:10.1128/JCM.00202-21)
Supplement: Supplemental file 1 — Supplemental methods, Tables S1 to S16, and Fig. S1 to S4. Download jcm.00202-21-s0001.pdf, PDF file, 2.9 MB [file jcm.00202-21-s0001.pdf]

SUPPLEMENTARY

A bioinformatics WGS workflow for clinical *Mycobacterium tuberculosis* complex isolate analysis, validated using a reference collection extensively characterized with conventional methods and *in silico* approaches

Methods

Evaluation of causes of mis-insertion of variants into sequencing data used to validate the detection of antimicrobial resistance.

Output log files from the bamsurgeon software used to insert variants were checked for error messages. 10 cases were associated with the error message "[fread] Unexpected end of file" and 48 with the error message "[bwa_sai2sam_pe_core] paired reads have different names", both of which can be traced back to read mis-pairing. The exact reason for read-mis-pairing could not be established but the initial fastq files used to insert variants were checked for consistency of read pairing between forward and reverse fastq files by comparing the positions of reads named in the bamsurgeon log files using the sed 4.4 (https://www.gnu.org/software/sed/manual/sed.html) and wc (GNU coreutils 8.28, http://www.gnu.org/software/coreutils/) unix tools.

Tables

Table S1: Regions targeted for AMR prediction.

This table lists the regions that are targeted for genotypic AMR prediction. The genomic coordinates in the H37Rv reference genome are listed in the first two columns. The third and fourth columns list the locus and the type, respectively. The fifth and sixth columns list the antibiotic (s) and the type of resistance interaction, respectively.

Start	End	Locus	Type	Antibiotic	Resistance / compensatory
5240	7267	gyrB	protein_coding	Fluoroquinolone	resistance
7302	9818	gyrA	protein_coding	Fluoroquinolone	resistance
575348	576790	mshA	protein_coding	Isoniazid	resistance
759310	759806	Rv0666-rpoB	intergenic	Rifampicin	resistance
759807	763325	rpoB	protein_coding	Rifampicin	resistance
763370	767320	rpoC	protein_coding	Rifampicin	compensatory
775586	778480	mmpL5	protein_coding	Bedaquiline	resistance
778477	778905	mmpS5	protein_coding	Bedaquiline	resistance
778990	779487	Rv0678	protein_coding	Bedaquiline	resistance
781560	781934	rpsL	protein_coding	Streptomycin	resistance
800809	801462	rplC	protein_coding	Linezolid	resistance
1461045	1461290	atpE	protein_coding	Bedaquiline	resistance
1471846	1473382	rrs	rRNA	Amikacin, Capreomycin, Kanamycin, Streptomycin	resistance
1473658	1476795	rrl	rRNA	Linezolid	resistance
1673300	1673439	Rv1482c-fabG1	intergenic	Isoniazid, Para-aminosalicylic acid	resistance
1673440	1674183	fabG1	protein_coding	Isoniazid	resistance
1674184	1674201	inhA-fabG1	intergenic	Isoniazid, Ethionamide and prothionamide	resistance
1674202	1675011	inhA	protein_coding	Isoniazid	resistance
1833542	1834987	rpsA	protein_coding	Pyrazinamide	resistance
1917940	1918746	tlyA	protein_coding	Capreomycin	resistance
1929786	1931456	cycA	protein_coding	D-cycloserine	resistance
2101651	2103042	ndh	protein_coding	Isoniazid	resistance
2153889	2156111	katG	protein_coding	Isoniazid	resistance
2156112	2156148	katG-furA	intergenic	Isoniazid	resistance
2156149	2156592	furA	protein_coding	Isoniazid	resistance
2156593	2156705	Rv1910c-furA	intergenic	Isoniazid	resistance
2288681	2289241	pncA	protein_coding	Pyrazinamide	resistance
2289242	2289281	pncA-Rv2044c	intergenic	Pyrazinamide	resistance
2715333	2715471	eis-Rv2417c	intergenic	Kanamycin	resistance
2725571	2726087	oxyR	pseudogene	Isoniazid	resistance
2726088	2726192	oxyR'-aphC	intergenic	Isoniazid	resistance
2726193	2726780	ahpC	protein_coding	Isoniazid	resistance
2746135	2747598	folC	protein_coding	Para-aminosalicylic acid	resistance
2859300	2860418	Rv2535c	protein_coding	Bedaquiline, Clofazimine	resistance
3004745	3005650	Rv2688c	protein_coding	Fluoroquinolone	resistance

3067946	3068460	thyX-hsdM	intergenic	Para-aminosalicylic acid	resistance
3073130	3073609	dfrA	protein_coding	Para-aminosalicylic acid	resistance
3073680	3074471	thyA	protein_coding	Para-aminosalicylic acid	resistance
3127364	3128272	Rv2820c	protein_coding	Ethambutol	resistance
3272214	3273209	drrA	protein_coding	Multidrug efflux	resistance
3511318	3511681	PPE52-nuoA	intergenic	Para-aminosalicylic acid	resistance
3568401	3568679	whiB7	protein_coding	Kanamycin, Amikacin	resistance
3628160	3629647	sahH	protein_coding	Para-aminosalicylic acid	resistance
3633584	3633675	Rv3253c-Rv3254c	intergenic	Para-aminosalicylic acid	resistance
3685983	3686900	Rv3300c	protein_coding	Ethambutol	resistance
3840194	3841420	alr	protein_coding	D-cycloserine	resistance
3841421	3841714	alr-Rv3424c	intergenic	D-cycloserine	resistance
3877464	3878507	rpoA	protein_coding	Rifampicin	compensatory
3986844	3987299	ddn	protein_coding	Delamanid	resistance
4043862	4044281	panD	protein_coding	Pyrazinamide	resistance
4239863	4243147	embC	protein_coding	Ethambutol	resistance
4243148	4243232	embC-embA	intergenic	Ethambutol	resistance
4243233	4246517	embA	protein_coding	Ethambutol	resistance
4246514	4249810	embB	protein_coding	Ethambutol	resistance
4268925	4269833	ubiA	protein_coding	Ethambutol	resistance
4326004	4327473	ethA	protein_coding	Ethionamide and prothionamide	resistance
4327549	4328199	ethR	protein_coding	Ethionamide and prothionamide	resistance
4407528	4408202	gid	protein_coding	Streptomycin	resistance

Table S2: Summary of all samples used for the validation of the bioinformatics workflow.

The first column contains sample names, the following columns respectively display the assays that each sample was used to perform the validation of, identified by "X". Samples marked with an "*" refer to samples which were modified *in silico* and should be read as sample name + "_full_snps_AMR_sanger" and sample name + "_ref_AMR_sanger" for samples used in the IS-sanger validation of the AMR module (see supplementary table S10) or as sample name + "_full_ref_AMR_3_nostops", sample name + "_full_snps_AMR_3_nostops" and sample name + "_full_snps_AMR_3_stops" for samples used in the IS-random validation of the AMR module (see Supplementary table S11). *In silico* modified datasets were not uploaded to SRA, accession numbers are therefore not available ('NA') for these samples.

**Sample**	**SRA accession**	***hsp65* / 16S**	***csb* / RD**	**Spoligotyping**	**SNP barcoding**	**Typing**	**AMR: molecular**	**AMR: *IS* molecular**	**AMR: *IS* database**
NC_CJ00001	ERR690668	X		X	X	X			
NC_CJ00002	ERR690765	X		X	X	X			
NC_CJ00003	ERR690787	X		X	X	X			
NC_CJ00004	ERR694502	X		X	X	X			
NC_CJ00005	ERR694575	X		X	X	X			
NC_NM00001	SRR6953924	X		X	X	X			
NC_NM00002	SRR6953903	X		X	X	X			
NC_NM00003	SRR6953901	X		X	X	X			
NC_NM00004	SRR6954084	X		X	X	X			
NC_NM00005	SRR6954008	X		X	X	X			
NC_SE00001	SRR5055288	X		X	X	X			
NC_SE00002	SRR5583186	X		X	X	X			
NC_SE00003	SRR5815674	X		X	X	X			
NC_SE00004	SRR5850014	X		X	X	X			
NC_SE00005	SRR5864444	X		X	X	X			
NC_SE00006	SRR6131972	X		X	X	X			
S07MY01004	SRR13180435	X		X		X			
S07MY01281	SRR13180434	X		X					
S08MY00057	SRR13180266	X		X		X			
S08MY00593	SRR13180218	X		X					
S08MY00891	SRR13180207	X		X					

S08MY01602	SRR13180361	X		X					
S09MY00391	SRR13180350	X		X			X		
S10MY00981	SRR13180339	X		X			X		
S11MY00210	SRR13180328	X		X					
S12MY01730	SRR13180281	X		X			X		
S12MY01752	SRR13180433	X		X			X		
S13MY00376	SRR13180422	X		X		X			
S13MY01686	SRR13180411	X		X					
S13MY02475	SRR13180400	X		X		X			
S14MY00112	SRR13180389	X		X			X		
S14MY00227	SRR13180378	X		X					
S14MY02137	SRR13180367	X		X			X		
S14MY02259	SRR13180320	X		X			X		
S15BD02575	SRR13180309	X	X	X			X		
S15BD05110	SRR13180298	X	X	X			X		
S15BD08902	SRR13180265	X					X		
S15BD09765	SRR13180254	X	X	X					
S15MY00419	SRR13180243	X		X					
S16BD00823	SRR13180232	X	X	X		X	X		
S16BD00996	SRR13180224	X		X					
S16BD06129	SRR13180223	X	X	X			X		
S16BD06161	SRR13180222	X	X	X			X		
S16BD07601	SRR13180221	X	X	X			X		
S16BD08755	SRR13180220	X	X	X			X		
S17BD00461	SRR13180219	X	X	X		X	X		
S17BD00631	SRR13180217	X	X	X			X		
S17BD00744	SRR13180216	X	X				X		
S17BD01584	SRR13180215	X	X				X		
S17BD03113	SRR13180214	X					X		
S17BD04315	SRR13180213	X					X		
S17BD04669	SRR13180212	X					X		
S17BD04916	SRR13180211	X		X			X		
S18BD00369	SRR13180210	X					X		
S18BD00575	SRR13180209	X					X		
S18BD01935	SRR13180208	X					X		
S18BD02332	SRR13180206	X							
S18BD02340	SRR13180205	X							
S18BD02391	SRR13180204	X							

S18BD02531	SRR13180203	X							
S18BD02535	SRR13180202	X							
S18BD02559	SRR13180201	X				X			
S18BD02600	SRR13180200	X							
S18BD03002	SRR13180199	X					X		
S18BD03040	SRR13180363	X							
S18BD03057	SRR13180362	X							
S18BD03223	SRR13180360	X							
S18BD09410	SRR13180358	X					X		
S18BD09708	SRR13180355	X		X					
S18BD09709	SRR13180354	X		X			X		
S19BD00079	SRR13180353	X		X					
S19BD00094	SRR13180352	X		X		X			
S19BD00140	SRR13180351	X		X					
S19BD00141	SRR13180349	X		X			X		
S19BD00167	SRR13180348	X	X	X			X		
S19BD00168	SRR13180347	X	X	X			X		
S19BD00198	SRR13180346	X		X					
S19BD00199	SRR13180345	X		X					
S19BD00200	SRR13180344	X							
S19BD00211	SRR13180343	X		X					
S19BD00212	SRR13180342	X		X					
S19BD00213	SRR13180341	X		X					
S19BD00282	SRR13180340	X		X					
S19BD00290	SRR13180338	X	X	X			X		
S19BD00357	SRR13180337	X							
S19BD00373	SRR13180336	X		X					
S19BD00393	SRR13180335	X		X					
S19BD00444	SRR13180334	X		X					
S19BD00457	SRR13180333	X		X					
S19BD00561	SRR13180332	X		X					
S19BD00564	SRR13180331	X					X		
S19BD00646	SRR13180330	X					X		
S19BD00752	SRR13180291	X							
S19BD00777	SRR13180290	X		X					
S19BD00778	SRR13180289	X				X			
S19BD00791	SRR13180288	X		X					
S19BD00819	SRR13180287	X	X	X			X		

S19BD00825	SRR13180286	X		X					
S19BD00844	SRR13180285	X	X	X			X		
S19BD00845	SRR13180284	X		X		X			
S19BD00846	SRR13180283	X		X					
S19BD00856	SRR13180282	X		X					
S19BD00867	SRR13180280	X					X		
S19BD00936	SRR13180278	X	X	X			X		
S19BD00970	SRR13180277	X		X					
S19BD01003	SRR13180276	X		X			X		
S19BD01027	SRR13180275	X							
S19BD01049	SRR13180274	X	X	X			X		
S19BD01090	SRR13180273	X		X					
S19BD01123	SRR13180271	X		X					
S19BD01129	SRR13180432	X		X					
S19BD01163	SRR13180431	X							
S19BD01204	SRR13180430	X		X		X			
S19BD01217	SRR13180429	X		X			X		
S19BD01228	SRR13180428	X		X					
S19BD01316	SRR13180427	X					X		
S19BD01376	SRR13180426	X		X					
S19BD01410	SRR13180425	X		X					
S19BD01454	SRR13180424	X		X					
S19BD01457	SRR13180423	X							
S19BD01466	SRR13180421	X				X			
S19BD01492	SRR13180420	X					X		
S19BD01541	SRR13180419	X		X		X			
S19BD01544	SRR13180418	X							
S19BD01586	SRR13180417	X							
S19BD01591	SRR13180416	X							
S19BD01610	SRR13180415	X							
S19BD01633	SRR13180414	X		X					
S19BD01643	SRR13180413	X							
S19BD01690	SRR13180412	X							
S19BD01691	SRR13180410	X		X					
S19BD01749	SRR13180409	X							
S19BD01779	SRR13180408	X				X			
S19BD01827	SRR13180407	X							
S19BD01836	SRR13180406	X							

S19BD01876	SRR13180405	X		X			X		
S19BD01938	SRR13180404	X							
S19BD01939	SRR13180403	X							
S19BD01940	SRR13180402	X							
S19BD02068	SRR13180401	X							
S19BD02087	SRR13180399	X							
S19BD02149	SRR13180398	X							
S19BD02152	SRR13180397	X							
S19BD02160	SRR13180396	X		X			X		
S19BD02203	SRR13180393	X					X		
S19BD02236	SRR13180392	X		X			X		
S19BD02237	SRR13180391	X							
S19BD02270	SRR13180390	X		X					
S19BD02272	SRR13180388	X		X			X		
S19BD02311	SRR13180387	X							
S19BD02316	SRR13180386	X							
S19BD02343	SRR13180385	X				X			
S19BD02344	SRR13180384	X							
S19BD02380	SRR13180382	X	X				X		
S19BD02390	SRR13180381	X							
S19BD02394	SRR13180380	X							
S19BD02423	SRR13180379	X	X				X		
S19BD02475	SRR13180377	X							
S19BD02502	SRR13180376	X							
S19BD02535	SRR13180375	X							
S19BD02579	SRR13180373	X	X				X		
S19BD02617	SRR13180372	X	X				X		
SRR6044788	SRR6044788	X							
SRR6044792	SRR6044792	X							
SRR6044839	SRR6044839	X							
SRR6044854	SRR6044854	X							
SRR6044858	SRR6044858	X	X						
SRR6044920	SRR6044920	X							
SRR6044922	SRR6044922	X							
SRR6044965	SRR6044965	X							
SRR6045054	SRR6045054	X							
SRR6045061	SRR6045061	X							
SRR6045062	SRR6045062	X							

SRR6045079	SRR6045079	X							
SRR6045087	SRR6045087	X							
SRR6045122	SRR6045122	X							
SRR6045214	SRR6045214	X							
SRR6045223	SRR6045223	X							
SRR6045274	SRR6045274	X							
SRR6045412	SRR6045412	X							
SRR6045436	SRR6045436	X							
SRR6045444	SRR6045444	X							
SRR6045477	SRR6045477	X							
SRR6045528	SRR6045528	X							
SRR6045554	SRR6045554	X							
SRR6045580	SRR6045580	X							
SRR6045611	SRR6045611	X							
SRR6045648	SRR6045648	X							
SRR6045672	SRR6045672	X							
SRR6045732	SRR6045732	X							
SRR6045765	SRR6045765	X							
SRR6045809	SRR6045809	X							
SRR6045827	SRR6045827	X							
SRR6045830	SRR6045830	X							
SRR6045996	SRR6045996	X							
SRR6045998	SRR6045998	X							
SRR6046049	SRR6046049	X							
SRR6046080	SRR6046080	X							
SRR6046100	SRR6046100	X							
SRR6046120	SRR6046120	X							
SRR6046199	SRR6046199	X							
SRR6046252	SRR6046252	X							
SRR6046334	SRR6046334	X	X						
SRR6046444	SRR6046444	X							
SRR6046511	SRR6046511	X							
SRR6046543	SRR6046543	X							
SRR6046549	SRR6046549	X							
SRR6046635	SRR6046635	X							
SRR6046651	SRR6046651	X							
SRR6046698	SRR6046698	X							
SRR6046732	SRR6046732	X							

SRR6046744	SRR6046744	X							
SRR6046773	SRR6046773	X							
SRR6046801	SRR6046801	X							
SRR6046832	SRR6046832	X							
ERR025455	ERR025455				X				
ERR025842	ERR025842				X				
ERR027453	ERR027453				X				
ERR028608	ERR028608				X				
ERR028617	ERR028617				X				
ERR028627	ERR028627				X				
ERR036187	ERR036187				X				
ERR037533	ERR037533				X				
ERR038253	ERR038253				X				
ERR038277	ERR038277				X	X			
ERR038749	ERR038749				X	X			
ERR039338	ERR039338				X				
ERR040119	ERR040119				X				
ERR046838	ERR046838				X				
ERR046897	ERR046897				X				
ERR046933	ERR046933				X	X			
ERR046937	ERR046937				X				
ERR046941	ERR046941				X	X			
ERR046954	ERR046954				X	X			
ERR047001	ERR047001				X	X			
ERR047004	ERR047004				X				
ERR047880	ERR047880				X				
ERR047890	ERR047890				X				
ERR072039	ERR072039				X				
ERR1035199	ERR1035199				X				
ERR234111	ERR234111				X				
ERR234164	ERR234164				X				
ERR234173	ERR234173				X				
ERR234180	ERR234180				X				
ERR234181	ERR234181				X				
ERR234190	ERR234190				X				
ERR234216	ERR234216				X				
ERR234251	ERR234251				X				
ERR234257	ERR234257				X				

ERR234268	ERR234268				X				
ERR234272	ERR234272				X				
ERR245674	ERR245674				X	X			
ERR245684	ERR245684				X	X			
ERR245749	ERR245749				X				
ERR245762	ERR245762				X				
S18BD08529	SRR13180359			X					
S18BD09562	SRR13180357					X			
S18BD09650	SRR13180356					X			
S19BD00245	SRR13259086					X			
S19BD00656	SRR13180329			X					
S19BD00875	SRR13180279					X			
S19BD01110	SRR13180272					X			
S19BD02166	SRR13180395			X					
S19BD02169	SRR13180394			X					
S19BD02364	SRR13180383			X					
S19BD02539	SRR13180374			X					
S19BD02624	SRR13180371			X					
S19BD02625	SRR13180370			X					
S19BD02791	SRR13180369			X					
S19BD02792	SRR13180368			X					
S19BD02813	SRR13180366			X					
S19BD02953	SRR13180365			X					
S19BD02959	SRR13180364			X					
S19BD02961	SRR13180327			X					
S19BD02962	SRR13180326			X					
S19BD03086	SRR13180325			X					
S19BD03152	SRR13180324			X		X			
S19BD03189	SRR13180323			X					
S19BD03237	SRR13180322			X					
S19BD03238	SRR13180321			X		X			
S19BD03239	SRR13180319			X					
S19BD03271	SRR13180318			X					
S19BD03401	SRR13180317			X					
S19BD03408	SRR13180316			X		X			
S19BD03443	SRR13180315			X					
S19BD03569	SRR13180314			X					
S19BD03607	SRR13180313			X					

S19BD03617	SRR13180312			X					
S19BD03619	SRR13180311			X					
S19BD03659	SRR13180310			X					
S19BD03692	SRR13180308			X					
S19BD03871	SRR13180307			X					
S19BD04031	SRR13180306			X		X			
S19BD04034	SRR13180305			X					
S19BD04122	SRR13180304			X					
S19BD04317	SRR13180303			X					
S19BD04437	SRR13180302			X					
S19BD04454	SRR13180301			X		X			
S19BD04462	SRR13180300			X					
S19BD04840	SRR13180299			X					
S19BD04941	SRR13180297			X					
S19BD04942	SRR13180296			X					
S19BD04943	SRR13180295			X					
S19BD05114	SRR13180294			X					
S19BD05219	SRR13180293			X					
S19BD05549	SRR13180292					X			
S19BD05637	SRR13180270			X					
S19BD05638	SRR13180269			X					
S19BD05639	SRR13180268			X					
S19BD05777	SRR13180267			X					
S19BD05778	SRR13180264			X		X			
S19BD06004	SRR13180263			X		X			
S19BD06016	SRR13180262			X					
S19BD06018	SRR13180261			X					
S19BD06295	SRR13180260			X		X			
S19BD06296	SRR13180259			X					
S19BD06838	SRR13180258			X					
S19BD07348	SRR13180257			X					
S19BD07397	SRR13180256			X					
S19BD07681	SRR13180255			X					
S19BD07775	SRR13180253			X					
S19BD07878	SRR13180252			X					
S19BD07881	SRR13180251			X					
S19BD07893	SRR13180250			X					
S19BD07950	SRR13180249			X					

S19BD08025	SRR13180248			X					
S19BD08414	SRR13180247			X					
S19BD08608	SRR13180246			X					
S19BD08697	SRR13180245			X					
S19BD08985	SRR13180244			X					
S19BD09037	SRR13180242			X					
S19BD09173	SRR13180241			X					
S19BD09201	SRR13180240			X		X			
S19BD09267	SRR13180239			X					
S19BD09360	SRR13180238			X					
S19BD09501	SRR13180237			X					
S20BD00190	SRR13180236			X					
S20BD00314	SRR13180235			X					
S20BD00315	SRR13180234			X					
S20BD00328	SRR13180233			X					
S20BD00569	SRR13180231			X					
S20BD00570	SRR13180230			X					
S20BD00934	SRR13180229			X					
S20BD00953	SRR13180228			X					
S20BD01134	SRR13180227			X					
S20BD01357	SRR13180226			X					
S20BD01537	SRR13180225			X					
SRR1915476	SRR1915476		X						
SRR1915477	SRR1915477		X			X			
SRR1915478	SRR1915478		X						
SRR1915488	SRR1915488		X						
SRR1915489	SRR1915489		X						
SRR5486073	SRR5486073		X			X			
SRR5486074	SRR5486074		X						
SRR5486075	SRR5486075		X						
SRR5486076	SRR5486076		X						
SRR5486077	SRR5486077		X						
SRR5486078	SRR5486078		X						
SRR5486079	SRR5486079		X						
SRR5486080	SRR5486080		X						
SRR5486081	SRR5486081		X						
SRR5486082	SRR5486082		X						
SRR5486083	SRR5486083		X						

SRR5486084	SRR5486084		X						
SRR5486085	SRR5486085		X						
SRR5486086	SRR5486086		X						
SRR6044939	SRR6044939		X						
SRR6045015	SRR6045015		X						
SRR6045301	SRR6045301		X			X			
SRR6045425	SRR6045425		X						
SRR6045962	SRR6045962		X						
SRR6046131	SRR6046131		X						
SRR6046235	SRR6046235		X						
SRR6046675	SRR6046675		X			X			
SRR6046695	SRR6046695		X						
SRR6046725	SRR6046725		X						
SRR671762	SRR671762				X	X			
SRR671797	SRR671797				X	X			
SRR671816	SRR671816				X				
SRR671872	SRR671872				X				
S07MY01004 *	NA							X	X
S07MY01281 *	NA							X	X
S08MY00057 *	NA								X
S08MY00593 *	NA							X	X
S08MY00891 *	NA							X	X
S08MY01602 *	NA							X	X
S09MY00391 *	NA								X
S10MY00981 *	NA								X
S11MY00210 *	NA							X	X
S12MY01730 *	NA								X
S12MY01752 *	NA								X
S13MY00376 *	NA							X	X
S13MY01686 *	NA							X	X
S13MY02475 *	NA							X	X
S14MY00112 *	NA								X
S14MY00227 *	NA							X	X
S14MY02137 *	NA								X
S14MY02259 *	NA								X
S15BD02575 *	NA								X
S15BD05110 *	NA								X
S15BD08902 *	NA								X

S15BD09765 *	NA							X	X
S15MY00419 *	NA							X	X
S16BD00823 *	NA								X
S16BD00996 *	NA							X	X
S16BD06129 *	NA								X
S16BD06161 *	NA								X
S16BD07601 *	NA								X
S16BD08755 *	NA								X
S17BD00461 *	NA								X
S17BD00631 *	NA								X
S17BD00744 *	NA								X
S17BD01584 *	NA								X
S17BD03113 *	NA								X
S17BD04315 *	NA								X
S17BD04669 *	NA								X
S17BD04916 *	NA								X
S18BD00369 *	NA								X
S18BD00575 *	NA								X
S18BD01935 *	NA								X
S18BD02332 *	NA							X	X
S18BD02340 *	NA							X	X
S18BD02391 *	NA							X	X
S18BD02531 *	NA							X	X
S18BD02535 *	NA							X	X
S18BD02559 *	NA							X	X
S18BD02600 *	NA							X	X
S18BD03002 *	NA								X
S18BD03040 *	NA							X	X
S18BD03057 *	NA							X	X
S18BD03223 *	NA							X	X

Table S3: Samples used for the validation of the 16S and *hsp65* assays.

The first columns lists the sample name. The second column lists species (or species complex) and the thirds column lists the class of the sample for the validation of the assays.

Sample name	species	Sample class
S07MY01004	MTBC	Positive
S07MY01281	MTBC	Positive
S08MY00057	MTBC	Positive
S08MY00593	MTBC	Positive
S08MY00891	MTBC	Positive
S08MY01602	MTBC	Positive
S09MY00391	MTBC	Positive
S10MY00981	MTBC	Positive
S11MY00210	MTBC	Positive
S12MY01730	MTBC	Positive
S12MY01752	MTBC	Positive
S13MY00376	MTBC	Positive
S13MY01686	MTBC	Positive
S13MY02475	*Mycobacterium africanum*	Positive
S14MY00112	MTBC	Positive
S14MY00227	MTBC	Positive
S14MY02137	MTBC	Positive
S14MY02259	MTBC	Positive
S15BD02575	MTBC	Positive
S15BD05110	MTBC	Positive
S15BD08902	MTBC	Positive
S15BD09765	MTBC	Positive
S15MY00419	MTBC	Positive
S16BD00823	MTBC	Positive
S16BD00996	MTBC	Positive
S16BD06129	MTBC	Positive
S16BD06161	MTBC	Positive
S16BD07601	MTBC	Positive
S16BD08755	MTBC	Positive
S17BD00461	MTBC	Positive
S17BD00631	MTBC	Positive
S17BD00744	MTBC	Positive
S17BD01584	*Mycobacterium tuberculosis*	Positive
S17BD03113	MTBC	Positive
S17BD04315	MTBC	Positive
S17BD04669	MTBC	Positive
S17BD04916	MTBC	Positive
S18BD00369	MTBC	Positive
S18BD00575	MTBC	Positive
S18BD01935	MTBC	Positive
S18BD02332	*Mycobacterium tuberculosis*	Positive
S18BD02340	MTBC	Positive
S18BD02391	MTBC	Positive
S18BD02531	MTBC	Positive
S18BD02535	*Mycobacterium tuberculosis*	Positive
S18BD02559	MTBC	Positive
S18BD02600	*Mycobacterium tuberculosis*	Positive
S18BD03002	MTBC	Positive
S18BD03040	*Mycobacterium tuberculosis*	Positive
S18BD03057	MTBC	Positive

S18BD03223	MTBC	Positive
S18BD09410	MTBC	Positive
S18BD09708	*Mycobacterium tuberculosis/africanum*	Positive
S18BD09709	*Mycobacterium tuberculosis/africanum*	Positive
S19BD00079	*Mycobacterium tuberculosis/africanum*	Positive
S19BD00094	*Mycobacterium tuberculosis/africanum*	Positive
S19BD00140	*Mycobacterium tuberculosis/africanum*	Positive
S19BD00141	*Mycobacterium tuberculosis/africanum*	Positive
S19BD00167	*Mycobacterium bovis BCG*	Positive
S19BD00168	*Mycobacterium bovis BCG*	Positive
S19BD00198	*Mycobacterium tuberculosis/africanum*	Positive
S19BD00199	*Mycobacterium tuberculosis/africanum*	Positive
S19BD00200	*Mycobacterium tuberculosis/africanum*	Positive
S19BD00211	*Mycobacterium tuberculosis/africanum*	Positive
S19BD00212	*Mycobacterium tuberculosis/africanum*	Positive
S19BD00213	*Mycobacterium tuberculosis/africanum*	Positive
S19BD00282	*Mycobacterium tuberculosis/africanum*	Positive
S19BD00290	*Mycobacterium bovis BCG*	Positive
S19BD00357	*Mycobacterium tuberculosis/africanum*	Positive
S19BD00373	*Mycobacterium tuberculosis/africanum*	Positive
S19BD00393	*Mycobacterium tuberculosis/africanum*	Positive
S19BD00444	*Mycobacterium tuberculosis/africanum*	Positive
S19BD00457	*Mycobacterium tuberculosis/africanum*	Positive
S19BD00561	*Mycobacterium tuberculosis/africanum*	Positive
S19BD00564	*Mycobacterium tuberculosis/africanum*	Positive
S19BD00646	*Mycobacterium tuberculosis/africanum*	Positive
S19BD00752	*Mycobacterium tuberculosis/africanum*	Positive
S19BD00777	*Mycobacterium tuberculosis/africanum*	Positive
S19BD00778	MTBC	Positive
S19BD00791	*Mycobacterium tuberculosis/africanum*	Positive
S19BD00819	*Mycobacterium bovis BCG*	Positive
S19BD00825	*Mycobacterium tuberculosis/africanum*	Positive
S19BD00844	*Mycobacterium bovis BCG*	Positive
S19BD00845	*Mycobacterium tuberculosis/africanum*	Positive
S19BD00846	*Mycobacterium tuberculosis/africanum*	Positive
S19BD00856	*Mycobacterium tuberculosis/africanum*	Positive
S19BD00867	*Mycobacterium bovis*	Positive
S19BD00936	*Mycobacterium bovis BCG*	Positive
S19BD00970	*Mycobacterium tuberculosis/africanum*	Positive
S19BD01003	*Mycobacterium tuberculosis/africanum*	Positive
S19BD01027	*Mycobacterium tuberculosis/africanum*	Positive
S19BD01049	*Mycobacterium bovis*	Positive
S19BD01090	*Mycobacterium tuberculosis/africanum*	Positive
S19BD01123	MTBC	Positive
S19BD01129	*Mycobacterium tuberculosis/africanum*	Positive
S19BD01163	*Mycobacterium tuberculosis/africanum*	Positive
S19BD01204	*Mycobacterium tuberculosis/africanum*	Positive
S19BD01217	*Mycobacterium tuberculosis/africanum*	Positive
S19BD01228	*Mycobacterium tuberculosis/africanum*	Positive
S19BD01316	*Mycobacterium tuberculosis/africanum*	Positive
S19BD01376	*Mycobacterium tuberculosis/africanum*	Positive
S19BD01410	*Mycobacterium tuberculosis/africanum*	Positive
S19BD01454	MTBC	Positive
S19BD01457	*Mycobacterium tuberculosis/africanum*	Positive
S19BD01466	*Mycobacterium tuberculosis/africanum*	Positive
S19BD01492	*Mycobacterium tuberculosis/africanum*	Positive
S19BD01541	*Mycobacterium tuberculosis/africanum*	Positive
S19BD01544	*Mycobacterium tuberculosis/africanum*	Positive

S19BD01586	*Mycobacterium tuberculosis/africanum*	Positive
S19BD01591	MTBC	Positive
S19BD01610	*Mycobacterium tuberculosis/africanum*	Positive
S19BD01633	*Mycobacterium tuberculosis/africanum*	Positive
S19BD01643	MTBC	Positive
S19BD01690	*Mycobacterium tuberculosis/africanum*	Positive
S19BD01691	*Mycobacterium tuberculosis/africanum*	Positive
S19BD01749	MTBC	Positive
S19BD01779	*Mycobacterium tuberculosis/africanum*	Positive
S19BD01827	*Mycobacterium tuberculosis/africanum*	Positive
S19BD01836	*Mycobacterium tuberculosis/africanum*	Positive
S19BD01876	*Mycobacterium tuberculosis/africanum*	Positive
S19BD01938	*Mycobacterium tuberculosis/africanum*	Positive
S19BD01939	*Mycobacterium tuberculosis/africanum*	Positive
S19BD01940	*Mycobacterium tuberculosis/africanum*	Positive
S19BD02068	*Mycobacterium tuberculosis/africanum*	Positive
S19BD02087	*Mycobacterium tuberculosis/africanum*	Positive
S19BD02149	*Mycobacterium tuberculosis/africanum*	Positive
S19BD02152	*Mycobacterium tuberculosis/africanum*	Positive
S19BD02160	*Mycobacterium tuberculosis/africanum*	Positive
S19BD02203	*Mycobacterium tuberculosis/africanum*	Positive
S19BD02236	*Mycobacterium tuberculosis/africanum*	Positive
S19BD02237	*Mycobacterium tuberculosis/africanum*	Positive
S19BD02270	*Mycobacterium tuberculosis/africanum*	Positive
S19BD02272	*Mycobacterium tuberculosis/africanum*	Positive
S19BD02311	*Mycobacterium tuberculosis/africanum*	Positive
S19BD02316	MTBC	Positive
S19BD02343	*Mycobacterium tuberculosis/africanum*	Positive
S19BD02344	*Mycobacterium tuberculosis/africanum*	Positive
S19BD02380	*Mycobacterium bovis*	Positive
S19BD02390	*Mycobacterium tuberculosis/africanum*	Positive
S19BD02394	*Mycobacterium tuberculosis/africanum*	Positive
S19BD02423	*Mycobacterium bovis BCG*	Positive
S19BD02475	*Mycobacterium tuberculosis/africanum*	Positive
S19BD02502	*Mycobacterium tuberculosis/africanum*	Positive
S19BD02535	*Mycobacterium tuberculosis/africanum*	Positive
S19BD02579	*Mycobacterium bovis BCG*	Positive
S19BD02617	*Mycobacterium bovis BCG*	Positive
SRR6044858	*Mycobacterium africanum*	Positive
SRR6045214	*Mycobacterium bovis*	Positive
SRR6045827	*Mycobacterium bovis*	Positive
SRR6046252	*Mycobacterium bovis*	Positive
SRR6046334	*Mycobacterium africanum*	Positive
NC_CJ00001	*Campylobacter jejuni*	Negative
NC_CJ00002	*Campylobacter jejuni*	Negative
NC_CJ00003	*Campylobacter jejuni*	Negative
NC_CJ00004	*Campylobacter jejuni*	Negative
NC_CJ00005	*Campylobacter jejuni*	Negative
NC_NM00001	*Neisseria meningitidis*	Negative
NC_NM00002	*Neisseria meningitidis*	Negative
NC_NM00003	*Neisseria meningitidis*	Negative
NC_NM00004	*Neisseria meningitidis*	Negative
NC_NM00005	*Neisseria meningitidis*	Negative
NC_SE00001	*Salmonella enteritidis*	Negative
NC_SE00002	*Salmonella enteritidis*	Negative
NC_SE00003	*Salmonella enteritidis*	Negative
NC_SE00004	*Salmonella enteritidis*	Negative
NC_SE00005	*Salmonella enteritidis*	Negative

NC_SE00006	*Salmonella enteritidis*	Negative
SRR6044788	*Mycobacterium sp. 'ratisbonense'*	Negative
SRR6044792	*Mycobacterium gordonae*	Negative
SRR6044839	*Mycobacterium paraffinicum*	Negative
SRR6044854	*Mycobacterium marinum*	Negative
SRR6044920	*Mycobacterium intracellulare*	Negative
SRR6044922	*Mycobacterium colombiense*	Negative
SRR6044965	*Mycobacterium arosiense*	Negative
SRR6045054	*Mycobacterium szulgai*	Negative
SRR6045061	*Mycobacterium malmoense*	Negative
SRR6045062	*Mycobacterium marseillense*	Negative
SRR6045079	*Mycobacterium marseillense*	Negative
SRR6045087	*Mycobacterium szulgai*	Negative
SRR6045122	*Mycobacterium kansasii*	Negative
SRR6045223	*Mycobacterium avium*	Negative
SRR6045274	*Mycobacterium xenopi*	Negative
SRR6045412	*Mycobacterium shimoidei*	Negative
SRR6045436	*Mycobacterium arosiense*	Negative
SRR6045444	*Mycobacterium lentiflavum*	Negative
SRR6045477	*Mycobacterium simiae*	Negative
SRR6045528	*Mycobacterium malmoense*	Negative
SRR6045554	*Mycobacterium kansasii*	Negative
SRR6045580	*Mycobacterium angelicum*	Negative
SRR6045611	*Mycobacterium szulgai*	Negative
SRR6045648	*Mycobacterium xenopi*	Negative
SRR6045672	*Mycobacterium lentiflavum*	Negative
SRR6045732	*Mycobacterium kansasii*	Negative
SRR6045765	*Mycobacterium paraffinicum*	Negative
SRR6045809	*Mycobacterium palustre*	Negative
SRR6045830	*Mycobacterium avium*	Negative
SRR6045996	*Mycobacterium triplex*	Negative
SRR6045998	*Mycobacterium malmoense*	Negative
SRR6046049	*Mycobacterium chimaera*	Negative
SRR6046080	*Mycobacterium marinum*	Negative
SRR6046100	*Mycobacterium sp. 'ratisbonense'*	Negative
SRR6046120	*Mycobacterium chimaera*	Negative
SRR6046199	*Mycobacterium paraffinicum*	Negative
SRR6046444	*Mycobacterium intracellulare*	Negative
SRR6046511	*Mycobacterium marseillense*	Negative
SRR6046543	*Mycobacterium gordonae*	Negative
SRR6046549	*Mycobacterium lentiflavum*	Negative
SRR6046635	*Mycobacterium gordonae*	Negative
SRR6046651	*Mycobacterium chimaera*	Negative
SRR6046698	*Mycobacterium sp. 'ratisbonense'*	Negative
SRR6046732	*Mycobacterium xenopi*	Negative
SRR6046744	*Mycobacterium triplex*	Negative
SRR6046773	*Mycobacterium shimoidei*	Negative
SRR6046801	*Mycobacterium colombiense*	Negative
SRR6046832	*Mycobacterium avium*	Negative

Table S4: Phenotypic AMR susceptibility testing.

	First Line		Second line (group A)		Second line (group B)		Second line (group C)	Second line (group D)	Other					
	Isoniazid	Rifampicin	Ofloxacin	Moxifloxacin	Amikacin	Capreomycin	Ethionamide	Pyrazinamide	Ethambutol	Rifabutine	PAS	Linezolid	Clofazimine	Bedaquiline
S17BD04916	R	R	S	S	R	R	R	R	R	R	S	S	S	S
S18BD09709	R	R	S	S	S	S	S	R	R	R	S	S	R	S
S19BD00168	S	S	-	-	-	-	-	R	S	-	-	-	-	-
S19BD04122	S	S	-	-	-	-	-	R	S	-	-	-	-	-
S19BD04840	R	S	S	S	S	S	R	R	R	S	S	S	S	S
S19BD05638	R	S	S	S	S	S	R	R	R	S	S	S	S	S
S19BD07681	S	S	-	-	-	-	-	R	S	-	-	-	-	-
S19BD07775	R	R	S	S	S	S	R	R	R	R	S	S	S	S
S19BD00819	S	S	-	-	-	-	-	R	S	-	-	-	-	-
S19BD00844	S	S	-	-	-	-	-	R	S	-	-	-	-	-
S19BD09201	R	R	S	S	S	S	R	R	S	R	S	S	R	S
S19BD00936	S	S	-	-	-	-	-	R	S	-	-	-	-	-
S19BD01217	R	R	S	S	S	S	R	R	S	R	S	S	S	S
S19BD01492	S	S	S	S	S	S	S	R	S	S	S	S	R	S
S19BD02394	S	S	-	-	-	-	-	R	S	-	-	-	-	-
S19BD02423	S	S	-	-	-	-	-	R	S	-	-	-	-	-
S19BD02579	S	S	-	-	-	-	-	R	S	-	-	-	-	-
S18BD09410	S	S	-	-	-	-	-	S	S	-	-	-	-	-
S19BD00079	S	S	-	-	-	-	-	S	S	-	-	-	-	-
S19BD00094	S	S	-	-	-	-	-	S	S	-	-	-	-	-
S19BD00140	S	S	-	-	-	-	-	S	S	-	-	-	-	-
S19BD00141	S	S	-	-	-	-	-	S	S	-	-	-	-	-
S19BD00200	S	S	S	S	-	-	-	S	S	-	-	-	-	-
S19BD00282	S	S	-	-	-	-	-	S	S	-	-	-	-	-
S19BD03569	R	R	-	R	S	S	R	S	S	R	S	S	S	S
S19BD00357	S	S	-	-	-	-	-	S	S	-	-	-	-	-
S19BD03692	S	S	-	-	-	-	-	S	S	-	-	-	-	-
S19BD00373	S	S	-	-	-	-	-	S	S	-	-	-	-	-
S19BD00393	S	S	-	-	-	-	-	S	S	-	-	-	-	-
S19BD00444	S	S	-	-	-	-	-	S	S	-	-	-	-	-
S19BD04454	R	S	-	-	-	-	-	S	S	-	-	-	-	-
S19BD00457	S	S	-	-	-	-	-	S	S	-	-	-	-	-
S19BD05549	S	S	-	-	-	-	-	S	S	-	-	-	-	-
S19BD00561	S	S	-	-	-	-	-	S	S	-	-	-	-	-
S19BD05639	R	R	S	S	S	S	S	S	S	R	R	S	S	S
S19BD06004	R	S	S	S	S	S	-	S	S	S	S	S	S	S
S19BD06295	R	S	S	S	S	S	R	S	S	S	S	S	S	S
S19BD06296	R	S	S	S	S	S	S	S	S	S	S	S	S	S
S19BD00646	R	S	-	-	-	-	-	S	S	-	-	-	-	-
S19BD00752	S	S	-	-	-	-	-	S	S	-	-	-	-	-
S19BD00777	S	S	-	-	-	-	-	S	S	-	-	-	-	-
S19BD07881	R	R	S	S	S	S	S	S	S	R	S	S	S	S
S19BD07950	R	S	S	S	S	S	R	S	S	S	S	S	R	S
S19BD00825	S	S	-	-	-	-	-	S	S	-	-	-	-	-
S19BD00845	S	S	-	-	-	-	-	S	S	-	-	-	-	-
S19BD00856	S	S	-	-	-	-	-	S	S	-	-	-	-	-
S19BD09173	-	-	-	-	-	-	-	S	-	-	-	-	-	-
S19BD01003	S	S	-	-	-	-	-	S	S	-	-	-	-	-
S19BD01027	S	S	-	-	-	-	-	S	S	-	-	-	-	-

S19BD01090	S	S	-	-	-	-	-	S	S	-	-	-	-	-
S19BD01123	S	S	-	-	-	-	-	S	S	-	-	-	-	-
S19BD01129	S	S	-	-	-	-	-	S	S	-	-	-	-	-
S19BD01163	S	S	-	-	-	-	-	S	S	-	-	-	-	-
S19BD01204	S	S	-	-	-	-	-	S	S	-	-	-	-	-
S19BD01316	R	S	-	-	-	-	-	S	S	-	-	-	-	-
S19BD01376	-	-	-	-	-	-	-	S	-	-	-	-	-	-
S19BD01410	S	S	-	-	-	-	-	S	S	-	-	-	-	-
S19BD01457	S	S	-	-	-	-	-	S	S	-	-	-	-	-
S19BD01466	S	S	-	-	-	-	-	S	S	-	-	-	-	-
S19BD01544	S	S	-	-	-	-	-	S	S	-	-	-	-	-
S19BD01586	S	S	-	-	-	-	-	S	S	-	-	-	-	-
S19BD01610	S	S	-	-	-	-	-	S	S	-	-	-	-	-
S19BD01633	S	S	-	-	-	-	-	S	S	-	-	-	-	-
S19BD01643	S	S	-	-	-	-	-	S	S	-	-	-	-	-
S19BD01749	S	S	-	-	-	-	-	S	S	-	-	-	-	-
S19BD01779	S	S	-	-	-	-	-	S	S	-	-	-	-	-
S19BD01836	S	S	-	-	-	-	-	S	S	-	-	-	-	-
S19BD01876	R	S	-	-	-	-	-	S	S	-	-	-	-	-
S19BD01938	S	S	-	-	-	-	-	S	S	-	-	-	-	-
S19BD01939	S	S	-	-	-	-	-	S	S	-	-	-	-	-
S19BD02087	S	S	-	-	-	-	-	S	S	-	-	-	-	-
S19BD02149	S	S	-	-	-	-	-	S	S	-	-	-	-	-
S19BD02152	S	S	-	-	-	-	-	S	S	-	-	-	-	-
S19BD02160	S	R	S	S	S	S	S	S	S	R	S	S	S	S
S19BD02203	S	S	-	-	-	-	-	S	S	-	-	-	-	-
S19BD02272	R	R	S	S	S	S	S	S	S	R	R	S	R	S
S19BD02311	S	S	-	-	-	-	-	S	S	-	-	-	-	-
S19BD02343	S	S	-	-	-	-	-	S	S	-	-	-	-	-
S19BD02344	S	S	-	-	-	-	-	S	S	-	-	-	-	-
S19BD02390	S	S	-	-	-	-	-	S	S	-	-	-	-	-
S19BD02475	S	S	-	-	-	-	-	S	S	-	-	-	-	-
S19BD02535	S	S	-	-	-	-	-	S	S	-	-	-	-	-
S19BD00846	S	-	S	S	S	S	S	-	-	S	S	S	S	S
S19BD02068	-	-	S	S	-	-	-	-	-	-	-	-	-	-
S19BD02236	R	-	S	S	-	-	-	-	-	-	-	-	-	-
S19BD02237	-	-	S	S	-	-	-	-	-	-	-	-	-	-

Results of phenotypical AMR susceptibility testing. ‘R’ and ‘S’ indicate that the sample was resistant or susceptible to the corresponding antibiotic, respectively. Dashes (‘-‘) indicate combinations that were not tested.

Table S5: Results of AMR screening with PCR amplification and LPAs.

The first column displays sample names, the second and third column contain the mutations characterized by PCR specifically designed to target mutation S315T in *katG* and C-15T in *inhA*, the fourth, fifth and sixth columns contain the mutations characterized by the genotyping assay MTBDR+ in genes *rpoB* (codons 505, 508, 509, 510, 511, 513, 516, 515, 518, 522, 526, 531, 531 and 533), *katG* (codon 315) and *inhA* (promoter nucleotides -8, -15 and -16), whereas columns seven to eleven contain mutations evidenced by the GenoType LPA kits targeting genes *gyrA* (codons 88, 90, 91 and 94), *rrs* (nucleotides A1401, C1402 and G1484), *embB* (codon 306), *gyrB* (codons 538 and 540) and *eis* (promoter nucleotides C-2, G-10, C-12, C-14 and G-37). The eight column displays mutations evidenced in the *rpoB* gene by sanger sequencing (targeting codons 475-591). "/" represent samples for which the specific assay has not been performed. "*wt*" represent samples for which no mutation was identified in any of the positions targeted by the specific assay.

Sample	PCR		Genotype kit MTBDR+			Genotype kit MTBRsl+				
	katG	***inhA***	***rpoB***	***katG***	***inhA***	***gyrA***	***rrs***	***embB***	***gyrB***	***eis***
S17BD03113	/	/	/	S315T	*wt*	*wt*	A1401G	/	*wt*	*wt*
S17BD04315	/	/	S531L	S315T	*wt*	/	/	/	/	/
S17BD04669	/	/	S531L	S315T	*wt*	*wt*	*wt*	/	*wt*	*wt*
S18BD00369	S315T	C-15T	/	/	/	*wt*	*wt*	/	*wt*	*wt*
S18BD00575	/	/	/	S315T	*wt*	*wt*	*wt*	/	*wt*	*wt*
S18BD01935	/	/	S531L	S315T	/	/	/	/	/	/
S18BD03002	/	/	S531L	S315T	*wt*	/	/	/	/	/
S09MY00391	/	/	/	/	/	D94G	A1401G	M306V	/	/
S10MY00981	/	/	/	/	/	D94G	A1401G	M306V	/	/
S12MY01730	/	/	S531L	S315T	*wt*	/	/	/	/	/
S12MY01752	/	/	S531L	S315T	*wt*	/	/	/	/	/
S14MY00112	/	/	S531L	S315T	*wt*	D94G	wt	M306V	/	/
S14MY02137	/	/	S531L	S315T	C-15T	/	/	/	/	/
S14MY02259	/	/	S531L	S315T	*wt*	/	/	/	/	/
S15BD02575	/	/	/	S315T	*wt*	D94G	A1401G	/	/	/
S15BD05110	/	C-15T	/	/	/	/	/	/	/	/
S15BD08902	/	C-15T	/	/	/	/	/	/	/	/
S16BD00823	/	C-15T	/	/	/	/	/	/	/	/
S16BD06129	/	C-15T	*wt*	*wt*	C-15T	/	/	/	/	/
S16BD06161	/	/	*wt*	*wt*	*wt*	/	/	/	/	/
S16BD07601	/	C-15T	/	/	/	/	/	/	/	/
S16BD08755	/	C-15T	/	/	/	/	/	/	/	/
S17BD00461	/	/	*wt*	S315T	*wt*	/	/	/	/	/
S17BD00631	/	/	*wt*	*wt*	C-15T	/	/	/	/	/
S17BD00744	/	/	*wt*	*wt*	*wt*	/	/	/	/	/
S17BD01584	/	C-15T	*wt*	*wt*	C-15T	/	/	/	/	/

Table S6: Results of AMR screening with Sanger sequencing.

Mutations identified in specific genes for each sample, based on sanger sequencing. All mutations are at the amino-acid level, except for those indicated with parentheses that correspond to nucleotide mutations, e.g. C(-15)T. "/" denotes a sample for which no information was obtained for a specific gene. "*wt*" describes samples for which no mutation was identified in a specific gene.

**Sample**	***katG***	***inhA***	***mabA***	***ethA***	***rpoB***	***embB***	***pncA***	***gyrA***	***rpsL***	***rrs***	***tlyA***	***thyA***
S17BD04916	R463L, S315T	*wt*	*wt*	*wt*	S450L	*wt*	K96T	S95T	K43R	A(1401)G	*wt*	*wt*
S19BD01217	S315T	*wt*	C(-15)T	*wt*	S450L	*wt*	A146V	*wt*	*wt*	*wt*	*wt*	/
S19BD00564	/	*wt*	*wt*	*wt*	S450L	M306I	D12N	S95T	*wt*	*wt*	/	/
S19BD02272	/	*wt*	*wt*	*wt*	S450L	*wt*	*wt*	S95T	K43R	*wt*	/	/
S19BD02203	*wt*	*wt*	/	*wt*	*wt*	*wt*	*wt*	S95T	*wt*	C(492)T	*wt*	T202A
S19BD01003	*wt*	*wt*	*wt*	*wt*	*wt*	*wt*	H43Y	S95T	*wt*	C(492)T	/	T202A
S19BD00141	*wt*	/	*wt*	*wt*	*wt*	*wt*	*wt*	S95T	*wt*	C(492)T	*wt*	/
S19BD01316	*wt*	*wt*	C(-15)T	*wt*	*wt*	*wt*	*wt*	S95T	*wt*	*wt*	*wt*	/
S18BD09709	S315T	*wt*	*wt*	*wt*	S450L	M306I	A(-11)G	S95T	*wt*	*wt*	*wt*	*wt*
S19BD00167	/	*wt*	*wt*	*wt*	*wt*	*wt*	H57D	S95T	*wt*	*wt*	/	/
S19BD00168	/	*wt*	*wt*	*wt*	*wt*	*wt*	H57D	S95T	/	*wt*	*wt*	/
S19BD00290	R463L	*wt*	*wt*	*wt*	*wt*	*wt*	H57D	S95T	*wt*	*wt*	*wt*	wt
S19BD00646	S315T	*wt*	*wt*	*wt*	*wt*	*wt*	*wt*	S95T	*wt*	*wt*	*wt*	T202A
S19BD00819	R463L	*wt*	*wt*	*wt*	*wt*	*wt*	H57D	S95T	*wt*	/	*wt*	*wt*
S19BD00844	/	*wt*	*wt*	*wt*	*wt*	D280N	H57D	S95T	*wt*	*wt*	*wt*	/
S19BD00867	R463L	*wt*	*wt*	*wt*	*wt*	*wt*	/	S95T	*wt*	*wt*	*wt*	*wt*
S19BD00936	R463L	*wt*	*wt*	*wt*	*wt*	D280N	H57D	S95T	*wt*	*wt*	*wt*	/
S19BD01049	R463L	*wt*	*wt*	*wt*	*wt*	*wt*	H57D	S95T	*wt*	*wt*	*wt*	*wt*
S19BD01492	*wt*	*wt*	/	*wt*	D436Y	*wt*	*wt*	/	*wt*	*wt*	/	*wt*
S19BD01876	/	/	*wt*	/	/	/	/	*wt*	/	/	/	/
S19BD02160	R463L	*wt*	*wt*	*wt*	H445Y	*wt*	*wt*	S95T	*wt*	*wt*	*wt*	/
S19BD02236	*wt*	*wt*	*wt*	*wt*	*wt*	*wt*	*wt*	S95T	*wt*	*wt*	*wt*	/
S19BD02380	R463L	*wt*	*wt*	*wt*	*wt*	*wt*	H57D	S95T	*wt*	*wt*	*wt*	/

S19BD02423	/	*wt*	*wt*	*wt*	*wt*	*wt*	H57D	S95T	*wt*	*wt*	/	/
S19BD02579	R463L	*wt*	*wt*	*wt*	*wt*	*wt*	H57D	S95T	*wt*	*wt*	/	/
S19BD02617	R463L	*wt*	*wt*	*wt*	*wt*	*wt*	H57D	S95T	*wt*	*wt*	*wt*	/
S17BD03113	/	/	/	/	H445R	/	/	/	/	/	/	/
S18BD09410	*wt*	*wt*	*wt*	*wt*	*wt*	*wt*	*wt*	S95T	*wt*	*wt*	*wt*	T202A
S18BD09562	*wt*	*wt*	*wt*	*wt*	*wt*	*wt*	*wt*	S95T	*wt*	*wt*	*wt*	/
S18BD09650	R463L	*wt*	*wt*	*wt*	*wt*	*wt*	*wt*	S95T	*wt*	*wt*	*wt*	*wt*

Table S7: Samples used for the validation of the spoligotyping module and results generated by the workflow

The first and second and third columns contain the names and species of samples, respectively. Species for samples with ambiguous typing are separated by "/" (*Mycobacterium tuberculosis/africanum* and *Mycobacterium bovis/bovis BCG*). The third column contains the class to which the sample was attributed for validation (Positive or Negative). Fourth and fifth columns contain respectively the SIT obtained by molecular methods and SIT reported by the workflow (mismatches indicated in bold). "/" refers to samples with no reported SIT. The sixth column contains the spacer sequences that were not reported by the workflow in positive samples (in bold), or "/" if no spacer was missed.

Sample name	Species	Sample class	SIT	Workflow SIT	Missed spacers
S07MY01004	MTBC	Positive	1	1	/
S07MY01281	MTBC	Positive	1	1	/
S08MY00057	MTBC	Positive	26	26	/
S08MY00593	MTBC	Positive	**265**	**1**	**37**
S08MY00891	MTBC	Positive	42	42	/
S08MY01602	MTBC	Positive	252	252	/
S09MY00391	MTBC	Positive	1	1	/
S10MY00981	MTBC	Positive	1	1	/
S11MY00210	MTBC	Positive	**433**	**/**	**31**
S12MY01730	MTBC	Positive	1	1	/
S12MY01752	MTBC	Positive	1805	1805	/
S13MY00376	MTBC	Positive	284	284	/
S13MY01686	MTBC	Positive	20	20	/
S13MY02475	*Mycobacterium africanum*	Positive	181	181	/
S14MY00112	MTBC	Positive	1	1	/
S14MY00227	MTBC	Positive	**50**	**53**	**31**
S14MY02137	MTBC	Positive	1	1	/
S14MY02259	MTBC	Positive	1	1	/
S15BD02575	*Mycobacterium tuberculosis/africanum*	Positive	1	1	/
S15MY00419	MTBC	Positive	1	1	/
S15BD05110	*Mycobacterium tuberculosis/africanum*	Positive	1	1	/
S15BD09765	*Mycobacterium tuberculosis/africanum*	Positive	1	1	/
S16BD00823	*Mycobacterium tuberculosis/africanum*	Positive	41	41	/
S16BD00996	MTBC	Positive	1	1	/
S16BD06129	*Mycobacterium tuberculosis/africanum*	Positive	26	26	/
S16BD06161	*Mycobacterium tuberculosis/africanum*	Positive	**21**	**26**	**10, 20, 21, 22, 35**

S16BD07601	*Mycobacterium tuberculosis/africanum*	Positive	26	26	/
S16BD08755	*Mycobacterium tuberculosis/africanum*	Positive	41	41	/
S17BD00461	*Mycobacterium tuberculosis/africanum*	Positive	262	262	/
S17BD00631	*Mycobacterium tuberculosis/africanum*	Positive	53	53	/
S17BD04916	*Mycobacterium tuberculosis/africanum*	Positive	1	1	/
S18BD08529	*Mycobacterium tuberculosis/africanum*	Positive	61	61	/
S18BD09708	*Mycobacterium tuberculosis/africanum*	Positive	20	20	/
S18BD09709	*Mycobacterium tuberculosis/africanum*	Positive	53	53	/
S19BD00079	*Mycobacterium tuberculosis/africanum*	Positive	144	144	/
S19BD00094	*Mycobacterium tuberculosis/africanum*	Positive	59	59	/
S19BD00140	*Mycobacterium tuberculosis/africanum*	Positive	42	42	/
S19BD00141	*Mycobacterium tuberculosis/africanum*	Positive	33	33	/
S19BD00167	*Mycobacterium bovis/BCG*	Positive	482	482	/
S19BD00168	*Mycobacterium bovis/BCG*	Positive	482	482	/
S19BD00198	*Mycobacterium tuberculosis/africanum*	Positive	2	2	/
S19BD00199	*Mycobacterium tuberculosis/africanum*	Positive	2	2	/
S19BD00211	*Mycobacterium tuberculosis/africanum*	Positive	1129	1129	/
S19BD00212	*Mycobacterium tuberculosis/africanum*	Positive	1129	1129	/
S19BD00213	*Mycobacterium tuberculosis/africanum*	Positive	1129	1129	/
S19BD00282	*Mycobacterium tuberculosis/africanum*	Positive	42	42	/
S19BD00290	*Mycobacterium bovis BCG*	Positive	482	482	/
S19BD00373	*Mycobacterium tuberculosis/africanum*	Positive	53	53	/
S19BD00393	*Mycobacterium tuberculosis/africanum*	Positive	42	42	/
S19BD00444	*Mycobacterium tuberculosis/africanum*	Positive	47	47	/
S19BD00457	*Mycobacterium tuberculosis/africanum*	Positive	61	61	/
S19BD00561	*Mycobacterium tuberculosis/africanum*	Positive	751	751	/
S19BD00656	*Mycobacterium tuberculosis/africanum*	Positive	48	48	/

S19BD00777	*Mycobacterium tuberculosis/africanum*	Positive	53	53	/
S19BD00791	*Mycobacterium tuberculosis/africanum*	Positive	52	52	/
S19BD00819	*Mycobacterium bovis BCG*	Positive	482	482	/
S19BD00825	*Mycobacterium tuberculosis/africanum*	Positive	53	53	/
S19BD00844	*Mycobacterium bovis BCG*	Positive	482	482	/
S19BD00845	*Mycobacterium tuberculosis/africanum*	Positive	53	53	/
S19BD00846	*Mycobacterium tuberculosis/africanum*	Positive	144	144	/
S19BD00856	*Mycobacterium tuberculosis/africanum*	Positive	53	53	/
S19BD00936	*Mycobacterium bovis BCG*	Positive	482	482	/
S19BD00970	*Mycobacterium tuberculosis/africanum*	Positive	42	42	/
S19BD01003	*Mycobacterium tuberculosis/africanum*	Positive	1525	1525	/
S19BD01049	*Mycobacterium bovis*	Positive	684	684	/
S19BD01090	*Mycobacterium tuberculosis/africanum*	Positive	1928	1928	/
S19BD01123	MTBC	Positive	363	363	/
S19BD01129	*Mycobacterium tuberculosis/africanum*	Positive	52	52	/
S19BD01204	*Mycobacterium tuberculosis/africanum*	Positive	19	19	/
S19BD01217	*Mycobacterium tuberculosis/africanum*	Positive	34	34	/
S19BD01228	*Mycobacterium tuberculosis/africanum*	Positive	61	61	/
S19BD01376	*Mycobacterium tuberculosis/africanum*	Positive	53	53	/
S19BD01410	*Mycobacterium tuberculosis/africanum*	Positive	**50**	**53**	**31**
S19BD01454	MTBC	Positive	**746**	**462**	**31**
S19BD01541	*Mycobacterium tuberculosis/africanum*	Positive	1211	1211	/
S19BD01633	*Mycobacterium tuberculosis/africanum*	Positive	52	52	/
S19BD01691	*Mycobacterium tuberculosis/africanum*	Positive	47	47	/
S19BD01876	*Mycobacterium tuberculosis/africanum*	Positive	1535	1535	/
S19BD02160	*Mycobacterium tuberculosis/africanum*	Positive	25	25	/
S19BD02166	*Mycobacterium tuberculosis/africanum*	Positive	167	167	/
S19BD02169	*Mycobacterium tuberculosis/africanum*	Positive	1264	1264	/

S19BD02236	*Mycobacterium tuberculosis/africanum*	Positive	47	47	/
S19BD02270	*Mycobacterium tuberculosis/africanum*	Positive	1528	1528	/
S19BD02272	*Mycobacterium tuberculosis/africanum*	Positive	1	1	/
S19BD02364	*Mycobacterium tuberculosis/africanum*	Positive	53	53	/
S19BD02539	*Mycobacterium tuberculosis/africanum*	Positive	1877	1166	4
S19BD02624	*Mycobacterium tuberculosis/africanum*	Positive	53	53	/
S19BD02625	*Mycobacterium tuberculosis/africanum*	Positive	450	450	/
S19BD02791	*Mycobacterium tuberculosis/africanum*	Positive	1498	1498	/
S19BD02792	*Mycobacterium tuberculosis/africanum*	Positive	741	44	31
S19BD02813	*Mycobacterium tuberculosis/africanum*	Positive	53	53	/
S19BD02953	*Mycobacterium tuberculosis/africanum*	Positive	73	73	/
S19BD02959	*Mycobacterium tuberculosis/africanum*	Positive	42	42	/
S19BD02961	*Mycobacterium tuberculosis/africanum*	Positive	50	53	31
S19BD02962	*Mycobacterium tuberculosis/africanum*	Positive	52	52	/
S19BD03086	*Mycobacterium tuberculosis/africanum*	Positive	42	42	/
S19BD03152	*Mycobacterium tuberculosis/africanum*	Positive	61	61	/
S19BD03189	*Mycobacterium tuberculosis/africanum*	Positive	**746**	**462**	**31**
S19BD03237	*Mycobacterium tuberculosis/africanum*	Positive	**1877**	**1166**	**4**
S19BD03238	*Mycobacterium tuberculosis/africanum*	Positive	**1877**	**1166**	**4**
S19BD03239	*Mycobacterium tuberculosis/africanum*	Positive	33	33	/
S19BD03271	*Mycobacterium bovis/BCG*	Positive	482	482	/
S19BD03401	*Mycobacterium tuberculosis/africanum*	Positive	53	53	/
S19BD03408	*Mycobacterium tuberculosis/africanum*	Positive	623	623	/
S19BD03443	*Mycobacterium tuberculosis/africanum*	Positive	1535	1535	/
S19BD03569	*Mycobacterium tuberculosis/africanum*	Positive	1	1	/
S19BD03607	*Mycobacterium tuberculosis/africanum*	Positive	52	52	/
S19BD03617	*Mycobacterium tuberculosis/africanum*	Positive	1198	1198	/

S19BD03619	*Mycobacterium tuberculosis/africanum*	Positive	1549	1549	/
S19BD03659	*Mycobacterium tuberculosis/africanum*	Positive	53	53	/
S19BD03692	*Mycobacterium tuberculosis/africanum*	Positive	1535	1535	/
S19BD03871	*Mycobacterium tuberculosis/africanum*	Positive	144	144	/
S19BD04031	*Mycobacterium tuberculosis/africanum*	Positive	48	48	/
S19BD04034	*Mycobacterium tuberculosis/africanum*	Positive	20	20	/
S19BD04122	*Mycobacterium bovis/BCG*	Positive	482	482	/
S19BD04317	*Mycobacterium tuberculosis/africanum*	Positive	1	1	/
S19BD04437	*Mycobacterium tuberculosis/africanum*	Positive	**768**	**1800**	**31**
S19BD04454	*Mycobacterium tuberculosis/africanum*	Positive	26	26	/
S19BD04462	*Mycobacterium tuberculosis/africanum*	Positive	**68**	**44**	**8**
S19BD04840	*Mycobacterium tuberculosis/africanum*	Positive	**50**	**53**	**31**
S19BD04941	*Mycobacterium tuberculosis/africanum*	Positive	4	4	/
S19BD04942	*Mycobacterium tuberculosis/africanum*	Positive	4	4	/
S19BD04943	*Mycobacterium tuberculosis/africanum*	Positive	4	4	/
S19BD05114	*Mycobacterium bovis/BCG*	Positive	481	481	/
S19BD05219	*Mycobacterium tuberculosis/africanum*	Positive	1	1	/
S19BD05637	*Mycobacterium bovis/BCG*	Positive	1047	1047	/
S19BD05638	*Mycobacterium tuberculosis/africanum*	Positive	**50**	**53**	**31**
S19BD05639	*Mycobacterium tuberculosis/africanum*	Positive	25	25	/
S19BD05777	*Mycobacterium tuberculosis/africanum*	Positive	52	52	/
S19BD05778	*Mycobacterium tuberculosis/africanum*	Positive	52	52	/
S19BD06004	*Mycobacterium tuberculosis/africanum*	Positive	181	181	/
S19BD06016	*Mycobacterium tuberculosis/africanum*	Positive	336	336	/
S19BD06018	*Mycobacterium bovis/BCG*	Positive	481	481	/
S19BD06295	*Mycobacterium tuberculosis/africanum*	Positive	53	53	/
S19BD06296	*Mycobacterium tuberculosis/africanum*	Positive	26	26	/

S19BD06838	*Mycobacterium tuberculosis/africanum*	Positive	853	853	/
S19BD07348	*Mycobacterium tuberculosis/africanum*	Positive	53	53	/
S19BD07397	*Mycobacterium tuberculosis/africanum*	Positive	17	17	/
S19BD07681	*Mycobacterium bovis/BCG*	Positive	482	482	/
S19BD07775	*Mycobacterium tuberculosis/africanum*	Positive	1	1	/
S19BD07878	*Mycobacterium tuberculosis/africanum*	Positive	149	149	/
S19BD07881	*Mycobacterium tuberculosis/africanum*	Positive	42	42	/
S19BD07893	*Mycobacterium tuberculosis/africanum*	Positive	52	52	/
S19BD07950	*Mycobacterium tuberculosis/africanum*	Positive	53	53	/
S19BD08025	*Mycobacterium tuberculosis/africanum*	Positive	25	25	/
S19BD08414	*Mycobacterium tuberculosis/africanum*	Positive	1570	1570	/
S19BD08608	*Mycobacterium tuberculosis/africanum*	Positive	167	167	/
S19BD08697	*Mycobacterium tuberculosis/africanum*	Positive	58	58	/
S19BD08985	*Mycobacterium bovis/BCG*	Positive	482	482	/
S19BD09037	*Mycobacterium tuberculosis/africanum*	Positive	53	53	/
S19BD09173	*Mycobacterium tuberculosis/africanum*	Positive	93	93	/
S19BD09201	*Mycobacterium tuberculosis/africanum*	Positive	1	1	/
S19BD09267	*Mycobacterium tuberculosis/africanum*	Positive	37	37	/
S19BD09360	*Mycobacterium tuberculosis/africanum*	Positive	47	47	/
S19BD09501	*Mycobacterium tuberculosis/africanum*	Positive	42	42	/
S20BD00190	*Mycobacterium tuberculosis/africanum*	Positive	172	172	/
S20BD00314	*Mycobacterium tuberculosis/africanum*	Positive	**50**	**53**	**31**
S20BD00315	*Mycobacterium bovis/BCG*	Positive	482	482	/
S20BD00328	*Mycobacterium tuberculosis/africanum*	Positive	53	53	/
S20BD00569	*Mycobacterium tuberculosis/africanum*	Positive	853	853	/
S20BD00570	*Mycobacterium tuberculosis/africanum*	Positive	1056	1056	/
S20BD00934	*Mycobacterium tuberculosis/africanum*	Positive	822	822	/

S20BD00953	*Mycobacterium bovis/BCG*	Positive	482	482	/
S20BD01134	*Mycobacterium tuberculosis/africanum*	Positive	61	61	/
S20BD01357	*Mycobacterium tuberculosis/africanum*	Positive	25	25	/
S20BD01537	*Mycobacterium tuberculosis/africanum*	Positive	**50**	**53**	**31**
NC_CJ00001	*Campylobacter jejuni*	Negative	/	/	/
NC_CJ00002	*Campylobacter jejuni*	Negative	/	/	/
NC_CJ00003	*Campylobacter jejuni*	Negative	/	/	/
NC_CJ00004	*Campylobacter jejuni*	Negative	/	/	/
NC_CJ00005	*Campylobacter jejuni*	Negative	/	/	/
NC_SE00001	*Salmonella enteritidis*	Negative	/	/	/
NC_SE00002	*Salmonella enteritidis*	Negative	/	/	/
NC_SE00003	*Salmonella enteritidis*	Negative	/	/	/
NC_SE00004	*Salmonella enteritidis*	Negative	/	/	/
NC_SE00005	*Salmonella enteritidis*	Negative	/	/	/
NC_SE00006	*Salmonella enteritidis*	Negative	/	/	/
NC_NM00001	*Neisseria meningititis*	Negative	/	/	/
NC_NM00002	*Neisseria meningititis*	Negative	/	/	/
NC_NM00003	*Neisseria meningititis*	Negative	/	/	/
NC_NM00004	*Neisseria meningititis*	Negative	/	/	/
NC_NM00005	*Neisseria meningititis*	Negative	/	/	/

Table S8: Samples used for the validation of the SNP-barcoding assay.

The first and second columns contain the name and species of samples, respectively. The third column contains the sample class and the fourth column contains the lowest lineage information for each sample. Negative samples did not have associated lineage information.

Sample name	Species	Sample class	(Sub-)Lineage
ERR025455	MTBC	Positive	lineage4.3.2
ERR025842	MTBC	Positive	lineage4.2.2
ERR027453	MTBC	Positive	lineage4.9
ERR028608	MTBC	Positive	lineage4.4.1.2
ERR028617	MTBC	Positive	lineage4.2.2.1
ERR028627	MTBC	Positive	lineage4.1.2
ERR036187	MTBC	Positive	lineage4.3.4.1
ERR037533	MTBC	Positive	lineage4.7
ERR038253	MTBC	Positive	lineage4.1.1.3
ERR038277	MTBC	Positive	lineage4.1.1
ERR038749	MTBC	Positive	lineage4.6.1.2
ERR039338	MTBC	Positive	lineage4.3.1
ERR040119	MTBC	Positive	lineage3.1.2.1
ERR046838	MTBC	Positive	lineage4.6.2.1
ERR046897	MTBC	Positive	lineage4.4.1.1
ERR046933	MTBC	Positive	lineage5
ERR046937	MTBC	Positive	lineage4.1.2.1
ERR046941	MTBC	Positive	lineage3.1.2.2
ERR046954	MTBC	Positive	lineageBOV_AFRI
ERR047001	MTBC	Positive	lineage1.1.2
ERR047004	MTBC	Positive	lineage4.6.2.2
ERR047880	MTBC	Positive	lineage1.1.1
ERR047890	MTBC	Positive	lineage4.1
ERR072039	MTBC	Positive	lineage4
ERR1035199	MTBC	Positive	lineage4.3.4.2
ERR234111	MTBC	Positive	lineage3.1
ERR234164	MTBC	Positive	lineage1.2.2
ERR234173	MTBC	Positive	lineage4.6.1.1
ERR234180	MTBC	Positive	lineage4.8
ERR234181	MTBC	Positive	lineage3
ERR234190	MTBC	Positive	lineage2.2.1.1
ERR234216	MTBC	Positive	lineage2.1
ERR234251	MTBC	Positive	lineage4.3.3
ERR234257	MTBC	Positive	lineage6
ERR234268	MTBC	Positive	lineage4.1.1.1
ERR234272	MTBC	Positive	lineage1.2.1
ERR245674	MTBC	Positive	lineage1.1.3
ERR245684	MTBC	Positive	lineage3.1.1
ERR245749	MTBC	Positive	lineage4.3.4.2.1
ERR245762	MTBC	Positive	lineage4.3.2.1
SRR671762	MTBC	Positive	lineage4.4.2

SRR671797	MTBC	Positive	lineage2.2.2
SRR671816	MTBC	Positive	lineage2.2.1
SRR671872	MTBC	Positive	lineage4.5
NC_CJ00001	*Campylobacter jejuni*	Negative	/
NC_CJ00002	*Campylobacter jejuni*	Negative	/
NC_CJ00003	*Campylobacter jejuni*	Negative	/
NC_CJ00004	*Campylobacter jejuni*	Negative	/
NC_CJ00005	*Campylobacter jejuni*	Negative	/
NC_SE00001	*Salmonella enteritidis*	Negative	/
NC_SE00002	*Salmonella enteritidis*	Negative	/
NC_SE00003	*Salmonella enteritidis*	Negative	/
NC_SE00004	*Salmonella enteritidis*	Negative	/
NC_SE00005	*Salmonella enteritidis*	Negative	/
NC_SE00006	*Salmonella enteritidis*	Negative	/
NC_NM00001	*Neisseria meningititis*	Negative	/
NC_NM00002	*Neisseria meningititis*	Negative	/
NC_NM00003	*Neisseria meningititis*	Negative	/
NC_NM00004	*Neisseria meningititis*	Negative	/
NC_NM00005	*Neisseria meningititis*	Negative	/

92

Table S9: Samples used for the core gene multilocus sequence typing (cgMLST) validation.

The first, second, and third column list the sample name, sample species, and negative control status in the validation of the assay, respectively.

Name	Species	Negative control
ERR038277	MTBC	Positive
ERR038749	MTBC	Positive
ERR046933	MTBC	Positive
ERR046941	MTBC	Positive
ERR046954	MTBC	Positive
ERR047001	MTBC	Positive
ERR245674	MTBC	Positive
ERR245684	MTBC	Positive
S07MY01004	MTBC	Positive
S08MY00057	MTBC	Positive
S13MY00376	MTBC	Positive
S13MY02475	MTBC	Positive
S16BD00823	MTBC	Positive
S17BD00461	MTBC	Positive
S18BD02559	MTBC	Positive
S19BD00094	MTBC	Positive
S19BD00245	MTBC	Positive
S19BD00778	MTBC	Positive
S19BD00845	MTBC	Positive
S19BD00875	MTBC	Positive
S19BD01110	MTBC	Positive
S19BD01204	MTBC	Positive
S19BD01466	MTBC	Positive
S19BD01541	MTBC	Positive
S19BD01779	MTBC	Positive
S19BD02343	MTBC	Positive
S19BD03152	MTBC	Positive
S19BD03238	MTBC	Positive
S19BD03408	MTBC	Positive
S19BD04031	MTBC	Positive
S19BD04454	MTBC	Positive
S19BD05549	MTBC	Positive
S19BD05778	MTBC	Positive
S19BD06004	MTBC	Positive
S19BD06295	MTBC	Positive
S19BD09201	MTBC	Positive
SRR1915477	MTBC	Positive
SRR5486073	MTBC	Positive
SRR6045301	MTBC	Positive
SRR6046675	MTBC	Positive
SRR671762	MTBC	Positive
SRR671797	MTBC	Positive
NC_CJ00001	*Campylobacter jejuni*	Negative
NC_CJ00002	*Campylobacter jejuni*	Negative

NC_CJ00003	*Campylobacter jejuni*	Negative
NC_CJ00004	*Campylobacter jejuni*	Negative
NC_CJ00005	*Campylobacter jejuni*	Negative
NC_NM00001	*Neisseria meningitidis*	Negative
NC_NM00002	*Neisseria meningitidis*	Negative
NC_NM00003	*Neisseria meningitidis*	Negative
NC_NM00004	*Neisseria meningitidis*	Negative
NC_NM00005	*Neisseria meningitidis*	Negative
NC_SE00001	*Salmonella enteritidis*	Negative
NC_SE00002	*Salmonella enteritidis*	Negative
NC_SE00003	*Salmonella enteritidis*	Negative
NC_SE00004	*Salmonella enteritidis*	Negative
NC_SE00005	*Salmonella enteritidis*	Negative
NC_SE00006	*Salmonella enteritidis*	Negative

Table S10: Samples used for the validation of the *csb* RD assay.

The first and second columns lists the sample name and species of the included samples, respectively. The four last columns display whether each sample was used as a positive or negative control for validation of the assay for each of the targeted species.

Sample name	Species	Sample class (for each validated species)			
		M. bovis BCG	*M. tuberculosis*	*M. africanum*	*M. bovis*
SRR6044858	*Mycobacterium africanum*	Negative	Negative	Positive	Negative
SRR6044939	*Mycobacterium africanum*	Negative	Negative	Positive	Negative
SRR6045015	*Mycobacterium africanum*	Negative	Negative	Positive	Negative
SRR6045301	*Mycobacterium africanum*	Negative	Negative	Positive	Negative
SRR6045425	*Mycobacterium africanum*	Negative	Negative	Positive	Negative
SRR6045962	*Mycobacterium africanum*	Negative	Negative	Positive	Negative
SRR6046131	*Mycobacterium africanum*	Negative	Negative	Positive	Negative
SRR6046235	*Mycobacterium africanum*	Negative	Negative	Positive	Negative
SRR6046334	*Mycobacterium africanum*	Negative	Negative	Positive	Negative
SRR6046675	*Mycobacterium africanum*	Negative	Negative	Positive	Negative
SRR6046695	*Mycobacterium africanum*	Negative	Negative	Positive	Negative
SRR6046725	*Mycobacterium africanum*	Negative	Negative	Positive	Negative
S19BD01049	*Mycobacterium bovis*	Negative	Negative	Negative	Positive
S19BD02380	*Mycobacterium bovis*	Negative	Negative	Negative	Positive
SRR5486073	*Mycobacterium bovis*	Negative	Negative	Negative	Positive
SRR5486074	*Mycobacterium bovis*	Negative	Negative	Negative	Positive
SRR5486075	*Mycobacterium bovis*	Negative	Negative	Negative	Positive
SRR5486076	*Mycobacterium bovis*	Negative	Negative	Negative	Positive
SRR5486077	*Mycobacterium bovis*	Negative	Negative	Negative	Positive
SRR5486078	*Mycobacterium bovis*	Negative	Negative	Negative	Positive
SRR5486079	*Mycobacterium bovis*	Negative	Negative	Negative	Positive
SRR5486080	*Mycobacterium bovis*	Negative	Negative	Negative	Positive
SRR5486081	*Mycobacterium bovis*	Negative	Negative	Negative	Positive
SRR5486082	*Mycobacterium bovis*	Negative	Negative	Negative	Positive
SRR5486083	*Mycobacterium bovis*	Negative	Negative	Negative	Positive
SRR5486084	*Mycobacterium bovis*	Negative	Negative	Negative	Positive
SRR5486085	*Mycobacterium bovis*	Negative	Negative	Negative	Positive
SRR5486086	*Mycobacterium bovis*	Negative	Negative	Negative	Positive
S19BD00167	*Mycobacterium bovis BCG*	Positive	Negative	Negative	Negative
S19BD00168	*Mycobacterium bovis BCG*	Positive	Negative	Negative	Negative
S19BD00290	*Mycobacterium bovis BCG*	Positive	Negative	Negative	Negative
S19BD00819	*Mycobacterium bovis BCG*	Positive	Negative	Negative	Negative
S19BD00844	*Mycobacterium bovis BCG*	Positive	Negative	Negative	Negative
S19BD00936	*Mycobacterium bovis BCG*	Positive	Negative	Negative	Negative
S19BD02423	*Mycobacterium bovis BCG*	Positive	Negative	Negative	Negative
S19BD02579	*Mycobacterium bovis BCG*	Positive	Negative	Negative	Negative
SRR1915476	*Mycobacterium bovis BCG*	Positive	Negative	Negative	Negative
SRR1915477	*Mycobacterium bovis BCG*	Positive	Negative	Negative	Negative
SRR1915478	*Mycobacterium bovis BCG*	Positive	Negative	Negative	Negative
SRR1915488	*Mycobacterium bovis BCG*	Positive	Negative	Negative	Negative
SRR1915489	*Mycobacterium bovis BCG*	Positive	Negative	Negative	Negative
S15BD02575	*Mycobacterium tuberculosis*	Negative	Positive	Negative	Negative
S15BD05110	*Mycobacterium tuberculosis*	Negative	Positive	Negative	Negative
S15BD09765	*Mycobacterium tuberculosis*	Negative	Positive	Negative	Negative
S16BD00823	*Mycobacterium tuberculosis*	Negative	Positive	Negative	Negative
S16BD06129	*Mycobacterium tuberculosis*	Negative	Positive	Negative	Negative

S16BD06161	*Mycobacterium tuberculosis*	Negative	Positive	Negative	Negative
S16BD07601	*Mycobacterium tuberculosis*	Negative	Positive	Negative	Negative
S16BD08755	*Mycobacterium tuberculosis*	Negative	Positive	Negative	Negative
S17BD00461	*Mycobacterium tuberculosis*	Negative	Positive	Negative	Negative
S17BD00631	*Mycobacterium tuberculosis*	Negative	Positive	Negative	Negative
S17BD00744	*Mycobacterium tuberculosis*	Negative	Positive	Negative	Negative
S17BD01584	*Mycobacterium tuberculosis*	Negative	Positive	Negative	Negative

Table S11: *In silico* modified samples for the validation of the SNP-based antimicrobial resistance detection at the positions covered by molecular methods.

List of the samples used for the *in silico* modification of samples at the positions covered by molecular methods.

**Sample name**
S07MY01004
S07MY01281
S08MY00593
S08MY00891
S08MY01602
S11MY00210
S13MY00376
S13MY01686
S13MY02475
S14MY00227
S15MY00419
S15BD09765
S16BD00996
S18BD02332
S18BD02340
S18BD02391
S18BD02531
S18BD02535
S18BD02559
S18BD02600
S18BD03040
S18BD03057
S18BD03223

Table S12: Mutations from conventionally characterized positions inserted *in silico* in samples for the validation of the SNP-based antimicrobial resistance assay.

Mutations sampled from positions characterized by conventional methods that were inserted *in silico* into sequencing datasets.

Gene	Mutation	Type	Genomic position (and DNA mutation)
embB	Met-306-Val	Amino-acid	4247429 (A>G)
gyrA	Asp-94-Gly	Amino-acid	7582 (A>G)
katG	Ser-315-Thr	Amino-acid	2155168 (C>G)
rpoB	His-445-Arg	Amino-acid	761140 (A>G)
rrs	A(1401)G	Nucleotide	1473246 (A>G)
Rv1482c-fabG1	C(-15)T	Promoter	1673425 (C>T)

Table S13: *In silico* modified samples for the validation of the SNP-based antimicrobial resistance detection at the positions randomly selected from the database.

List of the samples used for the *in silico* modification of samples at the positions randomly selected from the AMR database.

Sample name
S07MY01004
S07MY01281
S08MY00057
S08MY00593
S08MY00891
S08MY01602
S09MY00391
S10MY00981
S11MY00210
S12MY01730
S12MY01752
S13MY00376
S13MY01686
S13MY02475
S14MY00112
S14MY00227
S14MY02137
S14MY02259
S15BD02575
S15BD05110
S15BD08902
S15BD09765
S15MY00419
S16BD00823
S16BD00996
S16BD06129
S16BD06161
S16BD07601
S16BD08755
S17BD00461
S17BD00631
S17BD00744
S17BD01584
S17BD03113
S17BD04315
S17BD04669
S17BD04916
S18BD00369
S18BD00575
S18BD01935
S18BD02332
S18BD02340

S18BD02391
S18BD02531
S18BD02535
S18BD02559
S18BD02600
S18BD03002
S18BD03040
S18BD03057
S18BD03223

Table S14: Mutations randomly selected from the database of AMR-related mutations and inserted samples.

Mutations sampled from positions randomly selected from the AMR mutation database, which were inserted *in silico* into samples read data. The first column lists the targeted gene or locus. The second and third column list the target mutation and type. The fourth and fifth columns list the genomic position in the H37Rv reference genome and mutated nucleotide(s), respectively.

Gene	Mutation	Type	Genomic position	Mutated nucleotide(s)
ahpC	Gly-45-Ser	Amino-acid	2726325	G>A
ahpC	Ala-187-Pro	Amino-acid	2726751	G>C
ahpC	Ala-195-Ser	Amino-acid	2726775	G>T
ahpC	Pro-44-Arg	Amino-acid	2726323	C>G
ahpC	Thr-105-Met	Amino-acid	2726506	C>T
ahpC	Glu-160-Lys	Amino-acid	2726670	G>A
ahpC	Leu-191-Phe	Amino-acid	2726763	C>T
ahpC	Val-158-Phe	Amino-acid	2726664	G>T
alr	Phe-4-Leu	Amino-acid	3841409	G>C
alr	Leu-113-Arg	Amino-acid	3841083	A>C
alr	Met-343-Thr	Amino-acid	3840393	A>G
atpE	Asp-28-Val	Amino-acid	1461127	A>T
atpE	Ala-63-Pro	Amino-acid	1461231	G>C
atpE	Glu-61-Asp	Amino-acid	1461227	G>C
atpE	Ile-66-Met	Amino-acid	1461242	C>G
cycA	Gly-122-Ser	Amino-acid	1931093	C>T
dfrA	Ser-66-Cys	Amino-acid	3073414	T>A
dfrA	Val-54-Ala	Amino-acid	3073449	A>G
dfrA	Gln-28-Leu	Amino-acid	3073527	T>A
dfrA	Cys-110-Arg	Amino-acid	3073282	A>G
drrA	Arg-262-Gly	Amino-acid	3272997	A>G
embA	Ser-77-Pro	Amino-acid	4243461	T>C
embA	Thr-728-Ile	Amino-acid	4245415	C>T
embA	Ala-1016-Ser	Amino-acid	4246278	G>T
embA	Tyr-307-His	Amino-acid	4244151	T>C
embA	Val-1078-Met	Amino-acid	4246464	G>A
embA	Ala-109-Thr	Amino-acid	4243557	G>A
embA	Leu-233-Met	Amino-acid	4243929	C>A
embA	Trp-306-Arg	Amino-acid	4244148	T>A
embA	Asn-54-Ser	Amino-acid	4243393	A>G
embB	Thr-1069-Pro	Amino-acid	4249718	A>C
embB	Arg-147-Cys	Amino-acid	4246952	C>T
embB	Val-135-Met	Amino-acid	4246916	G>A
embB	Cys-361-Ser	Amino-acid	4247595	G>C
embB	Trp-332-Arg	Amino-acid	4247507	T>C
embB	Met-1049-Ile	Amino-acid	4249660	G>A
embB	Gly-836-Arg	Amino-acid	4249019	G>A
embB	Gln-51-Pro	Amino-acid	4246665	A>C

embB	Ser-658-Arg	Amino-acid	4248487	C>G
embB	Ile-1006-Met	Amino-acid	4249531	C>G
embB	Met-462-Thr	Amino-acid	4247898	T>C
embB	Leu-370-Arg	Amino-acid	4247622	T>G
embB	Leu-348-Pro	Amino-acid	4247556	T>C
embB	Asp-1024-Asn	Amino-acid	4249583	G>A
embC	Val-363-Ile	Amino-acid	4240949	G>A
embC	Pro-732-Ala	Amino-acid	4242056	C>G
embC	Gln-742-His	Amino-acid	4242088	G>T
embC	Thr-1079-Ala	Amino-acid	4243097	A>G
embC	Ala-597-Thr	Amino-acid	4241651	G>A
embC	Gly-909-Glu	Amino-acid	4242588	G>A
embC	Phe-286-Leu	Amino-acid	4240720	C>G
embC	Thr-786-Ala	Amino-acid	4242218	A>G
embC	Ala-116-Thr	Amino-acid	4240208	G>A
ethR	Phe-110-Leu	Amino-acid	4327878	C>G
ethR	Ala-95-Thr	Amino-acid	4327831	G>A
fabG1	Thr-4-Ile	Amino-acid	1673450	C>T
fabG1	Ala-215-Thr	Amino-acid	1674082	G>A
fabG1	Ser-126-Asn	Amino-acid	1673816	G>A
fabG1	Pro-81-Ala	Amino-acid	1673680	C>G
fabG1	Asn-24-Ser	Amino-acid	1673510	A>G
fabG1	Glu-7-Lys	Amino-acid	1673458	G>A
folC	Ile-43-Ala	Amino-acid	2747471, 2747472	AT>GC
folC	Ser-150-Gly	Amino-acid	2747151	T>C
folC	Glu-40-Ala	Amino-acid	2747480	T>G
folC	Glu-153-Gln	Amino-acid	2747142	C>G
folC	Arg-49-Leu	Amino-acid	2747453	C>A
furA	Leu-68-Phe	Amino-acid	2156389	C>A
gid	Ser-149-Arg	Amino-acid	4407758	T>G
gid	Gly-164-Cys	Amino-acid	4407713	C>A
gid	Glu-92-Asp	Amino-acid	4407927	T>A
gid	Leu-49-Pro	Amino-acid	4408057	A>G
gid	Gly-30-Arg	Amino-acid	4408115	C>G
gid	Val-110-Gly	Amino-acid	4407874	A>C
gid	Leu-16-Arg	Amino-acid	4408156	A>C
gid	Gly-117-Glu	Amino-acid	4407853	C>T
gyrA	Thr-80-Ala	Amino-acid	7539	A>G
gyrA	Gly-247-Ser	Amino-acid	8040	G>A
gyrA	Asp-94-His	Amino-acid	7581	G>C
gyrA	Glu-21-Gln	Amino-acid	7362	G>C
gyrA	Ala-90-Val	Amino-acid	7570	C>T
gyrA	Gly-88-Cys	Amino-acid	7563	G>T
gyrA	Ser-95-Thr	Amino-acid	7585	G>C
gyrA	Ala-74-Ser	Amino-acid	7521	G>T
gyrB	Asp-461-Asn	Amino-acid	6620	G>A
gyrB	Ala-504-Val	Amino-acid	6750	C>T
gyrB	Gly-470-Ala	Amino-acid	6648	G>C
gyrB	Asn-499-Asp	Amino-acid	6734	A>G
gyrB	Thr-500-Ile	Amino-acid	6738	C>T
gyrB	Glu-501-Val	Amino-acid	6741	A>T
gyrB	Glu-459-Lys	Amino-acid	6614	G>A

gyrB	Asp-494-Ala	Amino-acid	6720	A>C
inhA	Ile-194-Thr	Amino-acid	1674782	T>C
inhA	Gly-183-Arg	Amino-acid	1674748	G>C
inhA	Pro-107-Ser	Amino-acid	1674520	C>T
inhA	Val-78-Ala	Amino-acid	1674434	T>C
inhA	Ile-21-Thr	Amino-acid	1674263	T>C
inhA	Ser-94-Ala	Amino-acid	1674481	T>G
inhA	Gly-141-Arg	Amino-acid	1674622	G>A
katG	Gln-36-Pro	Amino-acid	2156005	T>G
katG	Val-23-Leu	Amino-acid	2156045	C>A
katG	Thr-308-Ala	Amino-acid	2155190	T>C
katG	Leu-159-Pro	Amino-acid	2155636	A>G
katG	Ser-527-Leu	Amino-acid	2154532	G>A
katG	Val-47-Ile	Amino-acid	2155973	C>T
katG	Ala-551-Ser	Amino-acid	2154461	C>A
katG	Val-739-Met	Amino-acid	2153897	C>T
katG	Val-633-Ala	Amino-acid	2154214	A>G
mshA	Ala-187-Val	Amino-acid	575907	C>T
mshA	Asn-111-Ser	Amino-acid	575679	A>G
panD	Ile-49-Val	Amino-acid	4044137	T>C
panD	Met-117-Ile	Amino-acid	4043931	C>G
panD	Ile-115-Thr	Amino-acid	4043938	A>G
panD	Pro-134-Ser	Amino-acid	4043882	G>A
Rv2535c	Leu-44-Pro	Amino-acid	2860288	A>G
pncA	Gln-10-Pro	Amino-acid	2289213	T>G
pncA	Gly-78-Val	Amino-acid	2289009	C>A
pncA	Asp-110-Glu	Amino-acid	2288912	G>C
pncA	His-71-Asn	Amino-acid	2289031	G>T
pncA	Pro-77-Ser	Amino-acid	2289013	G>A
pncA	His-51-Gln	Amino-acid	2289089	G>C
pncA	Leu-4-Ser	Amino-acid	2289231	A>G
pncA	Trp-119-Leu	Amino-acid	2288886	C>A
pncA	Val-7-Gly	Amino-acid	2289222	A>C
pncA	Glu-37-Val	Amino-acid	2289132	T>A
pncA	His-57-Arg	Amino-acid	2289072	T>C
pncA	Ile-133-Phe	Amino-acid	2288845	T>A
rplC	Cys-154-Asn	Amino-acid	801268, 801269	TG>AA
rpoA	Arg-186-Cys	Amino-acid	3877952	G>A
rpoA	Glu-319-Lys	Amino-acid	3877553	C>T
rpoB	Met-434-Ile	Amino-acid	761108	G>T
rpoB	Ser-431-Thr	Amino-acid	761098	G>C
rpoB	Pro-454-Leu	Amino-acid	761167	C>T
rpoB	Ala-544-Val	Amino-acid	761437	C>T
rpoB	His-445-Cys	Amino-acid	761139, 761140	CA>TG
rpoB	Asp-435-Asn	Amino-acid	761109	G>A
rpoB	Leu-893-Arg	Amino-acid	762484	T>G
rpoB	Leu-80-Val	Amino-acid	760044	C>G
rpoB	Thr-350-Ile	Amino-acid	760855	C>T
rpoC	Val-483-Gly	Amino-acid	764817	T>G
rpoC	Trp-484-Gly	Amino-acid	764819	T>G
rpoC	Gly-594-Glu	Amino-acid	765150	G>A
rpoC	Val-1252-Leu	Amino-acid	767123	G>T

rpoC	Pro-1040-Ser	Amino-acid	766487	C>T
rpoC	Ala-1044-Val	Amino-acid	766500	C>T
rpoC	Asn-698-Ser	Amino-acid	765462	A>G
rpoC	Ala-172-Val	Amino-acid	763884	C>T
rpsA	Asp-123-Ala	Amino-acid	1833909	A>C
rpsA	Thr-5-Ser	Amino-acid	1833555	C>G
rpsL	Thr-40-Ile	Amino-acid	781678	C>T
rpsL	Lys-88-Arg	Amino-acid	781822	A>G
rpsL	Lys-43-Thr	Amino-acid	781687	A>C
Rv0678	Glu-138-Gly	Amino-acid	779402	A>G
Rv0678	Ser-68-Gly	Amino-acid	779191	A>G
Rv0678	Arg-94-Gln	Amino-acid	779270	G>A
Rv2688c	Cys-213-Arg	Amino-acid	3005014	A>G
sahH	Thr-220-Ala	Amino-acid	3628990	T>C
thyA	Gln-162-Ala	Amino-acid	3073987, 3073988	TG>GC
thyA	Gly-91-Glu	Amino-acid	3074200	C>T
thyA	Val-50-Ala	Amino-acid	3074323	A>G
thyA	Tyr-251-Cys	Amino-acid	3073720	T>C
thyA	Leu-163-Pro	Amino-acid	3073984	A>G
thyA	Pro-253-Ala	Amino-acid	3073715	G>C
thyA	Gly-95-Cys	Amino-acid	3074189	C>A
thyA	Met-141-Arg	Amino-acid	3074050	A>C
ubiA	Leu-224-Phe	Amino-acid	4269162	C>A
ubiA	Glu-149-Asp	Amino-acid	4269387	T>A
ubiA	Ala-249-Gly	Amino-acid	4269088	G>C
ubiA	Arg-240-Cys	Amino-acid	4269116	G>A
ubiA	Gly-268-Asp	Amino-acid	4269031	C>T
ubiA	Val-188-Ala	Amino-acid	4269271	A>G
ubiA	Met-180-Val	Amino-acid	4269296	T>C
ubiA	Ala-237-Val	Amino-acid	4269124	G>A
alr-Rv3424c	G(-7)T	Promoter	3841427	C>T
eis-Rv2417c	G(-2)A	Promoter	2715334	C>A
eis-Rv2417c	C(-10)A	Promoter	2715342	G>A
eis-Rv2417c	C(-37)T	Promoter	2715369	G>T
embC-embA	C(-8)T	Promoter	4243225	C>T
embC-embA	C(-15)G	Promoter	4243218	C>G
embC-embA	C(-29)A	Promoter	4243204	G>A
embC-embA	G(-43)C	Promoter	4243190	G>C
embC-embA	C(-59)A	Promoter	4243174	C>A
embC-embA	C(-73)T	Promoter	4243160	C>T
katG-furA	A(-13)C	Promoter	2156124	T>C
katG-furA	T(-35)G	Promoter	2156146	A>G
oxyR'-aphC	A(-12)T	Promoter	2726181	A>T
oxyR'-aphC	C(-39)T	Promoter	2726154	C>T
oxyR'-aphC	G(-48)A	Promoter	2726145	G>A
oxyR'-aphC	C(-57)T	Promoter	2726136	C>T
oxyR'-aphC	C(-72)T	Promoter	2726121	C>T
oxyR'-aphC	A(-83)G	Promoter	2726110	A>G
oxyR'-aphC	A(-98)C	Promoter	2726095	A>C
pncA-	A(-7)C	Promoter	2289248	T>C
pncA-	G(-19)T	Promoter	2289260	C>T
pncA-	C(-33)A	Promoter	2289274	G>A

PPE52-nuoA	G(-314)T	Promoter	3511368	G>T
Rv0666-rpoB	T(-6)C	Promoter	759801	T>C
Rv0666-rpoB	A(-53)G	Promoter	759754	A>G
Rv0666-rpoB	C(-62)T	Promoter	759745	C>T
Rv0666-rpoB	C(-73)T	Promoter	759734	C>T
Rv1482c-	T(-8)C	Promoter	1673432	T>C
Rv1482c-	G(-17)T	Promoter	1673423	G>T
Rv1482c-	C(-40)T	Promoter	1673400	C>T
Rv1482c-	G(-67)T	Promoter	1673373	C>T
Rv1482c-	G(-77)A	Promoter	1673363	G>A
Rv1910c-	C(-7)A	Promoter	2156599	G>A
Rv1910c-	C(-15)A	Promoter	2156607	G>A
Rv3253c-	C(-7)T	Promoter	3633668	C>T
Rv3253c-	G(-30)T	Promoter	3633645	C>T
Rv3253c-	G(-49)T	Promoter	3633626	C>T
thyX-hsdM	C(-9)T	Promoter	3067954	C>T
thyX-hsdM	G(-23)C	Promoter	3067968	G>C
rrl	G(2270)C	Nucleotidic	1475927	G>C
rrl	G(2576)T	Nucleotidic	1476233	G>T
rrl	G(2746)A	Nucleotidic	1476403	G>A
rrs	A(1401)G	Nucleotidic	1473246	A>G
rrs	C(517)T	Nucleotidic	1472362	C>T
rrs	A(514)C	Nucleotidic	1472359	A>C
rrs	C(462)T	Nucleotidic	1472307	C>T
rrs	C(492)T	Nucleotidic	1472337	C>T
rrs	C(513)T	Nucleotidic	1472358	C>T
pncA	Stop-41	Stop codon	2289119	G>T
thyA	Stop-49	Stop codon	3074327	T>A
gid	Stop-128	Stop codon	4407820	A>T
ddn	Stop-130	Stop codon	3987233	C>G
whiB7	Stop-51	Stop codon	3568529	G>A

Table S15: Overview of the trimming and assembly statistics for the in-house sequenced MTBC samples

The first and second columns list the sample name and species, respectively. The third, fourth, and sixth columns list the coverage against the reference genome, the total number of read pairs and the total number of read pairs surviving trimming. The sixth, seventh and eight columns list the N50, number of contigs (after filtering on a kmer coverage of ≥10 and length ≥1000 bases) and total cumulative assembly length, respectively.

sample	species	coverage	Total reads pairs	Total reads pairs trimmed	n50	Contigs	Total length
S07MY01004	MTBC	100	1193546	1093082	82222	111	4337526
S07MY01281	MTBC	52	610304	564672	64265	126	4311540
S08MY00057	MTBC	72	817279	757566	73777	120	4341835
S08MY00593	MTBC	61	684270	634856	70622	121	4313448
S08MY00891	MTBC	61	717450	656206	78336	110	4318400
S08MY01602	MTBC	51	575783	523078	78342	111	4305818
S09MY00391	MTBC	89	999413	932522	64274	121	4337228
S10MY00981	MTBC	58	643923	599585	65473	127	4323045
S11MY00210	MTBC	56	642383	590269	82270	111	4343936
S12MY01730	MTBC	67	785581	722832	68715	115	4314486
S12MY01752	MTBC	70	817806	758249	70889	113	4348955
S13MY00376	MTBC	60	676435	629496	77328	107	4347605
S13MY01686	MTBC	55	640043	585562	78339	109	4276175
S13MY02475	*Mycobacterium africanum*	49	545072	496130	70921	113	4299790
S14MY00112	MTBC	60	665514	621500	68376	122	4319209
S14MY00227	MTBC	44	499692	447566	68621	124	4333813
S14MY02137	MTBC	57	639838	588232	69236	128	4315824
S14MY02259	MTBC	60	718695	665160	64264	115	4317667
S15BD02575	MTBC	75	839921	787160	68193	118	4335336
S15BD05110	MTBC	44	501736	456857	58345	132	4304074
S15BD08902	MTBC	56	636364	581586	70917	115	4330954
S15BD09765	MTBC	72	848322	793516	72729	123	4326755
S15MY00419	MTBC	43	492630	448780	68397	123	4312454
S16BD00823	MTBC	57	634610	580417	88436	104	4346729
S16BD00996	MTBC	68	796437	745854	70942	121	4328948
S16BD06129	MTBC	56	644560	591963	63979	120	4328829
S16BD06161	MTBC	46	521390	470454	63982	125	4319021
S16BD07601	MTBC	50	572778	530886	66117	122	4327852
S16BD08755	MTBC	33	419612	376346	57467	133	4325980
S17BD00461	MTBC	57	660702	600520	71440	122	4348552
S17BD00631	MTBC	52	578794	530103	72206	113	4315486
S17BD00744	MTBC	106	1174334	1098389	80919	110	4339014

S17BD01584	*Mycobacterium tuberculosis*	52	591347	539250	64692	131	4338598
S17BD03113	MTBC	45	569221	530188	62230	140	4297857
S17BD04315	MTBC	46	615968	570494	59174	145	4314878
S17BD04669	MTBC	37	505602	460962	62120	134	4335276
S17BD04916	*Mycobacterium tuberculosis/africanum*	68	808278	719376	65711	120	4331292
S18BD00369	MTBC	51	602411	532904	74350	120	4330438
S18BD00575	MTBC	140	1703681	1582516	81096	107	4339525
S18BD01935	MTBC	48	572984	529041	59131	138	4304027
S18BD02332	*Mycobacterium tuberculosis*	99	1104963	1035492	79828	133	4372981
S18BD02340	MTBC	50	545873	510014	81862	101	4339679
S18BD02391	MTBC	53	652501	602751	63794	149	4358495
S18BD02531	MTBC	38	429917	388463	61044	135	4327963
S18BD02535	*Mycobacterium tuberculosis*	77	831667	777992	67876	112	4336168
S18BD02559	MTBC	43	554118	508215	57496	145	4320414
S18BD02600	*Mycobacterium tuberculosis*	43	472878	434407	54270	138	4323294
S18BD03002	MTBC	86	1009931	937564	68385	111	4333590
S18BD03040	*Mycobacterium tuberculosis*	47	553813	523388	71055	125	4366617
S18BD03057	MTBC	81	900170	837817	69009	117	4341521
S18BD03223	MTBC	37	414146	370495	67328	121	4309280
S18BD08529	*Mycobacterium tuberculosis/africanum*	77	824595	766158	77815	115	4323361
S18BD09410	MTBC	58	674944	613065	78523	115	4307813
S18BD09562	MTBC	72	865373	760144	78397	113	4329704
S18BD09650	MTBC	48	596879	520834	73775	115	4352452
S18BD09708	*Mycobacterium tuberculosis/africanum*	68	786599	701144	81396	109	4282295
S18BD09709	*Mycobacterium tuberculosis/africanum*	79	924177	804280	74709	112	4335025
S19BD00079	*Mycobacterium tuberculosis/africanum*	78	826089	765015	82569	100	4332498
S19BD00094	*Mycobacterium tuberculosis/africanum*	64	728459	662233	78336	105	4296730
S19BD00140	*Mycobacterium tuberculosis/africanum*	110	1202847	1115788	80366	111	4337502
S19BD00141	*Mycobacterium tuberculosis/africanum*	98	1138530	1014959	82631	105	4336262
S19BD00167	*Mycobacterium bovis/BCG*	90	1093886	1026314	79024	105	4272876
S19BD00168	*Mycobacterium bovis/BCG*	82	919442	849666	79464	109	4269514
S19BD00198	*Mycobacterium tuberculosis/africanum*	92	1017466	942978	90155	96	4338983
S19BD00199	*Mycobacterium tuberculosis/africanum*	76	798206	734577	57550	154	4311638
S19BD00200	*Mycobacterium tuberculosis/africanum*	84	924891	840997	77704	116	4341624
S19BD00211	*Mycobacterium tuberculosis/africanum*	68	757524	692582	76103	107	4329006
S19BD00212	*Mycobacterium tuberculosis/africanum*	101	1139517	1064754	74350	109	4340520
S19BD00213	*Mycobacterium tuberculosis/africanum*	95	1040652	969627	86474	99	4341095
S19BD00245	MTBC	83	1035845	884611	77917	114	4361796

S19BD00282	*Mycobacterium tuberculosis/africanum*	210	2332493	2109880	107013	92	4306086
S19BD00290	*Mycobacterium bovis BCG*	114	1354970	1202625	81831	98	4265997
S19BD00357	*Mycobacterium tuberculosis/africanum*	92	1112325	972981	95925	96	4346626
S19BD00373	*Mycobacterium tuberculosis/africanum*	122	1327406	1201105	83930	104	4347375
S19BD00393	*Mycobacterium tuberculosis/africanum*	230	2991932	2684257	82034	481	7313932
S19BD00444	*Mycobacterium tuberculosis/africanum*	107	1295812	1141385	106405	104	4351841
S19BD00457	*Mycobacterium tuberculosis/africanum*	127	1611721	1479145	81272	103	4310321
S19BD00561	*Mycobacterium tuberculosis/africanum*	381	4727064	3988482	107404	74	4368025
S19BD00564	*Mycobacterium tuberculosis/africanum*	76	1032195	963620	72719	132	4331221
S19BD00646	*Mycobacterium tuberculosis/africanum*	269	3349715	2841368	84005	97	4339913
S19BD00656	*Mycobacterium tuberculosis/africanum*	68	767552	707675	70941	127	4374356
S19BD00752	*Mycobacterium tuberculosis/africanum*	81	877012	813244	72937	109	4331035
S19BD00777	*Mycobacterium tuberculosis/africanum*	79	875403	798832	82838	101	4353410
S19BD00778	MTBC	60	716620	678279	64280	140	4385295
S19BD00791	*Mycobacterium tuberculosis/africanum*	73	787568	741938	66018	128	4338718
S19BD00819	*Mycobacterium bovis BCG*	74	780073	727733	75798	113	4254000
S19BD00825	*Mycobacterium tuberculosis/africanum*	78	857064	812102	66185	119	4341275
S19BD00844	*Mycobacterium bovis BCG*	67	693155	645475	64279	118	4267633
S19BD00845	*Mycobacterium tuberculosis/africanum*	51	580374	550101	66162	123	4337524
S19BD00846	*Mycobacterium tuberculosis/africanum*	75	780933	733819	72093	113	4333717
S19BD00856	*Mycobacterium tuberculosis/africanum*	61	680850	628737	74644	123	4347645
S19BD00867	*Mycobacterium bovis*	65	707391	660346	81555	125	4318635
S19BD00875	*MTBC*	66	802115	728001	70740	124	4333132
S19BD00936	*Mycobacterium bovis BCG*	31	400586	355613	70865	129	4219461
S19BD00970	*Mycobacterium tuberculosis/africanum*	96	1222916	1104910	79224	118	4341777
S19BD01003	*Mycobacterium tuberculosis/africanum*	104	1275356	1123997	80788	108	4339287
S19BD01027	*Mycobacterium tuberculosis/africanum*	153	1845249	1654110	92227	97	4355827
S19BD01049	*Mycobacterium bovis*	76	904773	820732	98884	97	4293777
S19BD01090	*Mycobacterium tuberculosis/africanum*	126	1403181	1293814	84107	104	4347952
S19BD01110	MTBC	102	1208037	1099395	82150	121	4352956
S19BD01123	MTBC	111	1335331	1202768	93895	94	4326659
S19BD01129	*Mycobacterium tuberculosis/africanum*	120	1392914	1284702	87393	118	4375546
S19BD01163	*Mycobacterium tuberculosis/africanum*	92	1083819	1005095	80137	108	4347068
S19BD01204	*Mycobacterium tuberculosis/africanum*	105	1235816	1118326	70519	119	4373667
S19BD01217	*Mycobacterium tuberculosis/africanum*	135	1486235	1350877	82101	101	4341993

S19BD01228	*Mycobacterium tuberculosis/africanum*	94	1122365	989555	84037	98	4297052
S19BD01316	*Mycobacterium tuberculosis/africanum*	26	321301	287907	59746	140	4308859
S19BD01376	*Mycobacterium tuberculosis/africanum*	103	1206144	1082814	83282	117	4356809
S19BD01410	*Mycobacterium tuberculosis/africanum*	93	1086470	977867	77423	105	4343078
S19BD01454	MTBC	51	621425	574606	64134	128	4318605
S19BD01457	*Mycobacterium tuberculosis/africanum*	90	1043844	930952	90251	101	4346686
S19BD01466	*Mycobacterium tuberculosis/africanum*	136	1643238	1480763	99010	91	4342954
S19BD01492	*Mycobacterium tuberculosis/africanum*	40	472846	439700	66497	126	4339275
S19BD01541	*Mycobacterium tuberculosis/africanum*	64	738135	678018	82348	110	4335122
S19BD01544	*Mycobacterium tuberculosis/africanum*	47	792434	573134	72993	123	4323460
S19BD01586	*Mycobacterium tuberculosis/africanum*	44	776800	525778	70886	120	4275219
S19BD01591	MTBC	67	772599	724236	79752	108	4343812
S19BD01610	*Mycobacterium tuberculosis/africanum*	54	916216	612777	64674	130	4298574
S19BD01633	*Mycobacterium tuberculosis/africanum*	45	836726	593159	65563	138	4316632
S19BD01643	MTBC	67	1103405	823932	77476	105	4324259
S19BD01690	*Mycobacterium tuberculosis/africanum*	62	1125380	671096	78827	120	4315777
S19BD01691	*Mycobacterium tuberculosis/africanum*	46	854516	470435	64575	128	4319661
S19BD01749	MTBC	253	2840894	2560904	98885	106	4400754
S19BD01779	*Mycobacterium tuberculosis/africanum*	51	588825	533955	70615	114	4314316
S19BD01827	*Mycobacterium tuberculosis/africanum*	45	533449	480642	79659	115	4269751
S19BD01836	*Mycobacterium tuberculosis/africanum*	63	701391	659767	68686	134	4284460
S19BD01876	*Mycobacterium tuberculosis/africanum*	61	688403	630592	65441	131	4282481
S19BD01938	*Mycobacterium tuberculosis/africanum*	84	984668	878774	82366	108	4335148
S19BD01939	*Mycobacterium tuberculosis/africanum*	78	946309	848506	80662	115	4352749
S19BD01940	*Mycobacterium tuberculosis/africanum*	67	781507	705486	70995	114	4319420
S19BD02068	*Mycobacterium tuberculosis/africanum*	60	696187	625242	80774	112	4309453
S19BD02087	*Mycobacterium tuberculosis/africanum*	73	866796	783536	73775	120	4358286
S19BD02149	*Mycobacterium tuberculosis/africanum*	69	833559	733512	85504	97	4359901
S19BD02152	*Mycobacterium tuberculosis/africanum*	70	857776	778463	72917	135	4389742
S19BD02160	*Mycobacterium tuberculosis/africanum*	75	852362	773076	70923	122	4358000
S19BD02166	*Mycobacterium tuberculosis/africanum*	55	636322	585997	78368	113	4315516
S19BD02169	*Mycobacterium tuberculosis/africanum*	51	596856	547632	70869	120	4333779
S19BD02203	*Mycobacterium tuberculosis/africanum*	61	721959	635623	80625	111	4330240
S19BD02236	*Mycobacterium tuberculosis/africanum*	66	798088	699146	81857	112	4341958

S19BD02237	*Mycobacterium tuberculosis/africanum*	71	822375	736694	76272	116	4333769
S19BD02270	*Mycobacterium tuberculosis/africanum*	69	803991	714816	82348	107	4355720
S19BD02272	*Mycobacterium tuberculosis/africanum*	69	798056	733787	69282	130	4339235
S19BD02311	*Mycobacterium tuberculosis/africanum*	75	858028	792528	93899	113	4345840
S19BD02316	MTBC	66	774040	716389	77867	109	4352144
S19BD02343	*Mycobacterium tuberculosis/africanum*	68	859010	798137	76101	120	4355644
S19BD02344	*Mycobacterium tuberculosis/africanum*	83	943191	878311	69224	108	4341814
S19BD02364	*Mycobacterium tuberculosis/africanum*	74	837326	781952	69109	119	4341507
S19BD02380	*Mycobacterium bovis*	88	1011241	926956	80984	97	4272971
S19BD02390	*Mycobacterium tuberculosis/africanum*	91	1006666	944475	91141	99	4285646
S19BD02394	*Mycobacterium tuberculosis/africanum*	91	1039146	969230	70149	116	4368435
S19BD02423	*Mycobacterium bovis BCG*	67	767742	713925	76156	112	4264262
S19BD02475	*Mycobacterium tuberculosis/africanum*	76	854896	771610	81411	104	4337352
S19BD02502	*Mycobacterium tuberculosis/africanum*	88	1008995	935257	77867	107	4338232
S19BD02535	*Mycobacterium tuberculosis/africanum*	78	912449	824424	73777	114	4341371
S19BD02539	*Mycobacterium tuberculosis/africanum*	146	1545289	1448597	111486	90	4356003
S19BD02579	*Mycobacterium bovis BCG*	84	991164	915316	80740	101	4263888
S19BD02617	*Mycobacterium bovis BCG*	79	936577	842137	81932	100	4294627
S19BD02624	*Mycobacterium tuberculosis/africanum*	62	693643	630553	79457	107	4348688
S19BD02625	*Mycobacterium tuberculosis/africanum*	72	808323	765168	76156	118	4352524
S19BD02791	*Mycobacterium tuberculosis/africanum*	86	988390	911273	81010	102	4345192
S19BD02792	*Mycobacterium tuberculosis/africanum*	147	1928355	1794849	82324	107	4364584
S19BD02813	*Mycobacterium tuberculosis/africanum*	79	897668	816379	78321	102	4338883
S19BD02953	*Mycobacterium tuberculosis/africanum*	101	1079460	1007295	95924	82	4335752
S19BD02959	*Mycobacterium tuberculosis/africanum*	76	958367	880654	81627	120	4340532
S19BD02961	*Mycobacterium tuberculosis/africanum*	86	1032148	955187	81412	101	4337453
S19BD02962	*Mycobacterium tuberculosis/africanum*	83	979697	910903	70921	117	4334452
S19BD03086	*Mycobacterium tuberculosis/africanum*	68	776973	707882	80541	113	4312483
S19BD03152	*Mycobacterium tuberculosis/africanum*	61	670236	620391	75783	113	4315442
S19BD03189	*Mycobacterium tuberculosis/africanum*	84	902004	840976	69392	112	4343756
S19BD03237	*Mycobacterium tuberculosis/africanum*	44	535349	489072	78317	129	4402857
S19BD03238	*Mycobacterium tuberculosis/africanum*	68	749774	698115	80266	121	4343533
S19BD03239	*Mycobacterium tuberculosis/africanum*	47	615948	583566	64744	133	4332635

S19BD03271	*Mycobacterium bovis/BCG*	160	1852302	1674155	81896	98	4270723
S19BD03401	*Mycobacterium tuberculosis/africanum*	88	1036067	953435	78157	109	4346348
S19BD03408	*Mycobacterium tuberculosis/africanum*	65	733919	673991	68413	135	4320568
S19BD03443	*Mycobacterium tuberculosis/africanum*	248	2785435	2502731	97934	91	4309027
S19BD03569	*Mycobacterium tuberculosis/africanum*	63	653305	621445	64266	125	4328961
S19BD03607	*Mycobacterium tuberculosis/africanum*	59	656721	594525	83880	107	4330806
S19BD03617	*Mycobacterium tuberculosis/africanum*	115	1311087	1202036	84010	96	4356914
S19BD03619	*Mycobacterium tuberculosis/africanum*	26	404815	381575	48611	184	4236117
S19BD03659	*Mycobacterium tuberculosis/africanum*	76	882993	793923	82450	102	4336871
S19BD03692	*Mycobacterium tuberculosis/africanum*	74	881218	794019	80643	110	4291722
S19BD03871	*Mycobacterium tuberculosis/africanum*	113	1246051	1160541	103385	92	4334112
S19BD04031	*Mycobacterium tuberculosis/africanum*	75	931574	865342	68834	112	4379958
S19BD04034	*Mycobacterium tuberculosis/africanum*	50	662925	623062	36573	218	4295040
S19BD04122	*Mycobacterium bovis/BCG*	87	940159	893371	81705	103	4266813
S19BD04317	*Mycobacterium tuberculosis/africanum*	102	1178220	1113081	71661	119	4343919
S19BD04437	*Mycobacterium tuberculosis/africanum*	90	1011179	943763	77531	109	4348086
S19BD04454	*Mycobacterium tuberculosis/africanum*	78	906423	853242	64663	138	4382209
S19BD04462	*Mycobacterium tuberculosis/africanum*	99	1142675	1054244	88318	142	4409141
S19BD04840	*Mycobacterium tuberculosis/africanum*	49	527971	477701	70728	123	4319140
S19BD04941	*Mycobacterium tuberculosis/africanum*	65	727526	673910	88706	105	4336346
S19BD04942	*Mycobacterium tuberculosis/africanum*	96	1008282	950932	80532	99	4346070
S19BD04943	*Mycobacterium tuberculosis/africanum*	75	795687	719737	98875	102	4332352
S19BD05114	*Mycobacterium bovis/BCG*	62	630133	597575	72529	111	4281033
S19BD05219	*Mycobacterium tuberculosis/africanum*	104	1176449	1112116	79207	103	4334118
S19BD05549	MTBC	52	598970	545393	75456	133	4378469
S19BD05637	*Mycobacterium bovis/BCG*	84	954790	888270	92234	107	4279278
S19BD05638	*Mycobacterium tuberculosis/africanum*	69	800631	749947	76067	111	4327044
S19BD05639	*Mycobacterium tuberculosis/africanum*	57	756897	678168	70524	125	4344342
S19BD05777	*Mycobacterium tuberculosis/africanum*	93	1026203	956006	103370	97	4337595
S19BD05778	*Mycobacterium tuberculosis/africanum*	82	905732	843510	82311	97	4324178
S19BD06004	*Mycobacterium tuberculosis/africanum*	75	895735	777654	71132	121	4304308
S19BD06016	*Mycobacterium tuberculosis/africanum*	82	862399	802034	82502	111	4345976
S19BD06018	*Mycobacterium bovis/BCG*	49	661825	568510	60789	129	4258771

S19BD06295	*Mycobacterium tuberculosis/africanum*	72	803957	744735	81870	107	4347018
S19BD06296	*Mycobacterium tuberculosis/africanum*	82	935100	875321	65683	128	4407897
S19BD06838	*Mycobacterium tuberculosis/africanum*	64	781876	738501	80602	116	4350936
S19BD07348	*Mycobacterium tuberculosis/africanum*	123	1285388	1199947	95925	96	4347270
S19BD07397	*Mycobacterium tuberculosis/africanum*	58	620148	581974	82199	109	4297272
S19BD07681	*Mycobacterium bovis/BCG*	87	938761	881193	68252	121	4258903
S19BD07775	*Mycobacterium tuberculosis/africanum*	82	904593	847210	82509	120	4350156
S19BD07878	*Mycobacterium tuberculosis/africanum*	57	611768	573262	68031	116	4344133
S19BD07881	*Mycobacterium tuberculosis/africanum*	58	624927	577285	70425	119	4313632
S19BD07893	*Mycobacterium tuberculosis/africanum*	53	617963	561660	65849	122	4312974
S19BD07950	*Mycobacterium tuberculosis/africanum*	79	903811	845964	76316	102	4349564
S19BD08025	*Mycobacterium tuberculosis/africanum*	58	702759	627083	65380	116	4351343
S19BD08414	*Mycobacterium tuberculosis/africanum*	67	815856	763600	76937	115	4319426
S19BD08608	*Mycobacterium tuberculosis/africanum*	87	1119823	1025744	83444	112	4344255
S19BD08697	*Mycobacterium tuberculosis/africanum*	91	1021512	938530	74693	105	4339968
S19BD08985	*Mycobacterium bovis/BCG*	65	746085	689667	75967	111	4295543
S19BD09037	*Mycobacterium tuberculosis/africanum*	51	613058	561008	71024	111	4338054
S19BD09173	*Mycobacterium tuberculosis/africanum*	45	512016	474645	71391	122	4337815
S19BD09201	*Mycobacterium tuberculosis/africanum*	66	733736	679136	70600	131	4376857
S19BD09267	*Mycobacterium tuberculosis/africanum*	90	986163	932616	66354	116	4392640
S19BD09360	*Mycobacterium tuberculosis/africanum*	78	889986	842130	80286	117	4394026
S19BD09501	*Mycobacterium tuberculosis/africanum*	58	612331	556560	78322	117	4320579
S20BD00190	*Mycobacterium tuberculosis/africanum*	53	567640	542416	72254	123	4340620
S20BD00314	*Mycobacterium tuberculosis/africanum*	90	991698	921215	86641	100	4337147
S20BD00315	*Mycobacterium bovis/BCG*	80	834458	795324	58383	142	4241267
S20BD00328	*Mycobacterium tuberculosis/africanum*	117	1514660	1419156	82251	97	4351250
S20BD00569	*Mycobacterium tuberculosis/africanum*	61	705161	656974	79282	113	4357628
S20BD00570	*Mycobacterium tuberculosis/africanum*	60	645287	573238	82263	102	4322355
S20BD00934	*Mycobacterium tuberculosis/africanum*	76	812171	762114	82604	112	4348257
S20BD00953	*Mycobacterium bovis/BCG*	97	993269	936528	76156	112	4266138
S20BD01134	*Mycobacterium tuberculosis/africanum*	75	783894	721726	76956	110	4316923
S20BD01357	*Mycobacterium tuberculosis/africanum*	79	830868	790842	71847	114	4362871
S20BD01537	*Mycobacterium tuberculosis/africanum*	84	874343	808527	78488	106	4347553

Table S16: Comparison predicted and observed AMR phenotypes.

Observations were evaluated at the level on the antibiotic for each sample for which AMR phenotypic data was available (see Supplementary Table S4). The following definitions were used: TP as observed and predicted resistance, FP as observed susceptibility and predicted resistance, TN as predicted and observed susceptibility, and FN as predicted susceptibility and observed resistance. Dashes indicate the corresponding combination of antibiotic and sample were not tested. Ofloxacin and moxifloxacin were compared with the predicted resistance to fluoroquinolones.

	First Line		Second line (group A)		Second line (group B)		Second line (group C)	Second line (group D)	Other					
**sample**	**Isoniazid**	**Rifampicin**	**Ofloxacin**	**Moxifloxacin**	**Amikacin**	**Capreomycin**	**Ethionamide**	**Pyrazinamide**	**Ethambutol**	**Rifabutine**	**PAS**	**Linezolid**	**Clofazimine**	**Bedaquiline**
S17BD04916	TP	TN	TN	TN	TP	TP	FN	TP	TP	TP	TN	TN	TN	TN
S18BD09410	TN	TN	-	-	-	-	-	TN	TN	-	-	-	-	-
S18BD09709	TP	TN	TN	TN	TN	TN	TN	FN	TP	TP	TN	TN	FN	TN
S19BD00079	TN	TN	-	-	-	-	-	TN	TN	-	-	-	-	-
S19BD00094	TN	TN	-	-	-	-	-	TN	TN	-	-	-	-	-
S19BD00140	TN	TN	-	-	-	-	-	TN	TN	-	-	-	-	-
S19BD00141	TN	TN	-	-	-	-	-	TN	TN	-	-	-	-	-
S19BD00168	TN	TN	-	-	-	-	-	TP	TN	-	-	-	-	-
S19BD00200	TN	TN	TN	TN	-	-	-	TN	TN	-	-	-	-	-
S19BD00282	TN	TN	-	-	-	-	-	TN	TN	-	-	-	-	-
S19BD00357	TN	TN	-	-	-	-	-	TN	TN	-	-	-	-	-
S19BD00373	TN	TN	-	-	-	-	-	TN	TN	-	-	-	-	-
S19BD00393	TN	TN	-	-	-	-	-	TN	TN	-	-	-	-	-
S19BD00444	TN	TN	-	-	-	-	-	TN	TN	-	-	-	-	-
S19BD00457	TN	TN	-	-	-	-	-	TN	TN	-	-	-	-	-
S19BD00561	TN	TN	-	-	-	-	-	TN	TN	-	-	-	-	-
S19BD00646	TP	TN	-	-	-	-	-	TN	TN	-	-	-	-	-
S19BD00752	TN	TN	-	-	-	-	-	TN	TN	-	-	-	-	-
S19BD00777	TN	TN	-	-	-	-	-	TN	TN	-	-	-	-	-
S19BD00819	TN	TN	-	-	-	-	-	TP	TN	-	-	-	-	-
S19BD00825	TN	TN	-	-	-	-	-	TN	TN	-	-	-	-	-
S19BD00844	TN	TN	-	-	-	-	-	TP	TN	-	-	-	-	-
S19BD00845	TN	TN	-	-	-	-	-	TN	TN	-	-	-	-	-
S19BD00846	TN	-	TN	TN	TN	TN	TN	-	-	TN	TN	TN	TN	TN
S19BD00856	TN	TN	-	-	-	-	-	TN	TN	-	-	-	-	-
S19BD00936	TN	TN	-	-	-	-	-	TP	TN	-	-	-	-	-
S19BD01003	TN	TN	-	-	-	-	-	TN	TN	-	-	-	-	-
S19BD01027	TN	TN	-	-	-	-	-	TN	TN	-	-	-	-	-
S19BD01090	TN	TN	-	-	-	-	-	TN	TN	-	-	-	-	-
S19BD01123	TN	TN	-	-	-	-	-	TN	TN	-	-	-	-	-
S19BD01129	TN	TN	-	-	-	-	-	TN	TN	-	-	-	-	-
S19BD01163	TN	TN	-	-	-	-	-	TN	TN	-	-	-	-	-

S19BD01204	TN	TN	-	-	-	-	-	TN	TN	-	-	-	-	-
S19BD01217	TP	TN	TN	TN	TN	TN	TP	TP	FP	TP	TN	TN	TN	TN
S19BD01316	TP	TN	-	-	-	-	-	TN	TN	-	-	-	-	-
S19BD01376	-	-	-	-	-	-	-	TN	-	-	-	-	-	-
S19BD01410	TN	TN	-	-	-	-	-	TN	TN	-	-	-	-	-
S19BD01457	TN	TN	-	-	-	-	-	TN	TN	-	-	-	-	-
S19BD01466	TN	TN	-	-	-	-	-	TN	TN	-	-	-	-	-
S19BD01492	TN	FP	TN	TN	TN	TN	TN	FN	TN	TN	TN	TN	FN	TN
S19BD01544	TN	TN	-	-	-	-	-	TN	TN	-	-	-	-	-
S19BD01586	TN	TN	-	-	-	-	-	TN	TN	-	-	-	-	-
S19BD01610	TN	TN	-	-	-	-	-	TN	TN	-	-	-	-	-
S19BD01633	TN	TN	-	-	-	-	-	TN	TN	-	-	-	-	-
S19BD01643	TN	TN	-	-	-	-	-	TN	TN	-	-	-	-	-
S19BD01749	TN	TN	-	-	-	-	-	TN	TN	-	-	-	-	-
S19BD01779	TN	TN	-	-	-	-	-	TN	TN	-	-	-	-	-
S19BD01836	TN	TN	-	-	-	-	-	TN	TN	-	-	-	-	-
S19BD01876	TP	TN	-	-	-	-	-	TN	TN	-	-	-	-	-
S19BD01938	TN	TN	-	-	-	-	-	TN	TN	-	-	-	-	-
S19BD01939	TN	TN	-	-	-	-	-	TN	TN	-	-	-	-	-
S19BD02068	-	-	TN	TN	-	-	-	-	-	-	-	-	-	-
S19BD02087	TN	TN	-	-	-	-	-	TN	TN	-	-	-	-	-
S19BD02149	TN	TN	-	-	-	-	-	TN	TN	-	-	-	-	-
S19BD02152	TN	TN	-	-	-	-	-	TN	TN	-	-	-	-	-
S19BD02160	TN	TN	TN	TN	TN	TN	TN	TN	TN	TP	TN	TN	TN	TN
S19BD02203	TN	TN	-	-	-	-	-	TN	TN	-	-	-	-	-
S19BD02236	TP	-	TN	TN	-	-	-	-	-	-	-	-	-	-
S19BD02237	-	-	TN	TN	-	-	-	-	-	-	-	-	-	-
S19BD02272	TP	TP	TN	TN	TN	TN	TN	TN	TN	TP	TP	TN	FN	TN
S19BD02311	TN	TN	-	-	-	-	-	TN	TN	-	-	-	-	-
S19BD02343	TN	TN	-	-	-	-	-	TN	TN	-	-	-	-	-
S19BD02344	TN	TN	-	-	-	-	-	TN	TN	-	-	-	-	-
S19BD02390	TN	TN	-	-	-	-	-	TN	TN	-	-	-	-	-
S19BD02394	TN	TN	-	-	-	-	-	FN	TN	-	-	-	-	-
S19BD02423	TN	TN	-	-	-	-	-	TP	TN	-	-	-	-	-
S19BD02475	TN	TN	-	-	-	-	-	TN	TN	-	-	-	-	-
S19BD02535	TN	TN	-	-	-	-	-	TN	TN	-	-	-	-	-
S19BD02579	TN	TN	-	-	-	-	-	TP	TN	-	-	-	-	-
S19BD03569	TP	TN	-	TP	TN	TN	FN	TN	TN	TP	TN	TN	TN	TN
S19BD03692	TN	TN	-	-	-	-	-	TN	TN	-	-	-	-	-
S19BD04122	TN	TN	-	-	-	-	-	TP	TN	-	-	-	-	-
S19BD04454	TP	TN	-	-	-	-	-	TN	TN	-	-	-	-	-
S19BD04840	TP	TN	TN	TN	TN	TN	FN	FN	TP	TN	TN	TN	TN	TN
S19BD05549	TN	TN	-	-	-	-	-	TN	TN	-	-	-	-	-
S19BD05638	TP	TN	TN	TN	TN	TN	FN	TP	TP	TN	TN	TN	TN	TN
S19BD05639	TP	TN	TN	TN	TN	TN	TN	TN	FP	TP	TP	TN	TN	TN
S19BD06004	FN	TN	TN	TN	TN	TN	-	TN	TN	TN	TN	TN	TN	TN
S19BD06295	TP	TN	TN	TN	TN	TN	FN	TN	TN	TN	TN	TN	TN	TN
S19BD06296	TP	TN	TN	TN	TN	TN	TN	TN	TN	TN	TN	TN	TN	TN
S19BD07681	TN	TN	-	-	-	-	-	TP	TN	-	-	-	-	-
S19BD07775	TP	TN	TN	TN	TN	TN	TP	TP	TP	TP	TN	TN	TN	TN
S19BD07881	TP	TP	TN	TN	TN	TN	TN	TN	TN	TP	TN	TN	TN	TN
S19BD07950	TP	TN	TN	TN	TN	TN	TP	TN	TN	TN	TN	TN	FN	TN
S19BD09173	-	-	-	-	-	-	-	TN	-	-	-	-	-	-
S19BD09201	TP	TP	TN	TN	TN	TN	FN	TP	FP	TP	TN	TN	FN	TN

Figures

Figure S1: Example output of the region of difference (RD) *csb* species determination assay.

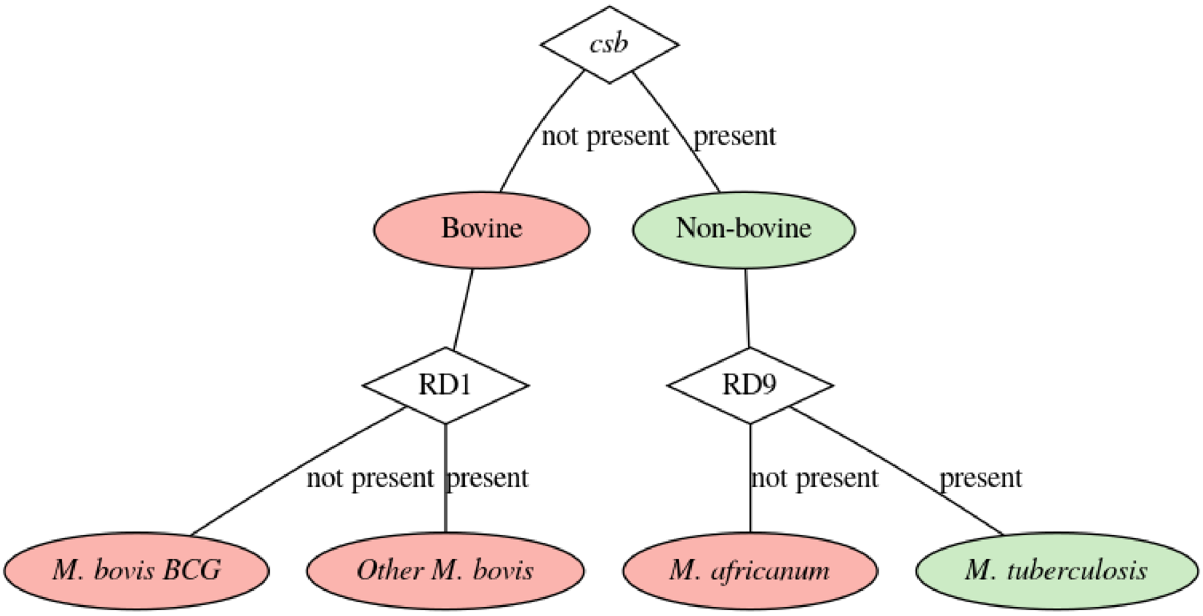

Figure S2: Examples of SNP-based antimicrobial resistance detection visualization.

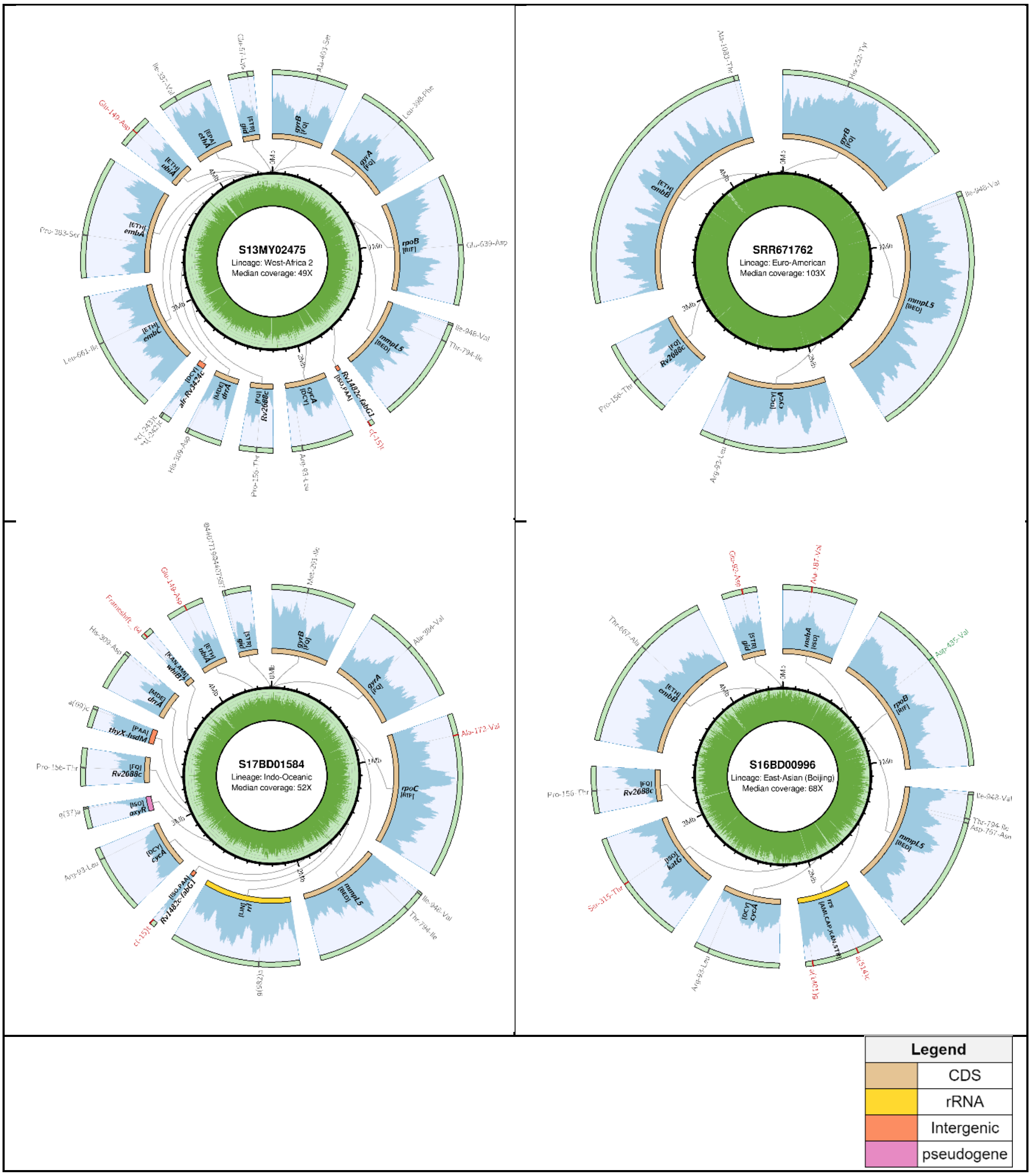

The sample name, lineage (based on SNP-barcoding) and median coverage against the H37Rv reference genome are listed in the center of the plot. The coverage against the reference genome is visualized in the inner ring (on a scale from 0 to 100x). Regions containing mutations classified as either 'known resistant' or 'unknown' are highlighted as slices with the inner bands of the slices indicating the type of region (see legend). The dark blue indicates the relative coverage within the region, and mutations are visualized with centripetal lines, with 'known resistant' shown in red and 'unknown' mutations in grey. Mutations that did not pass variant filtering are prefixed with an asterisk (*). The

antibiotic(s) associated with the regions are indicated within brackets. Abbreviations: bedaquiline (BED), D-cycloserine (DCY), ethionamide and prothionamide (EPA), ethambutol (ETH), fluoroquinolone (FQ), isoniazid (ISO), kanamycin (KAN), pyrazinamide (PYR), rifampicin (RIF), and streptomycin (STR).

Figure S3: Rv1482c-*fabG1* c(-15)t mutation in sample S16BD06161 reported as *wt* by Sanger sequencing.

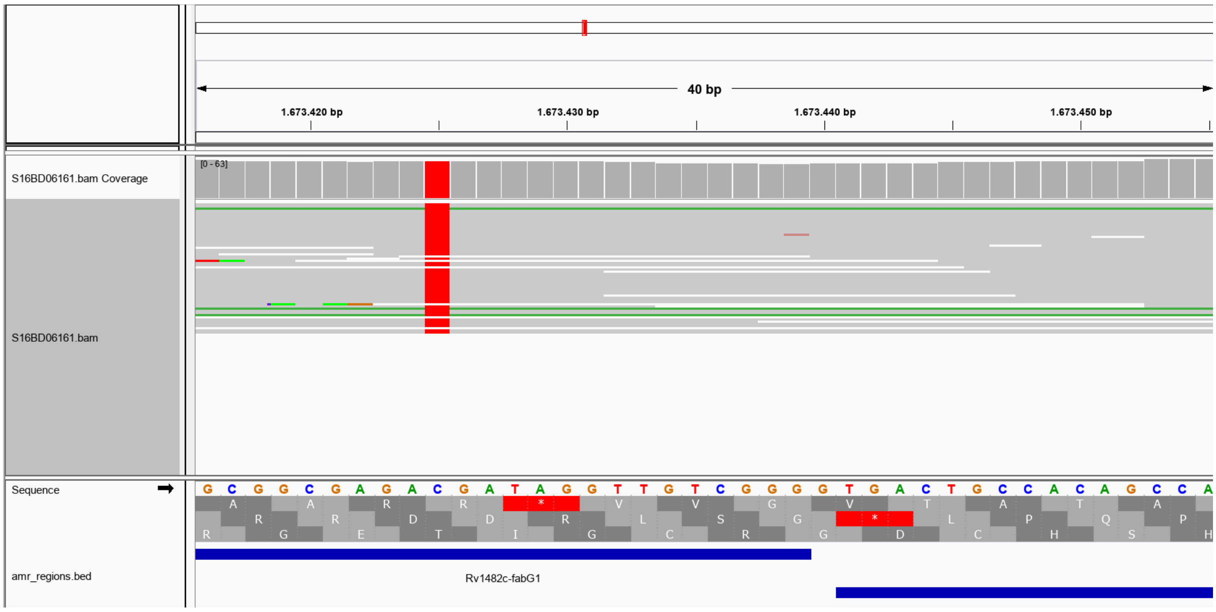

The mutation at position -15 in the promotor region is shown in red (corresponding to nucleotide T), and is present in all reads aligned to this position. The wild type sequence from the H37Rv reference genome is shown at the bottom of the plot. This sample also contained five mismatches for spacers in the spoligotyping when comparing with the reference data (spacers 10, 20, 21, 22, and 35), more than any other sample. These two observations are consistent with the hypothesis of a sample swap.

Figure S4: Pre-existing stop-codons and frameshifts observed in the *in silico* modified datasets for the SNP-based antimicrobial resistance detection assay validation

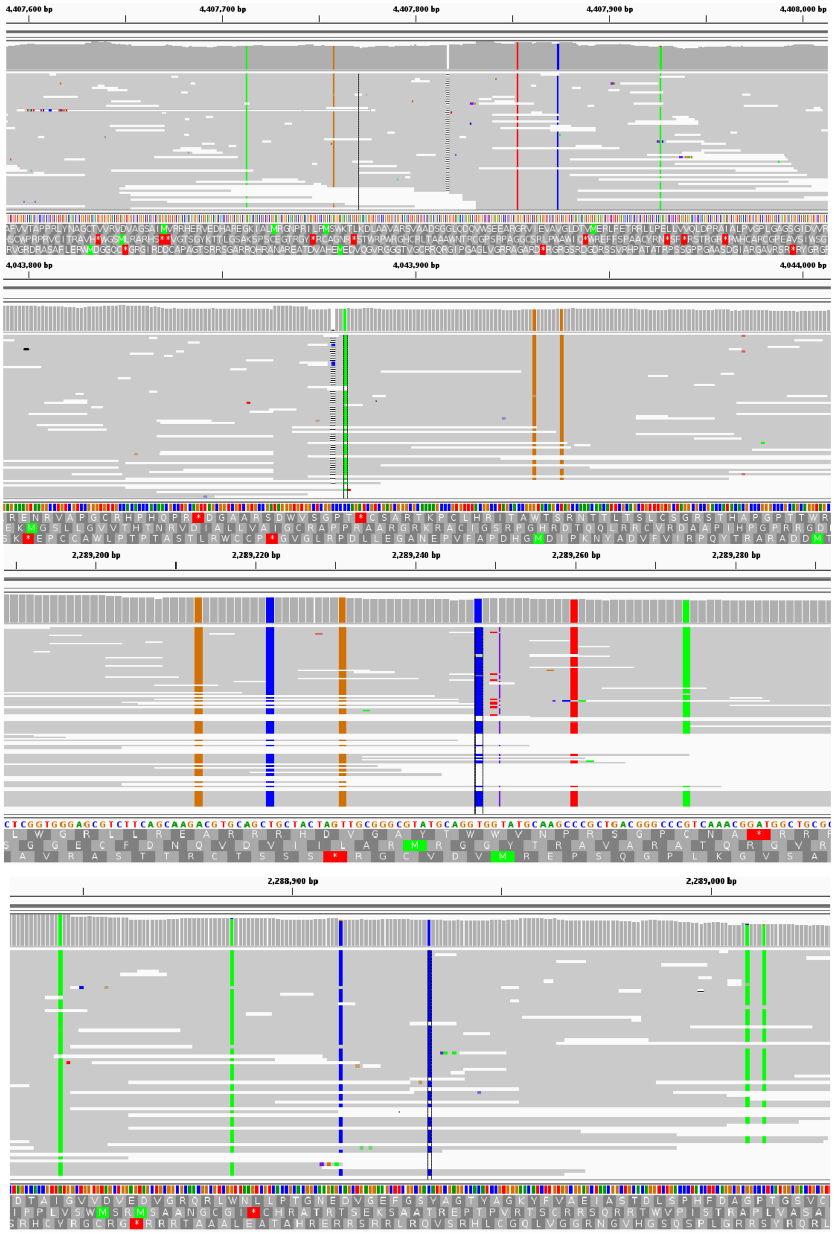

First and second panel: two pre-existing deletions causing frameshifts, respectively upstream of affected mutations (position 4,407,817) and immediately downstream of an affected mutation (position 4,043,879) (reading frames are from right to left). Third panel: pre-existing insertion causing a frameshift upstream of affected mutations (position 2,289,250). Fourth panel: pre-existing stop codon (position 2,288,933). The '--local-csq' option from Bcftools csq can be used to perform localized predictions (i.e. considering the effect of mutations in isolation). While enabling this option would results in less FNs in the validation, predictions would be less accurate at the phenotypic level, since mutations upstream of stop codons or frameshift are not expressed *in vitro*.
